# Transcript Profile of Flowering Regulatory Genes in *VcFT*-Overexpressing Blueberry Plants

**DOI:** 10.1371/journal.pone.0156993

**Published:** 2016-06-07

**Authors:** Aaron E. Walworth, Benli Chai, Guo-qing Song

**Affiliations:** 1 Plant Biotechnology Resource and Outreach Center, Department of Horticulture, Michigan State University, East Lansing, Michigan, United States of America; 2 Center for Microbial Ecology, Michigan State University, East Lansing, Michigan, United States of America; Clemson University, UNITED STATES

## Abstract

In order to identify genetic components in flowering pathways of highbush blueberry (*Vaccinium corymbosum* L.), a transcriptome reference composed of 254,396 transcripts and 179,853 gene contigs was developed by assembly of 72.7 million reads using Trinity. Using this transcriptome reference and a query of flowering pathway genes of herbaceous plants, we identified potential flowering pathway genes/transcripts of blueberry. Transcriptome analysis of flowering pathway genes was then conducted on leaf tissue samples of transgenic blueberry cv. Aurora (‘VcFT-Aurora’), which overexpresses a blueberry *FLOWERING LOCUS T*-like gene (*VcFT*). Sixty-one blueberry transcripts of 40 genes showed high similarities to 33 known flowering-related genes of herbaceous plants, of which 17 down-regulated and 16 up-regulated genes were identified in ‘VcFT-Aurora’. All down-regulated genes encoded transcription factors/enzymes upstream in the signaling pathway containing *VcFT*. A blueberry *CONSTANS*-*LIKE 5*-like (*VcCOL5*) gene was down-regulated and associated with five other differentially expressed (DE) genes in the photoperiod-mediated flowering pathway. Three down-regulated genes, *i*.*e*., a *MADS-AFFECTING FLOWERING 2*-like gene (*VcMAF2*), a *MADS-AFFECTING FLOWERING 5*-like gene (*VcMAF5*), and a *VERNALIZATION1*-like gene (*VcVRN1*), may function as integrators in place of *FLOWERING LOCUS C* (*FLC*) in the vernalization pathway. Because no *CONSTAN1*-like or *FLOWERING LOCUS C*-like genes were found in blueberry, *VcCOL5* and *VcMAF2*/*VcMAF5* or *VRN1* might be the major integrator(s) in the photoperiod- and vernalization-mediated flowering pathway, respectively. The major down-stream genes of *VcFT*, *i*.*e*., *SUPPRESSOR of Overexpression of Constans 1*-like (*VcSOC1*), *LEAFY*-like (*VcLFY*), *APETALA1*-like (*VcAP1*), *CAULIFLOWER 1*-like (*VcCAL1*), and *FRUITFULL*-like (*VcFUL*) genes were present and showed high similarity to their orthologues in herbaceous plants. Moreover, overexpression of *VcFT* promoted expression of all of these *VcFT* downstream genes. These results suggest that *VcFT’s* down-stream genes appear conserved in blueberry.

## Introduction

Blueberry contains high amounts of antioxidants known to be important for human health [1]. The highbush blueberry (2n = 4x = 48) (*Vaccinium corymbosum* L.), including northern and southern ecotypes, is the major cultivated *Vaccinium* fruit crop [2, 3]. The northern highbush cultivars have better winter hardiness, but their adoption in warm areas is often limited by their requirement for more chill units (CU) (generally > 800) to break dormancy in the spring. In contrast, the southern highbush blueberry cultivars are derivatives of the northern highbush blueberry with additional genes from other southern *Vaccinum* species [*e*.*g*., *V*. *darrowi* (2n = 2x = 24), *V*. *ashei* (2n = 6x = 72), and *V*. *tenellum* (2n = 2x = 24)] [2]. These southern cultivars have better summer heat tolerance and require fewer chill units (150 to 600 CU) to induce flowering, but they are often less cold/freeze-tolerant. Developing new cultivars with different flowering times and chilling requirements, high cold/heat tolerances, and high yields are the top priorities in breeding for sustainable blueberry production, particularly in anticipation of climate changes.

Abundant genetic resources of the genus *Vaccinium* (*i*.*e*. 450 species) contain high genetic diversity but also lead to great complexity making it difficult to improve the cultivated tetraploid highbush cultivars through conventional breeding [2]. Alternatively, new genomic and biotechnological tools will facilitate studies on blueberry genetics and genomics and improve breeding efficiency [2, 4–10]. For blueberry genomic studies, a draft blueberry genome assembly with 358 million base pairs (Mb) of diploid blueberry accession ‘W8520’ was generated and used for genetic diversity studies of cultivated blueberry cultivars as well as marker-assisted breeding and comparative genomics of *Vaccinium* species [2]. Using RNA-Seq data and a draft blueberry genome assembly, candidate genes involved in fruit ripening and biosynthesis of bioactive compounds have been reported [7]. In addition, 22,401 blueberry (*V*. *corymbosum*) expressed sequence tags (ESTs) are available in GenBank. Generation and analyses of the transcriptomes of flower and fruit tissues of *V*. *corymbosum* ‘Northland’ yielded 34,464 NCBI Unigene clusters out of 64 million sequencing reads for identifying genes involved in antioxidant biosynthesis [8]. Transcriptomes of the major highbush blueberry cultivar Bluecrop were generated from leaves, flower buds at different stages of cold acclimation, and fruit tissues at different stages of development. About 15,000 contigs were derived from 600,000 reads and are considered to be useful for analyzing differentially expressed (DE) genes in flower bud formation, vernalization, cold acclimation, and fruit development [9].

Reliable and highly effective *Agrobacterium tumefaciens*-mediated transformation and regeneration methods have been developed for several highbush blueberry cultivars [11, 12]. The availability of blueberry transcriptome information and genetic transformation tools enables gene cloning and functional gene analysis. For example, overexpressing the endogenous blueberry C-repeat binding factor (CBF) gene (*VcCBF*) (GenBank AF234316) resulted in a significant increase of freeze tolerance in leaves and floral buds [10]. These results demonstrate the potential of genomics and genetic transformation for blueberry breeding.

Flowering is controlled and modulated by flowering genes and environmental signals (*e*.*g*., light and temperature) and is of importance for sustainable blueberry production. To date, the molecular basis of flowering in *Arabidopsis thaliana* (L.) and other herbaceous plants has been extensively studied through investigations into the networks of flowering-related genes [13–21]. In contrast, few similarly detailed studies have been conducted to reveal genetic control of seasonal flowering of woody perennials [14, 22]. Of the reported flowering-related genes of *A*. *thaliana*, the major ones include *CONSTANS 1* (*CO1*), *FLOWERING LOCUS C* (*FLC*), *FLOWERING LOCUS T* (*FT*), *SUPPRESSOR of Overexpression of Constans 1* (*SOC1*), *LEAFY* (*LFY*), *APETALA1* (*AP1*), and *FRUITFULL* (*FUL*) [23]. To study the vernalization-mediated flowering mechanisms in blueberry, we isolated one blueberry *FT*-like gene (*VcFT*), three *AP1*-like genes, three *SOC1*-like genes and one *LFY*-like gene (*VcLFY*) from the highbush blueberry cvs. Bluecrop and Legacy. Overexpressing *VcFT* caused early and continuous flowering in *in vitro* shoots and in one-year old ‘Aurora’ plants [5]. However, our observation on two- and three-year old plants showed that the *VcFT*-overexpressing plants did not flower normally and the majority of the flower buds did not open under greenhouse conditions without chilling. Analyses of DE genes in *VcFT*-overexpressing blueberry plants should reveal *VcFT*-regulated flowering gene networks.

The aim of this study is to identify flowering-related gene networks of highbush blueberry. We developed a transcriptome reference of blueberry, assembled using Trinity [24], and annotated flowering pathway genes by comparative genomics of *A*. *thaliana*, rice (*Oryza sativa*), and some cereals, were used to identify flowering-related genes of blueberry [25]. DE analysis of flowering-related genes was subsequently conducted on the transcriptome from leaf tissues of transgenic ‘Aurora’ plants (herein ‘VcFT-Aurora’) overexpressing *VcFT*. In this way, we expected to identify functional flowering pathway genes of blueberry, which respond to the major flowering pathway integrator *VcFT*.

## Materials and Methods

### Plant materials

The northern highbush blueberry cv. Aurora and the southern highbush cv. Legacy were used. Both cultivars are tetraploid and require at least 800 CU to flower. The reference transcriptome was developed using eight ‘Legacy’ tissue samples derived from a wild type plant, one plant from each of two transgenic events containing an overexpressed *VcCBF* (herein ‘VcCBF-Legacy’) obtained in our previous study [10]. These tissue samples included unvernalized leaves and floral buds and flower tissues with corollas removed. Three plants of wild type ‘Aurora’ and three plants from one transgenic line of ‘VcFT-Aurora’, a transgenic line overexpressing *VcFT* obtained in our previous study [5], were used to analyze differential expression resulting from *VcFT* overexpression. Differential expression comparisons across tissue types (leaves, floral buds, and flower) utilized three plants of wild type ‘Legacy’.

Growth and flowering of non-transgenic ‘Aurora’ and transgenic ‘VcFT-Aurora’ plants were investigated using 12 non-transgenic plants and 12 plants from each of the five transgenic events obtained in our previous study [5]. Phenotypic data (*i*.*e*., plant height, flowering time, the number of floral buds that flowered, and flower number per flowered bud) were collected. For differential expression analyses, young leaf tissues of 3 two-year old, unvernalized ‘Aurora’ plants and 1 transgenic ‘VcFT-Aurora’ event of were sampled for RNA isolation and sequencing.

All plants were from *in vitro* cultured shoots and grown in the greenhouse (heated for winter) under natural light and ambient temperature conditions, and a regular schedule of irrigation and fertilization using 0.2 g/L fertilizer (Nitrogen: Phosphorus: Potassium = 21: 7: 7) [11]. To get full vernalization, one-year old plants were grown in controlled environment chambers at 4°C with a 12 hour photoperiod for two months; two- and three-year old plants were exposed to the natural environment in winter in a secured courtyard between our greenhouses.

### RNA preparation and sequencing

Floral buds were collected in November 2013 before the plants were exposed to a non-heated greenhouse for chilling. Newly emerged young leaf and flower tissues of ‘Legacy’ were collected in February 2014. Flower tissues consisted of all parts from newly opened flowers except corollas. Young leaf tissues of two-year old ‘Aurora’ and ‘VcFT-Aurora’ plants were collected in June 2014 from unvernalized plants. All tissues collected were frozen immediately in liquid nitrogen and stored at -80°C.

Total RNA was isolated from 0.5 g tissues for each sample, using a cetyltrimethylammonium bromide (CTAB) method [26]. The samples were purified using RNeasy Mini Kit and On-Column DNase digestion with the RNase-free DNase Set (Qiagen, Valencia, CA, USA). Integrity of the RNA samples was assessed using the Agilent RNA 6000 Pico Kit (Agilent Technologies, Inc. Germany). All samples had a RNA quality score above 8.0 before submission for sequencing (100-bp paired-end reads) using the Illumina HiSeq2500 platform at the Research Technology Support Facility of Michigan State University (East Lansing, MI, USA).

### *de novo* transcriptome assembly

FastQC program (http://www.bioinformatics.babraham.ac.uk/projects/fastqc/) was used to assess the quality of sequencing reads for the per base quality scores range from 26–40. Generation of the transcriptome reference for highbush blueberry from RNA-seq was carried out using the Trinity platform (trinity/20140413p1)[24]. The command for running about 72 million reads (MR) was “Trinity—seqType fq—JM 50G —left reads_R1_CBFVF.fq—right reads_R2_CBFVF.fq—SS_lib_type RF—CPU 24”. The memory request was 250G and the wall-time request was 48 hr. Left/Right reads were a combination of the reads from eight samples of non-transgenic and transgenic ‘Legacy’. The quality of the assembly was assessed by alignment with existing EST singlets and contigs in the Unigene v1 library obtained from www.vaccinium.org. These sequences were derived from a variety of tissues and cultivars of highbush blueberry. BLASTplus version 2.2.27 was used to run blastn with default parameters except dust was off, percent identity ranged from 80–95%, with an evalue threshold of 1e-05. Unigene sequences were used as query with refTrinity as database. Results were filtered for completeness, calculated as (qend-(qstart-1))/qlen.

### Differential expression analysis

RNA-seq reads of three biological replicates for each of the ‘Legacy’ tissues, plus ‘Aurora’ and ‘VcFT-Aurora’ leaves were analyzed. Two technical replicates were sequenced for each biological replicate and combined together for analysis. The paired reads, two sets for each biological replicate, were aligned to the transcriptome reference developed for ‘Legacy’ and the abundance of each read was estimated using the Trinity command “align_and_estimate_abundance.pl”. This generated 12 “genes.counts.matrix” and 12 “transcripts.counts.matrix” files. The Trinity command “abundance_estimates_to_matrix.pl—est_method RSEM” was then used to merge “genes.counts.matrix” files and “transcripts.counts.matrix” files, separately. The resultant two matrices were used for running DE analysis using the Trinity command “run_DE_analysis.pl—method edgeR”. The DE genes or transcripts with false discovery rate (FDR) values below 0.05 were used for further analyses of flowering pathway genes of blueberry.

For RT-PCR analysis of differentially expressed genes/transcripts, reverse-transcription of RNA to cDNA was performed using SuperScript II reverse transcriptase (Invitrogen, Carlsbad, CA, USA). The resulting cDNA was diluted (volume 1:10) in water, and 2 μl/sample, was used for each PCR reaction. Three RNA samples from leaf tissues for each of non-transgenic ‘Aurora’ and transgenic ‘VcFT-Aurora’ were used. The primers were included in S1 Table. The reaction conditions for all primer pairs were 94°C for 2 min, 35 cycles of 45 s at 94°C, 60 s at 60°C and 90 s at 72°C, with a final 10 min extension at 72°C. RT-PCR products were separated on 1.0% agarose gel containing ethidium bromide visualized, and photographed under UV light.

### Sequence sources of plant flowering pathway genes

Three protein datasets of the flowering pathway genes of *Arabidopsis*, rice, and cereal crops [*i*.*e*., bread wheat (*Triticum aestivum*), einkorn wheat (*T*. *monococcum*), barley (*Hordeum vulgare*), and maize (*Zea mays*)] were developed according to the gene IDs reported by [25] (S2 Table). Each dataset was used for orthologue identification using tblastn against the transcriptome reference of blueberry. The top hits (*i*.*e*., transcripts and genes) with e-values less than e-20, were retained to do comparative genomics of blueberry flowering genes and to search for the DE profiles of *VcFT*-overexpressing ‘Aurora’.

### Annotation of DE genes/transcripts

Trinotate was used to annotate the transcriptome reference and DE transcripts. Blueberry flowering pathway transcripts were also used as queries to search the *A*. *thaliana* protein database and GenBank using blastx. All Trinity and Trinotate analyses were performed using the resources at the High Performance Computing Center of Michigan State University.

## Results

### Phenotypes of *VcFT* overexpressing plants

**Fig 1:**
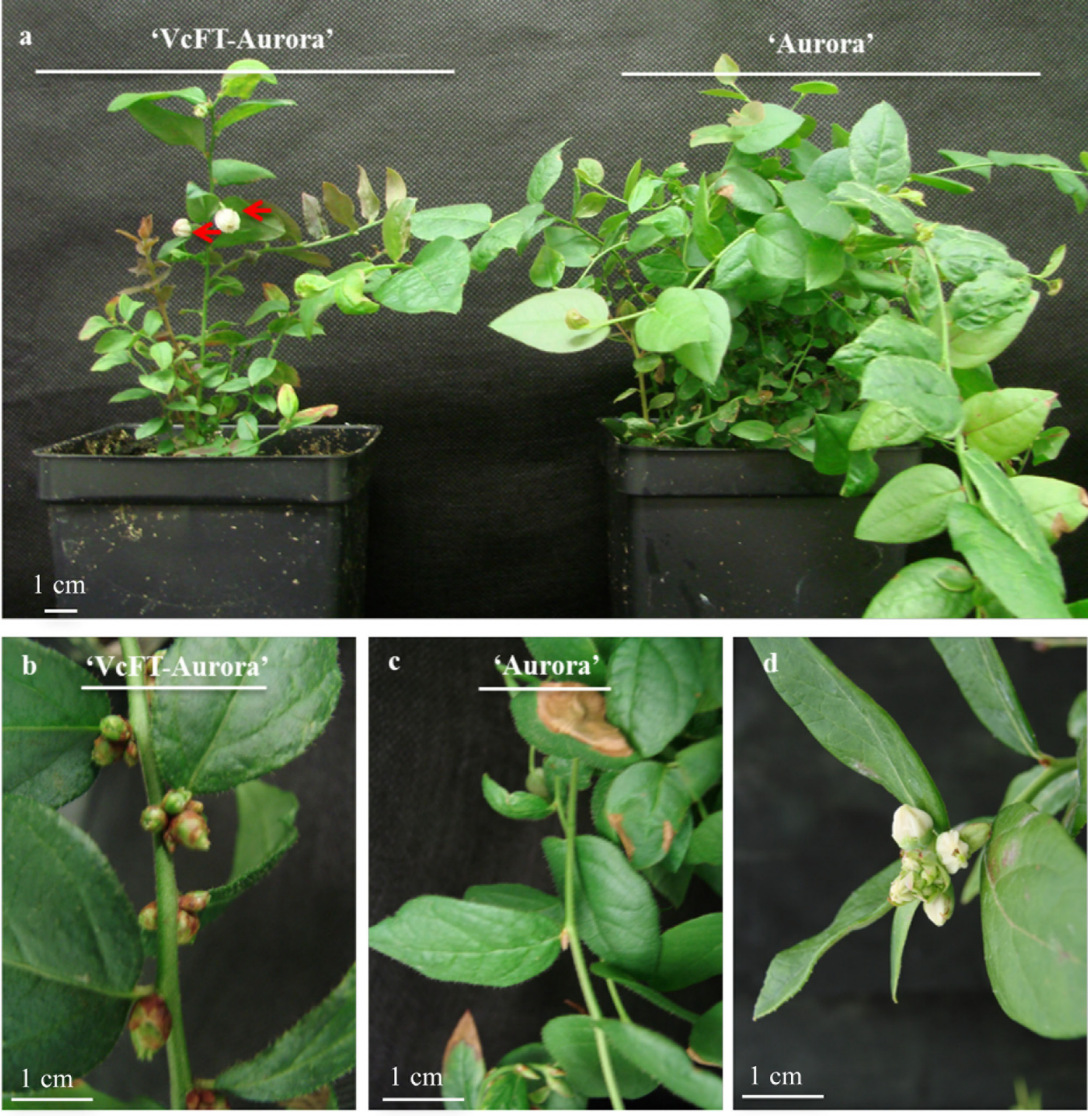
Comparison of flowering between ‘VcFT-Aurora’ and ‘Aurora’ plants. **a,** Continuous flowering of non-vernalized one-year old ‘VcFT-Aurora’ (left) alongside ‘Aurora’ plants with no flowers (right). Arrows show flowers. **b,** Floral bud formation on the new shoots of ‘VcFT-Aurora’. **c,** Vegetative buds, but not floral buds, appeared on new shoots of ‘Aurora’. **d,** Fully vernalized ‘VcFT-Aurora’ flowered normally.

‘VcFT-Aurora’ constitutively expresses *VcFT* driven by the cauliflower mosaic virus (CaMV) 35S promoter [5]. Overexpressing *VcFT* in ‘VcFT-Aurora’ resulted in dwarf plants, early floral bud formation, and continuous flowering (Fig 1A–1C). For example, the height of one- or two-year old ‘VcFT-Aurora’ plants was about half that of non-transgenic ‘Aurora’ and some newly formed buds flowered during the growing season (Fig 1A). ‘Aurora’ plants did not develop floral buds until they were three-years old. Fully chilled plants flowered with a bloom period of about one week and each bud had 5–10 flowers, while unvernalized plants did not flower. In contrast, ‘VcFT-Aurora’ shoots started to flower while they were in *in vitro* culture [5]. Fully chilled ‘VcFT-Aurora’ plants flowered normally with 5–10 flowers/bud (Fig 1D). The plants then formed new shoots and reverted to continuous floral bud formation and flowering. For unvernalized ‘VcFT-Aurora’ plants, 20–50% of floral buds flowered with 2–3 flowers/bud (Fig 1A). These results suggest that over-expressing *VcFT* alone promotes early flowering, but is not sufficient to completely substitute the need for vernalization for blueberries to flower normally under non-chilling conditions.

### Development of a transcriptome reference for blueberry

Approximately 8–10 million pair-end reads (MR) were generated for each sample. Using Trinity [24], 72.7 MR obtained from eight samples of leaf, floral bud, and flower tissues were assembled. The total Trinity ‘genes’ and ‘transcripts’ were 179,853 and 254,196, respectively (Table 1). All these transcript contigs, a total of 192 million bases, was designated as refTrinity and were used as the transcriptome reference for identifying candidate genes and differential expression analyses. The quality of the assembly was assessed by aligning to known EST and gene contig sequences obtained from the database at www.vaccinium.org. refTrinity aligned to 61% of 2,955 ESTs and 70% of 748 contig sequences with at least 90% identity and 90% completeness (S3 Table). Considering that the query sequences originated from varied cultivars, tissues, and treatments, the results suggest that Trinity is an efficient platform in generating transcriptome references for tetraploid blueberry plants.

**Table 1:** Statistic analysis of blueberry transcriptome reference developed using Trinity. Nt: nucleotide.

Statistics based on ALL transcript contigs	
Total trinity 'genes'	179,853
Total trinity transcripts	254,196
Percent GC	41.58
Contig N10	3323 nt
Contig N20	2528 nt
Contig N30	2018 nt
Contig N40	1620 nt
Contig N50	1256 nt
Median contig length	423 nt
Average contig	754 nt
Total assembled bases	191,637,363 nt

### Identification of flowering pathway genes of blueberry

Abundant sequence data (*i*.*e*., DNA, cDNA, EST, and protein) of flowering pathway genes from many plant species are deposited in GenBank. To identify orthologues of flowering pathway genes of blueberry, we used protein sequences derived from the previous reports and a cutoff e-value of above e-20 to identify flowering pathway genes in blueberry (S2 Table) [25]. When 156 sequences of 81 flowering-related genes of *Arabidopsis* (TAIR 10) were used to blast against refTrinity, 127 sequences of 57 genes got hits. Of the query of 160 sequences of 103 flowering pathway genes of rice (RGAP 7), only *OsF* gene (LOC_Os02g08150.1) did not have a match. With the query of 49 flowering-related genes of cereal crops, 48 genes had matching sequences. Overall, we pooled 1,223 transcript contigs from 720 gene contigs that showed high similarities (e-values ≤ e-20) to the query sequences, which allowed us to generate a reference of potential flowering pathway genes of blueberry (herein Trinity_floral_ref) (S4 Table).

Orthologues of most common flowering pathway genes of *Arabidopsis*, rice, and cereal crops were found in blueberry (S4 Table). These included, important genes such as *FRIGIDA*-like (*VcFRI*), *VcFT*, *APETALA 2*-like (*VcAP2*), *SUPPRESSOR of Overexpression of Constans 1*-like (*VcSOC1*), *LEAFY*-like (*VcLFY*), *APETALA1*-like (*VcAP1*), *CAULIFLOWER 1*-like (*VcCAL1*), and *FRUITFULL*-like (*VcFUL*) genes. These results validated refTrinity as a reliable transcriptome reference for identification of flowering pathway genes of blueberry.

*Arabidopsis CO1* and rice *HEADING DATE* (*HD1*) are the major integrator genes in their respective photoperiod-regulated flowering pathways [25]. Using the *CO1* as a query to search online *Vaccinium* sequences, we did not get any sequence with a high similarity. In Trinity_floral_ref, we found three *CONSTANS-LIKE* (*COL*)-like genes (*i*.*e*., *VcCOL2*, *VcCOL4*, and *VcCOL5*), but no *CO1*-like (*VcCO1*) or *HD1*-like genes.

*Arabidopsis FLC* and cereal *VRN(s)* and their orthologues are among the main integrators of vernalization pathway genes in herbaceous plants. In blueberry, we did not find *FLC* orthologues by searching the online *Vaccinium* sequences. In our Trinity_floral_ref, only one transcript, which has the best hit to *AGL79* (AT5G17690.1), showed 59.2% similarity (e-value = 2.00e-24) to *FLC* (AT5G10140.1). However, we found orthologues of other vernalization pathway genes, including a *VcFRI* (5.00e-58), a *MADS AFFECTING FLOWERING 2* (*MAF2*)-like gene (*VcMAF2*) (4.00e-33), a *MAF5*-like gene (*VcMAF5*) (2.00e-21)], a *VERNALIZATION 1*-like gene (*VcVRN1*), and a *VERNALIZATION 5*-like gene (*VcVRN5*). Of these genes, *VcVRN5* was the closest match (50.51% identity, e-value = 2.00e-168). These results suggest that blueberry (a perennial, woody plant) may have vernalization-pathways diverging from either the *FLC*-mediated pathway in *Arabidopsis* [27–29] or the *VERNALIZATION* genes (*VRNs*)-mediated pathways in cereals and their relatives [13, 16, 30]. Overall, of the major flowering pathway genes in ‘Aurora’, when compared to those in herbaceous plants, the genes up-stream of *VcFT* show less similarity than the down-stream genes (S4 Table).

### Tissue-specific expression of different flowering pathway genes

Comparative analyses of three pools of Trinity transcripts (*i*.*e*., leaf vs. floral bud, flower vs. floral bud, and leaf vs. flower) of non-transgenic blueberry plants showed that 1,193 out of 1,223 transcript contigs of the Trinity_floral_ref were found in the combined contigs of leaf, bud, and flower tissues of non-transgenic ‘Legacy’. Furthermore, 1,122 (out of 1,193) contigs were shared among three tissue types, of which 397 contigs showed no differential expression between the tissues. The remaining 725 transcripts (69 genes) showed differential expression in the comparisons of leaf vs. bud, flower vs. bud, or leaf vs. flower (Table 2). For example, of the photoperiod pathway genes, *VcCOL5* is a DE gene with high expression in leaf; but *VcCOL2* and *VcCOL4* did not show differential expression. Of the genes of the vernalization pathway, homologues of *MAF2*, *VRN1*, and *TaVRN1* showed the highest expression in leaf tissues and *VcVRN5* had the highest expression in buds; but *VcFRI* and *VcMAF5* were not differentially expressed in the comparisons (Table 2). The results indicate that expression of some flowering-related transcripts/genes in blueberry is tissue specific. In addition, RNA-seq transcriptome analysis provides a very efficient approach to confirm tissue specificity of gene expression.

**Table 2:** Differential expression of blueberry flowering pathway genes in leaf, bud, and flower tissues of ‘Legacy’.

Orthologues of flowering pathway genes	FPKM (fragments per kilobase of transcript per million mapped reads)			Gene ID	Orthologues of flowering pathway genes	FPKM (fragments per kilobase of transcript per million mapped reads)			Gene ID	Orthologues of flowering pathway genes	FPKM (fragments per kilobase of transcript per million mapped reads)		
	Bud	Leaf	Flower			Bud	Leaf	Flower			Bud	Leaf	Flower
ABF3	10.6	4.8	4.2	c99616_g2	CRY2	5.8	40.0	9.9	c92902_g3	PIE1	24.4	23.0	31.3
AGL14	3.2	45.8	0.0	c92516_g1	EFS	35.7	20.2	21.9	c95473_g3	PIE1	12.9	7.6	11.3
AGL19	4.9	20.5	5.6	c94437_g2	EFS	39.7	25.0	20.4	c95823_g6	PIE1	18.7	11.6	11.9
AGL19	129.5	85.1	15.2	c99784_g1	EFS	20.1	13.3	10.4	c96115_g2	PIE1	26.5	23.1	24.8
AGL19	2.9	19.0	4.7	c99653_g1	ELF3	22.8	27.9	47.1	c96482_g1	PIE1	27.4	15.4	12.8
AGL19	30.3	0.2	86.8	c93803_g1	ELF4	23.8	30.2	47.4	c97601_g1	PIE1	31.4	16.6	13.1
AGL19	17.1	72.0	2.0	c95520_g1	ELF4	6.4	8.9	4.9	c98765_g4	PIE1	23.0	16.5	20.0
AGL19	5.1	25.2	3.8	c91390_g4	ELF5	56.8	94.4	44.2	c99276_g1	PIE1	36.5	17.1	7.5
AP1	7.4	0.0	14.4	c97312_g2	ELF6	22.0	14.4	10.9	c99993_g3	PIE1	24.6	11.5	12.9
AP1	91.4	0.5	15.6	c98947_g5	ELF6	50.5	21.1	22.0	c98035_g1	PRR 7	87.0	26.6	28.8
AP2	12.7	5.1	3.2	c99119_g2	ELF6	28.6	17.4	14.1	c91063_g2	PRR5	2.0	0.3	1.3
AP2	56.4	32.8	40.3	c97265_g1	FCA	15.8	19.8	8.1	c92222_g2	PRR5	47.8	65.2	27.1
AREB3	35.9	18.8	18.6	c66694_g1	FD	5.9	3.7	0.1	c92704_g6	PRR5	53.4	2.2	0.8
ARP	11.7	10.0	27.0	c96634_g1	FKF1	2.1	3.8	5.3	c96565_g2	PRR5	77.8	51.0	32.8
ARP6	35.0	21.1	17.7	c96634_g2	FKF1	84.3	112.7	175.8	c94181_g4	RAV1	22.6	10.6	3.6
ARP6	382.0	437.9	397.7	c96828_g1	FKF1	66.0	31.4	11.0	c88293_g2	SOC1	5.5	27.0	0.0
ARP6	26.9	15.5	13.6	c97877_g1	FKF1	23.3	60.6	23.9	c89673_g5	SOC1	20.9	87.9	13.2
ARP6	76.4	133.2	212.4	c89277_g1	FLD	12.7	16.5	28.9	c99509_g2	SPA2	35.0	27.6	17.1
ATCOL5	29.6	10.6	16.8	c97418_g1	FLD	11.1	4.1	2.5	c97089_g4	SPA3	48.6	72.0	38.4
CAL1	0.4	0.6	14.2	c81479_g2	FLK	61.4	39.8	37.8	c79187_g1	SPL	104.7	34.4	23.0
CDF2	2.8	13.4	5.2	c91122_g1	FLK	39.0	25.0	20.9	c80807_g1	SPL	283.3	294.3	133.4
CDF2	25.8	37.1	26.6	c92308_g1	FLK	12.4	11.4	12.7	c87494_g1	SPL	4.7	3.8	6.4
CDF3	16.2	15.7	35.2	c97816_g1	FLK	44.5	28.0	25.7	c91703_g1	SPL	122.3	89.5	73.5
CDF3	1.8	3.9	1.1	c99051_g1	FPA	9.6	11.0	10.4	c92156_g2	SPL	64.1	48.0	24.9
CDF3	20.1	15.5	9.2	c99819_g1	FPA	20.4	15.5	12.4	c93310_g3	SPL	253.3	114.1	130.7
CDF3	57.9	22.5	11.4	c84088_g2	FT	34.9	0.2	0.9	c93493_g1	SPL	11.8	11.4	4.2
CHE	19.3	13.1	3.4	c75036_g1	FUL	93.7	24.6	66.1	c96319_g1	SPL	80.6	16.2	27.3
CHE	32.5	39.7	15.7	c77424_g2	FUL	4.8	0.0	54.8	c92144_g7	SPY	12.9	6.4	7.7
CHE	16.8	12.5	5.1	c79125_g1	FUL	120.5	14.7	38.5	c92144_g9	SPY	30.6	18.0	18.4
CIB1	0.9	1.2	5.4	c88293_g4	FUL	56.7	29.1	162.4	c90289_g1	SVP	12.9	28.7	0.5
CIB1	0.4	0.1	65.3	c90323_g1	FUL	7.8	0.0	1.2	c90829_g2	SVP	1.6	35.7	1.7
CIB1	8.4	19.8	5.4	c92912_g1	FY	69.0	37.9	37.4	c91377_g1	SVP	52.4	57.9	29.5
CIB1	15.3	10.6	36.7	c95783_g1	FY	24.4	13.3	15.2	c95674_g3	SWN	22.6	30.4	30.1
CIB1	16.0	14.6	82.9	c99108_g3	GI	72.0	27.3	34.6	c75317_g1	TaVRN1	11.8	7.1	0.3
CKA1	35.7	26.7	35.3	c91613_g4	GRF3	312.1	281.0	285.4	c94181_g3	TEM2	17.8	21.6	0.6
CKA1	12.2	8.1	1.3	c93155_g2	GRF3	1.7	0.7	29.6	c94930_g3	TOC1	68.6	1.3	2.4
CKA1	54.2	61.9	42.3	c87227_g1	HAP3B3	12.1	24.1	8.7	c52913_g1	TOE1	0.9	3.4	0.1
CKA1	29.3	11.6	16.9	c98146_g5	HAP3B3	33.1	106.5	30.0	c85378_g3	TOE1	0.6	0.3	2.9
CKA1	50.1	34.2	33.4	c95551_g2	HAP5C	71.7	31.6	83.9	c87192_g2	TOE1	8.1	1.5	4.8
CKA2	22.7	28.4	28.5	c59813_g1	HvFT5	0.1	1.5	5.8	c87192_g5	TOE1	14.1	7.5	3.2
CKA2	113.3	14.6	14.9	c98104_g1	LD	14.8	6.7	5.1	c87982_g1	TOE1	6.2	0.3	1.3
CKA2	156.2	107.6	120.1	c98104_g2	LD	15.2	8.4	7.8	c91054_g4	TOE1	1.1	32.6	0.5
CKA2	54.0	31.0	34.9	c96427_g2	LFY	44.4	1.8	0.2	c96694_g1	TOE1	12.9	1.1	1.2
CKA3	54.4	33.7	29.0	c90643_g1	LHP1	50.7	30.5	27.9	c96979_g5	TOE1	23.5	3.0	0.4
CKA3	4.2	1.2	38.4	c58074_g2	LHY	0.6	3.6	0.0	c98453_g2	TOE1	56.1	16.7	5.8
CKA3	15.3	15.5	9.7	c92088_g3	LHY	63.8	62.3	69.6	c91085_g1	TOE3	11.1	3.9	4.0
CKA3	74.7	49.3	47.2	c99092_g1	LHY	76.6	223.5	137.8	c96979_g6	TOE3	28.6	13.3	8.3
CKA3	59.8	45.3	94.5	c88534_g2	LKP2	19.3	8.4	13.0	c91926_g8	TSF3	8.7	0.7	0.2
CKA3	29.6	14.5	2.5	c96650_g1	LUX	10.2	12.7	8.5	c92401_g3	VEL1	1.5	13.4	4.9
CKA4	80.5	78.9	81.1	c95303_g7	MAF2	8.7	7.5	17.7	c92401_g6	VEL1	4.3	17.7	6.6
CKA4	6.8	13.2	9.0	c93556_g1	OsEhd1	59.0	28.0	21.8	c98995_g1	VRN1	30.7	10.5	23.7
CLF	13.8	9.9	4.4	c97066_g3	OsLFL1	11.4	8.2	6.4	c93433_g2	VRN5	7.5	11.0	11.6
COP1	14.4	28.7	4.4	c82061_g1	OsSPY	63.1	35.8	50.6	c82411_g2	ZmID1	16.0	24.9	11.8
COP1	13.9	26.0	3.9	c87872_g1	PAF2	162.6	112.9	155.3	c92595_g6	ZmID1	109.4	40.8	33.3
COP1	9.8	22.5	3.9	c95421_g1	PHYC	6.7	8.8	9.7	c93923_g1	ZmID1	35.9	15.5	16.1
CRY1	39.2	89.8	43.8	c84580_g2	PIE1	26.1	20.5	25.1	c94665_g2	ZmID1	16.1	21.8	4.8
CRY1	20.1	38.3	26.0	c89213_g2	PIE1	43.9	24.3	25.1	c96507_g4	ZmID1	23.6	36.2	18.2
CRY1	34.4	76.5	45.6	c89266_g3	PIE1	30.0	19.2	15.1	c88070_g1	ZTL	22.1	44.2	58.1
CRY2	4.2	13.1	4.7	c92275_g1	PIE1	17.5	8.1	10.6					

### DE analyses of transgenic blueberry overexpressing VcFT

124,560 gene contigs (69.3% of the 179,853 refTrinity genes) and 187,779 transcript contigs (73.8% of the 254,396 refTrinity genes) were present in young leaves of ‘*VcFT*-Aurora’ plants. In addition, 1,130 transcript contigs (92.4% of the 1,223) of blueberry flowering-related genes were identified. The absence of some gene/transcript contigs is attributed to either tissue-specific or genotype-dependent expression.

**Fig 2:**
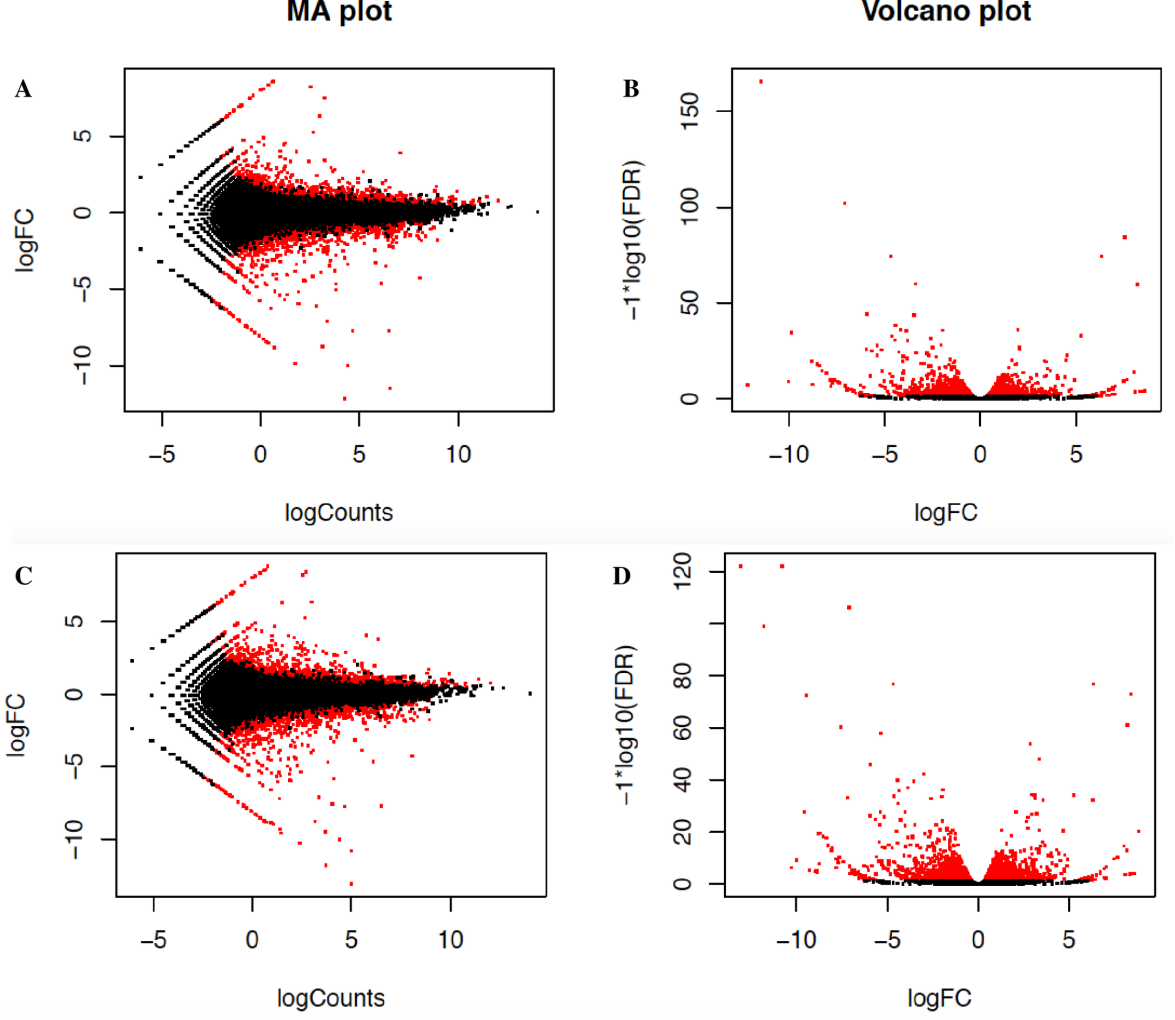
**Comparison of transcript expression profiles between ‘VcFT-Aurora’ and ‘Aurora’ to identify differentially expressed genes (a, b) and isoforms (c, d) in leaf tissues. a, c,** *MA* plot for differential expression analysis generated by EdgeR: for each gene or isoform, the log_2_(fold change) (log_2_(Aurora/VcFT-Aurora)) between the two samples is plotted (*A*, *y* axis) against the gene’s log_2_(average expression) in the two samples (*M*, *x* axis). **b, d,** Volcano plot reporting false discovery rate (−logFDR, *y* axis) as a function of log_2_(fold change) between the samples (logFC, *x* axis). Genes or isoforms that are identified as significantly differentially expressed at most 0.1% FDR are colored in red.

**Fig 3:**
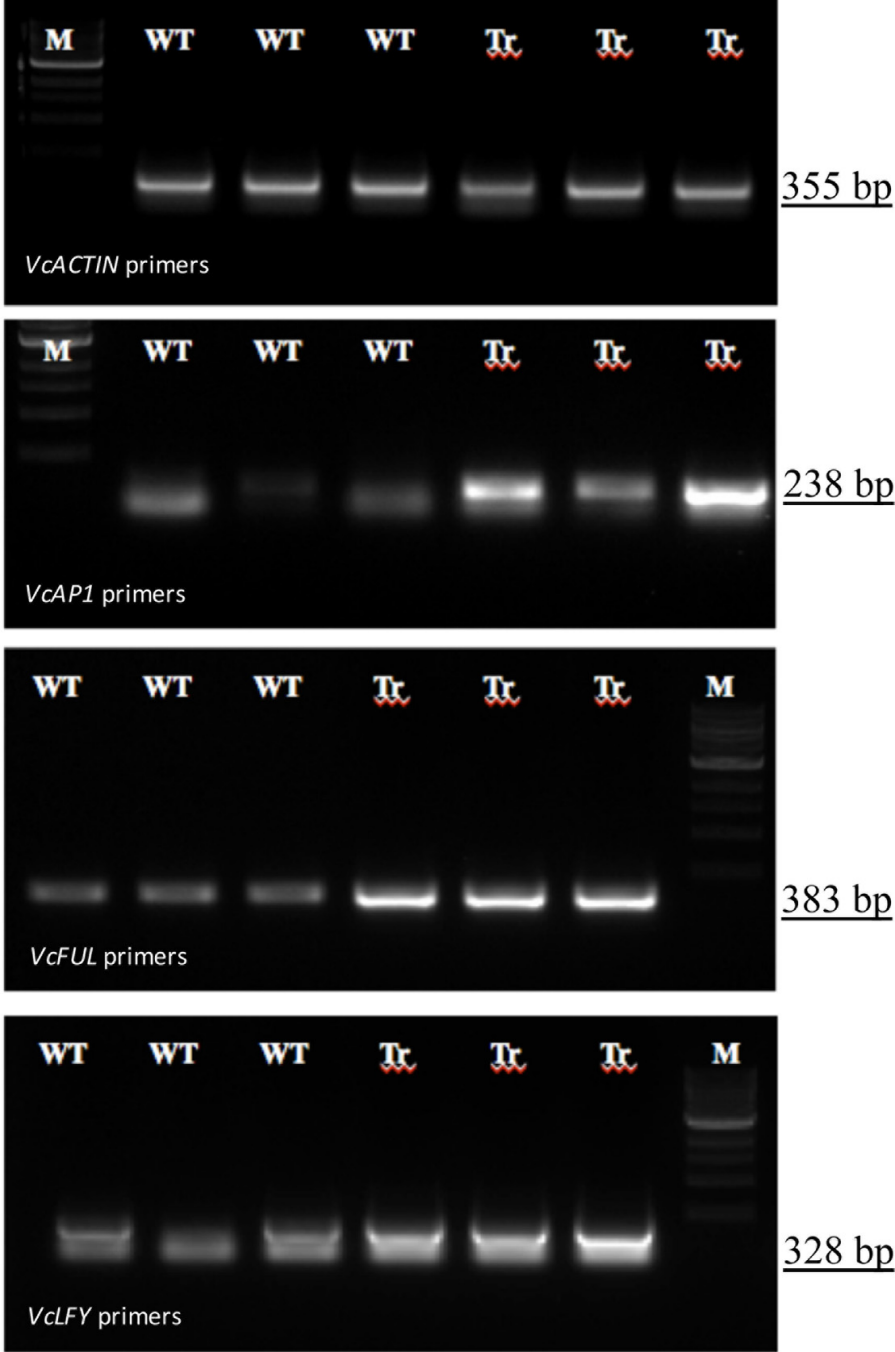
RT-PCR analysis of differentially expressed transcripts in leaf tissues of non-transgenic ‘Aurora’ (WT) and transgenic ‘VcFT-Aurora’ (Tr). *VcACTIN* is the internal control. M: 1 Kb ladder.

Differential expression analyses of transcriptome of ‘VcFT-Aurora’ indicated that over-expressed *VcFT* had a broad impact on global gene expression. We found expression levels of 3,023 Trinity gene contigs (2.4% of 124,560) were significantly altered in ‘VcFT-Aurora’ plants (p-value<0.001 and FDR<0.05), including 1,498 up-regulated and 1,525 down-regulated gene contigs (Fig 2). At the transcript level, 4,844 Trinity transcripts (2.6% of 187,779) were significantly altered, of which 2,270 were up-regulated and 2,574 were down-regulated (Fig 2). Sixty-one transcripts of 40 gene contigs showed high similarities to 33 known flowering-related genes, of which 17 were down-regulated and 16 were up-regulated (Table 3). All down-regulated flowering-related genes were the upstream genes of *VcFT*. The 35S promoter drove high expression of the *VcFT*, which was associated with up-regulated *TWIN SISTER of FT* (*TSF*)-like (*VcTSF*) as well as the downstream genes *VcAP1*, *VcFUL*, *SEPALLATA*-like (*VcSPL*), *VcLFY*, and *VcSOC1*. The up-regulation of the selected *VcAP1*, *VcLFY*, and *VcFUL* was confirmed in our RT-PCR analysis (Fig 3). Interestingly, nine *VcFT* upstream genes were also up-regulated (Table 3).

**Table 3:** Differential expression of blueberry flowering pathway genes in ‘VcFT-Aurora’ leaves and their putative pathways.

Putative Pathway	Gene	Log_2_(fold change) = Log_2_(VcFT-Aurora/Aurora)	FDR
Photoperiod & Temperature	VcPRR5_g1	1.18	1.15E-02
	VcPRR7_g1	0.58	2.67E-03
	VcPRR9_g1	-1.82	1.62E-02
	VcLHY_g1	0.89	1.75E-08
	VcCHE_g1	0.85	3.00E-03
	VcCHE_g2	-0.74	1.96E-03
	VcCHE_g3	-0.95	5.62E-04
	VcCOL5_g1	-1.14	6.26E-11
Hormone	VcABF4_g1	0.70	4.29E-03
	VcCKA3_g1	3.03	2.64E-02
	VcCKA3_g2	1.48	2.77E-06
	VcCKA2_g1	-0.50	9.16E-03
	VcAGL14_g1	-1.30	2.39E-02
	VcAGL14_g2	-2.94	1.31E-03
Age	VcFLD_g1	1.01	2.66E-02
	VcTOE3_g1	0.76	3.51E-03
	VcAGL19_g1	0.61	8.61E-03
	VcFLK_g1	-1.62	5.45E-02
	VcFCA_g1	-0.54	5.61E-03
	VcFCA_g2	-0.57	9.11E-03
	VcTOE1_g1	-1.17	1.15E-02
	VcHAP5C3_g1	-0.58	1.56E-02
	VcSPL_g1	1.55	1.65E-09
	VcSPL_g2	1.01	6.39E-03
	VcSPL_g3	0.85	3.31E-03
	VcSPL_g4	0.45	3.72E-02
	VcSPL_g5	-1.11	6.11E-05
	VcCIB1_g1	0.77	1.21E-04
Vernalization	VcFRI_g1	0.65	2.53E-02
	VcVRN1_g1	2.02	3.43E-03
	VcVRN1_g2	0.79	2.64E-03
	VcSUF4_g1_i1*	-0.50	1.20E-02
	VcPAF2_g1	-0.44	3.72E-02
	VcHUA2_g1	-0.47	3.20E-02
	VcARP6_g1	0.63	4.50E-02
	VcARP6_g2	-0.65	1.70E-03
	VcMAF2_g1/VcMAF5_g1	-2.07	4.53E-04
Conserved Flowering	VcFT_g1	11.46	1.94E-166
	VcFUL_g1	4.73	6.87E-14
	VcSOC1_g1	1.35	1.03E-10
	VcTSF3_g1	3.54	1.32E-09
	VcAP1_g1	5.77	3.52E-02
	VcAP1_g2	3.87	3.59E-36
	VcAP1_g3	3.79	4.30E-31
	VcAP1_g4	3.45	2.96E-08
	VcLFY_g1	2.26	7.30E-07
	VcLFY_g2	1.39	3.47E-07

* Isoform was differentially expressed, but not at gene level.

In ‘VcFT-Aurora’, *VcCOL5* is a major down-regulated flowering pathway gene associated with five other DE genes in the photoperiod-regulated flowering pathway (Table 3). *VcMAF2* and *VcMAF5* or *VcVRN1* may function as integrators of the vernalization pathway, in which six other genes [*i*.*e*., *VcFRI*, *VcHUA2*, *SUPPRESSOR OF FRIGIDA4*-like (*VcSUF4*), *20S PROTEASOME SUBUNIT PAF2*-like (*VcPAF2*), and *ACTIN-RELATED PROTEIN 6* (*ARP6*)-like gene (*VcARP6*)] were involved (Table 3). No *VcCO1* or *VcFLC* were found in blueberry. These results suggest *VcCOL5* and *VcMAF2*/*VcMAF5* or *VcVRN1* in blueberry may be the major integrator(s) in the photoperiod and vernalization pathways, respectively.

Our comparative analyses of flowering pathway genes of blueberry, in addition to the profile of DE flowering-related genes in *VcFT*-over-expressing ‘Aurora’ plants (Table 3), suggest that the *VcFT* down-stream genes (*e*.*g*., *VcSOC1*, *VcFUL*, *VcLFY*, and *VcAP1*) are functionally conserved.

## Discussion

The genetic nature of many fruit crops, *e*.*g*., polyploidy, high heterozygosity, long juvenility, and clonal propagation, makes traditional breeding a lengthy and expensive process. Although a few genetic markers have been developed to facilitate the selection process [9] blueberry breeding is mainly guided by breeders’ experiences. Manipulation of flowering pathway genes is anticipated to change the regime of blueberry flowering and to increase fruit production [4, 5]. To this end, we here have used comparative genomics and biotechnology tools to investigate flowering pathway genes and flowering mechanisms of blueberry.

### Transcriptome reference of blueberry

In a previous study of anthocyanin biosynthesis in blueberry, over 64 million RNA-sequencing reads were generated from blueberry skin and pulp, 34,464 unigenes were obtained through *de novo* assembly [8]. The first whole genome sequence of blueberry with 15,129 scaffolds for 358 Mb was reported on a diploid *V*. *corymbosum* accession ‘W8520’ [2, 7]. Using this draft genome reference and RNA-seq data, great efforts have been made to identify genes involved in blueberry ripening and over 400 biosynthetic pathways (*e*.*g*., ethylene and anthocyanin) [7]. Currently, there is an online blueberry genomics database (BBDG) that contains a collection of EST sequences (http://bioinformatics.towson.edu/BBGD/)), and some published RNA-seq data are available in the GenBank [8]. To date, no blueberry genome reference has been made available to the public.

We demonstrated that the Trinity platform [24] is amenable to use and is very efficient in generating transcriptome references for tetraploid blueberry. With 72.7 MR (8–10 MR/sample), we believe our transcriptome reference (refTrinity) covers the majority of transcripts in leaves, flowers, and flower buds. Although this transcriptome reference does not include root- and fruit-specific transcripts, it provides a good coverage of the majority of blueberry flowering pathway genes for studying gene expression in blueberry plants.

### Conserved flowering pathway genes in blueberry

In this study, it is not surprising that the orthologues of the majority of the flowering pathway genes found in *Arabidopsis*, rice, and cereals crops were identified in blueberry, although some of these orthologues may have distinctive functions to meet the specific needs of blueberry floral transitions or flowering. Meanwhile, there could be other genes (*e*.*g*., *VcCO1* and *VcFLC*) that might have eluded our comparative genomic analysis due to their high specificity or uniqueness to blueberry.

### Flowering gene network of blueberry

**Fig 4:**
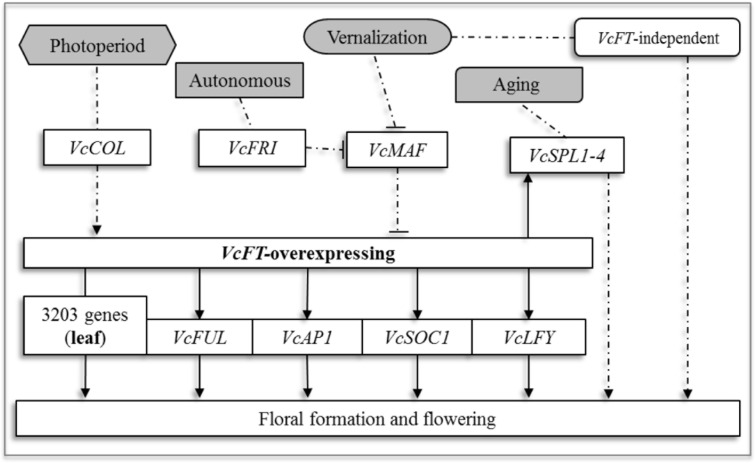
Flowering pathways in blueberry. Arrows: promotion. T-signs: inhibition. Solid lines: results of this study. Dashed lines: proposed correlation.

Based on profiles of DE flowering-related genes in ‘VcFT-Aurora’, we propose a flowering gene network in blueberry (Fig 4). In this network, blueberry has a universal *FT*-mediated flowering pathway, where the photoperiod pathway (*e*.*g*., *VcCOL2*, and *VcCOL5*), and the vernalization and autonomous pathways (*e*.*g*., *VcFRI*, *VcMAF2*, *VcMAF5*, or *VcVRN1*) function through *VcFT* and its downstream integrators *VcSOC1* and *VcLFY*, although no orthologues of *CO1* and *FLC* were identified. In this universal pathway, over-expressing *VcFT* promotes floral bud formation and flowering. *VcFT* also appears to promote expression of several *SPL* (*SQUAMOSA PROMOTER BINDING LIKE*) homologs in the aging pathway. However, *VcFT* over-expression is not sufficient to completely reverse the influence of environmental stimuli for normal plant flowering; for example, ‘VcFT-Aurora’ plants did not flower normally when grown under non-chilling conditions. It is possible that there are additional genes/pathways independent of *VcFT* expression that regulate vernalization-mediated blueberry flowering.

In conclusion, we developed a transcriptome reference of highbush blueberry using RNA sequencing data and Trinity. By searching this transcriptome using known flowering pathway gene sequences of *Arabidopsis*, rice and cereal, we developed a reference of blueberry flowering-related genes. Comparative genomics of flowering pathway genes in blueberry and herbaceous plants revealed a conserved *VcFT*-mediated flowering through its down-stream *VcSOC1*, *VcAP1*, *VcLFY*, and *VcFUL* genes. Interestingly, neither *VcCO1* nor *VcFLC* are present in our transcriptome reference. The profile of DE flowering pathway genes in leaf tissues of *VcFT*-overexpressing plants suggests *VcCOL5* and *VcMAF2*/*VcMAF5* may be the major integrator(s) in the photoperiod and vernalization pathway, respectively.

## Supporting Information

S1 Table Primer sequences for RT-PCR analysis. (DOCX)

S2 Table Identities of flowering pathway genes in herbaceous plants (derived from Higgins et al., 2010). (DOCX)

S3 Table Analysis of refTrinity assembly quality and completeness by alignment with existing unigene sequences. Data from blastn of BLASTplus with e-value threshold of 1e-5 using indicated query and refTrinity as database.
(DOCX)

S4 Table Identities of flowering pathway genes/transcripts of blueberry. (DOCX)
