# Supplementary material for: Transcript Profile of Flowering Regulatory Genes in VcFT-Overexpressing Blueberry Plants
Source: PLoS One. 2016 Jun 7;11(6):e0156993. doi: 10.1371/journal.pone.0156993 (PMC4896415; doi:10.1371/journal.pone.0156993)
Supplement: S1 Table — (DOCX) [file pone.0156993.s001.docx]

**S1 Table.** **Primer sequences for RT PCR analysis.**

| Primer name | Primer (5' to 3' end) | Gene ID |
| --- | --- | --- |
| TA1F | GAGAGATTCAGATGCCCAGAAG | *VcACTIN* |
| TA1R | GGACAATGGATGGACCAGATT | *VcACTIN* |
|  |  |  |
| BBAP1F | GCCGAGGTGGCGCTTAT | *VcAP1* |
| BBAP3R | AGGTATCGAGGTCTTCTCCCATATAG | *VcAP1* |
|  |  |  |
| VcFULF | AGTTCATCCACCTTGGTCTTAC | *VcFUL* |
| VcFULR | GTCCACTTCGCGTACAATCT | *VcFUL* |
|  |  |  |
| VcLFYF | CTTGATCCAGGTCCAGAGTATTG | *VcLFY* |
| VcLFYR | GGGAACATACCAAACCGAGAG | *VcLFY* |
