# Supplementary material for: Transcript Profile of Flowering Regulatory Genes in VcFT-Overexpressing Blueberry Plants
Source: PLoS One. 2016 Jun 7;11(6):e0156993. doi: 10.1371/journal.pone.0156993 (PMC4896415; doi:10.1371/journal.pone.0156993)
Supplement: S2 Table — (DOCX) [file pone.0156993.s002.docx]

**S2 Table. Identities of flowering pathway genes in herbaceous plants (derived from Higgins et al., 2010).**

| Arabidopsis | | | | | Rice | | | | | | Cereals - Wheat /Barley/Maize | |
| --- | --- | --- | --- | --- | --- | --- | --- | --- | --- | --- | --- | --- |
| Gene | AGI code TAIR 9 | | Uniprot | | Rice MSU release 6.1 | | | Uniprot | Name | Synonym | Accession | Protein / gene name |
| *PHYA* | AT1G09570.1 | |  | | LOC_Os03g51030.1 | | |  | OsPHYA |  | ABB13321 | HvPHYA |
| *PHYB* | AT2G18790.1 | |  | | LOC_Os03g19590.1 | | |  | OsPHYB |  |  |  |
| *PHYC* | AT5G35840.1 | |  | | LOC_Os03g54084.1 | | |  | OsPHYC |  |  |  |
| *PHYD* | AT4G16250.1 | |  | |  | | |  |  |  |  |  |
| *PHYE* | AT4G18130.1 | |  | |  | | |  |  |  |  |  |
| *CRY1* | AT4G08920.1 | |  | | LOC_Os02g36380.1 | | |  | OsCRY1a |  |  |  |
|  |  | |  | | LOC_Os04g37920.1 | | |  | OsCRY1b |  |  |  |
| *CRY2* | AT1G04400.2 | |  | | LOC_Os02g41550.1 | | |  | OsCRY2 |  |  |  |
| *CKA1* | AT5G67380.1 | |  | | LOC_Os03g55389.1 | | |  | Hd6 |  |  |  |
| *CKA2* | AT3G50000.1 | |  | | LOC_Os07g02350.1 | | |  |  |  |  |  |
| *CKA3* | AT2G23080.1 | |  | | LOC_Os03g55490.1 | | |  |  |  |  |  |
| *CKA4* | AT2G23070.1 | |  | | LOC_Os03g10940.1 | | |  |  |  |  |  |
| *ZTL* | AT5G57360.2 | |  | | LOC_Os06g47890.1 | | |  | OsZTLa |  |  |  |
| *LKP2* | AT2G18915.2 | |  | | LOC_Os02g05700.1 | | |  | OsZTLb |  |  |  |
| *FKF1* | AT1G68050.1 | |  | | LOC_Os11g34460.1 | | |  | OsFKF1 |  |  |  |
| *ELF3* | AT2G25930.1 | |  | | LOC_Os01g38530.1 | | |  | OsELF3 |  | ABL11477 | TaELF3 |
|  | AT3G21320.1 | |  | | LOC_Os06g05060.1 | | |  | OsELF3 |  |  |  |
| *ELF4* | AT2G40080.1 | | \|  \|  \|  \|  \| \| --- \| --- \| --- \| --- \| \|  \| 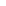 \|  \|  \| \|  \|  \|  \|  \| \|  \|  \| \| 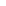 \| | | LOC_Os11g40610.1 | | |  |  |  |  |  |
|  | AT1G17455.1 | |  | | LOC_Os03g29680.1 | | |  |  |  |  |  |
|  | AT1G72630.1 | |  | | LOC_Os08g27860.1 | | |  |  |  |  |  |
|  | AT2G06255.1 | |  | | LOC_Os08g27870.1 | | |  |  |  |  |  |
|  | AT2G29950.1 | |  | |  | | |  |  |  |  |  |
| *GI* | AT1G22770.1 | |  | | LOC_Os01g08700.1 | | |  | OsGI |  | AAW66946 | Gigantea-like |
| *SPY* | AT3G11540.1 | |  | | LOC_Os08g44510.1 | | |  | OsSPY |  |  |  |
| *LUX* | AT3G46640.1 | |  | | LOC_Os01g74020.1 | | |  | OsLUX |  |  |  |
| *LHY* | AT1G01060.1 | |  | | LOC_Os08g06110.2 | | |  | OsCCA1 |  |  |  |
| *CCA1* | AT2G46830.1 | |  | |  | | |  |  |  |  |  |
| *CHE* | AT5G08330.1 | |  | |  | | |  |  |  |  |  |
| *TOC1* | AT5G61380.1 | |  | | LOC_Os02g40510.1 | | |  | OsTOC1 |  |  |  |
| *PRR3* | AT5G60100.1 | |  | | LOC_Os07g49460.2 | | |  | OsPRR37 |  | AAY42109 | Ppd-H1 |
| *PRR 7* | AT5G02810.1 | |  | | LOC_Os03g17570.1 | | |  | OsPRR73 |  | AK250331 |  |
| *PRR5* | AT5G24470.1 | |  | | LOC_Os11g05930.1 | | |  | OsPRR59 |  |  |  |
| *PRR9* | AT2G46790.1 | |  | | LOC_Os09g36220.1 | | |  | OsPRR95 |  |  |  |
| *COP1* | AT2G32950.1 | |  | | LOC_Os02g53140.1 | | |  | OsCOP1 |  |  |  |
| *CDF1* | AT5G62430.1 | |  | | LOC_Os01g15900.1 | | |  |  |  | AK252924 | HvCDF |
| *CDF2* | AT5G39660.1 | |  | | LOC_Os01g17000.1 | | |  |  |  |  | TaDOF5 |
| *CDF3* | AT3G47500.1 | |  | | LOC_Os07g48570.1 | | |  |  |  |  | TaDOF16 |
|  | AT1G69570.1 | |  | | LOC_Os03g07360.1 | | |  |  |  |  | TaDOF17 |
|  | AT1G26790.1 | |  | | LOC_Os10g26620.1 | | |  |  |  |  | TaDOF18 |
|  | AT1G29160.1 | |  | |  | | |  |  |  |  | TaDOF19 |
|  | AT2G34140.1 | |  | |  | | |  |  |  |  | TaDOF20 |
|  |  | |  | |  | | |  |  |  |  | TaDOF26 |
|  |  | |  | |  | | |  |  |  |  | TaDOF29 |
|  |  | |  | |  | | |  |  |  |  | TaDOF31 |
| *PFT1* | AT1G25540.1 | |  | | LOC_Os09g13610.1 | | |  | OsPFT1 |  |  |  |
| *CONSTANS* | AT5G15840.1 | |  | | LOC_Os06g16370.1 | | | Q30DN4_ORYSI | Hd1 | OsA | AAM74063 | HvCO1 |
| *AtCOL1* | AT5G15850.1 | |  | |  | | |  |  |  | AAM74065 | HvCO2 |
| *AtCOL2* | AT3G02380.1 | |  | |  | | |  |  |  | AAT42130 | TaCO2 |
| *AtCOL3* | AT2G24790.1 | |  | | LOC_Os09g06464.1 | | |  | OsCO3 | OsB | AAM74068 | HvCO3 |
| *AtCOL4* | AT5G24930.1 | |  | | LOC_Os04g42020.1 | | |  |  | OsC | AAM74070 | HvCO4 |
| *ATCOL5* | AT5G57660.1 | |  | | LOC_Os02g39710.1 | | |  |  | OsD | AAL99264 | HvCO5 |
|  |  | |  | | LOC_Os06g44450.1 | | |  |  | OsE | AAL99268 | HvCO6 |
|  |  | |  | | LOC_Os02g08150.1 | | |  |  | OsF | AAL99269 | HvCO7 |
|  |  | |  | | LOC_Os08g15050.1 | | |  |  | OsG | AAL99270 | HvCO8 |
| *SPA1* | AT2G46340.1 | |  | | LOC_Os01g52640.3 | | |  | OsSPA1 |  |  |  |
| *SPA2* | AT4G11110.1 | |  | | LOC_Os05g49590.2 | | |  |  |  |  |  |
| *SPA3* | AT3G15354.1 | |  | |  | | |  |  |  |  |  |
| *SPA4* | AT1G53090.1 | |  | |  | | |  |  |  |  |  |
| *TOE1* | AT2G28550.1 | |  | | LOC_Os05g03040.1 | | |  |  |  | AAC05206 | ZmIDS1 |
| *TOE2* | AT5G60120.1 | |  | | LOC_Os07g13170.1 | | |  |  |  | AAU94922 | TaQ |
| *SMZ* | AT3G54990.1 | |  | | LOC_Os05g03040.1 | | |  |  |  | ABR19871 | ZmRAP2.7 Vgt1 |
| *SNZ* | AT2G39250.1 | |  | | LOC_Os04g55560.2 | | |  |  |  |  |  |
| *TOE3* | AT5G67180.1 | |  | | LOC_Os06g43220.1 | | | B8B0I8_ORYSI |  |  |  |  |
| *AP2* | AT4G36920.1 | |  | |  | | |  |  |  |  |  |
| *TEM1* | AT1G25560.1 | |  | | LOC_Os05g47650.1 | | |  | OsRAV8 |  |  |  |
| *TEM2* | AT1G68840.1 | |  | | LOC_Os01g04800.1 | | |  | OsRAV9 |  |  |  |
| *RAV1* | AT1G13260.1 | |  | | LOC_Os01g49830.1 | | |  | OsRAV11 |  |  |  |
| *RAV1-like* | AT3G25730.1 | |  | | LOC_Os01g04750.1 | | |  | OsRAV12 |  |  |  |
|  | AT1G50680.1 | |  | |  | | |  |  |  |  |  |
|  | AT1G51120.1 | |  | |  | | |  |  |  |  |  |
| *ELF6* | AT5G04240.1 | |  | | LOC_Os03g05680/90 | | | A3AE68_ORYSJ | OsELF6 |  |  |  |
| *REF6* | AT3G48430.1 | |  | | LOC_Os01g67970.1 | | |  | OsREF6 |  |  |  |
|  |  | |  | | LOC_Os10g32600.1 | | |  | OsEhd1 |  |  |  |
|  |  | |  | | LOC_Os10g28330.1 | | |  | OsID1 | RID1 Ehd2 | AAC18941 | ZmID1 |
|  |  | |  | | LOC_Os01g09850.1 | | |  | OsIDD2 |  | C5X1F3 | SbID1 |
|  |  | |  | | LOC_Os01g70870.1 | | |  | OsIDD9 |  |  |  |
| *CIB1* | AT4G34530.1 | |  | |  | | |  |  |  |  |  |
| *FT* | AT1G65480.1 | |  | | LOC_Os01g11940.1 | | |  | OsFTL1 |  | ABB99414 | HvFT2 |
| *TSF* | AT4G20370.1 | |  | | LOC_Os06g06320.1 | | |  | Hd3a | OsFTL2 | AAZ38709 | HvFT1 |
|  | AT1G18100.1 | |  | | LOC_Os06g06300.1 | | |  | OsFTL3 | OsRFT1 |  |  |
|  | AT2G27550.1 | |  | | LOC_Os09g33850.1 | | |  | OsFTL4 |  |  |  |
| *TFL1* | AT5G03840.1 | |  | | LOC_Os02g39064.1 | | |  | OsFTL5 |  |  |  |
| *BFT* | AT5G62040.1 | |  | | LOC_Os04g41130.1 | | |  | OsFTL6 |  |  |  |
|  |  | |  | | LOC_Os12g13030.1 | | |  | OsFTL7 |  |  |  |
|  |  | |  | |  | | |  |  |  |  |  |
|  |  | |  | |  | | |  |  |  |  |  |
|  |  | |  | | LOC_Os01g10590.1 | | |  | OsFTL8 |  |  |  |
|  |  | |  | | LOC_Os01g54490.1 | | |  | OsFTL9 |  |  |  |
|  |  | |  | |  | | |  |  |  | ABD75336 | HvFT3 |
|  |  | |  | |  | | |  |  |  | ABM26903 | HvFT5 |
|  |  | |  | | LOC_Os05g44180.1 | | |  | OsFTL10 |  |  |  |
|  |  | |  | | LOC_Os11g18870.1 | | |  | OsFTL11 |  |  |  |
|  |  | |  | | LOC_Os06g35940.1 | | |  | OsFTL12 |  | ABD75337 | HvFT4 |
|  |  | |  | | LOC_Os02g13830.1 | | |  | OsFTL13 |  |  |  |
|  |  | |  | | LOC_Os06g30370.1 | | |  | OsMFT1 |  |  |  |
|  |  | |  | |  | | |  |  |  |  |  |
|  |  | |  | | LOC_Os01g02120 .1 | | |  | OsMFT2 |  |  |  |
|  |  | |  | | LOC_Os11g05470.1 | | |  | OsTFL1 | OsRCN1 |  |  |
|  |  | |  | | LOC_Os02g32950.1 | | |  | OsRCN2 |  |  |  |
|  |  | |  | | LOC_Os12g05590.1 | | |  | OsRCN3 |  |  |  |
|  |  | |  | | LOC_Os04g33570.1 | | |  | OsRCN4 |  |  |  |
| *HAP3A* | AT2G38880.1 | |  | | LOC_Os07g41580.1 | | |  |  |  |  |  |
| *HAP3B* | AT5G47640.1 | |  | | LOC_Os03g29970.1 | | |  |  |  |  |  |
|  | AT2G13570.1 | |  | | LOC_Os05g38820.1 | | |  |  |  |  |  |
|  | AT4G14540.1 | |  | | LOC_Os05g49780.1 | | |  |  |  |  |  |
|  | AT2G37060.1 | |  | | LOC_Os01g61810.1 | | |  |  |  |  |  |
|  | AT3G53340.1 | |  | |  | | |  |  |  |  |  |
| *HAP5A* | AT3G48590.1 | |  | | LOC_Os02g07450.1 | | |  |  |  |  |  |
| *HAP5B* | AT1G56170.1 | |  | | LOC_Os06g45640.1 | | |  |  |  |  |  |
| *HAP5C* | AT1G08970.1 | |  | | LOC_Os03g14669.1 | | |  |  |  |  |  |
|  | AT1G54830.1 | |  | | LOC_Os08g38780.1 | | |  |  |  |  |  |
|  | AT5G63470.1 | |  | | LOC_Os09g30310.1 | | |  |  |  |  |  |
| *GRF2* | AT1G78300.1 | |  | | LOC_Os02g36974.1 | | |  | OsGF14e |  |  |  |
| *GRF1* | AT4G09000.1 | |  | | LOC_Os08g33370.1 | | |  | OsGF14c |  |  |  |
| *GRF4* | AT1G35160.1 | |  | | LOC_Os04g38870.1 | | |  | OsGF14b |  |  |  |
| *GRF3* | AT5G38480.1 | |  | | LOC_Os03g50290.1 | | |  | OsGF14f |  |  |  |
| *GRF7* | AT3G02520.1 | |  | | LOC_Os11g34450.1 | | |  | OsGF14d |  |  |  |
| *GRF5* | AT5G16050.1 | |  | | LOC_Os08g37490.1 | | |  | OsGF14a |  |  |  |
| *GRF8* | AT5G65430.1 | |  | |  | | |  |  |  |  |  |
| *GRF6* | AT5G10450.1 | |  | |  | | |  |  |  |  |  |
| *SOC1* | At2g45660.1 | |  | | LOC_Os03g03070/100 | | | MAD50_ORYSJ | OsMADS50 |  | BAF56968 | TaSOC1 |
| *AGL19* | At4g22950.1 | |  | | LOC_Os10g39130.1 | | |  | OsMADS56 |  |  |  |
| *AGL14* | At4g11880.1 | |  | | LOC_Os05g38820.1 | | |  |  |  |  |  |
|  |  | |  | | LOC_Os02g01355.1 | | |  |  |  |  |  |
|  |  | |  | | LOC_Os02g01365.1 | | |  |  |  |  |  |
|  |  | |  | | LOC_Os08g41960.1 | | |  | OsMADS37 |  |  |  |
|  |  | |  | | LOC_Os01g69850.1 | | |  | OsMADS51 |  | ABF57941 | TaAGL41 |
|  |  | |  | |  | | |  |  |  |  |  |
|  |  | |  | |  | | |  |  |  |  |  |
| *ELF9* | AT5G16260.1 | |  | | LOC_Os04g41910.1 | | | | OsELF9 |  |  |  |
|  |  | |  | | LOC_Os01g51610.1 | |  | | OsLFL1 |  |  |  |
| *LFY* | AT5G61850.1 | |  | | LOC_Os04g51000.1 | |  | | OsLFY |  |  |  |
| *AP1* | At1g69120.1 | |  | | LOC_Os03g54160.1 | |  | | OsMADS14 |  | AAW73221 | TaVRN1 |
| *AGL79* | At3g30260.1 | |  | | LOC_Os07g01820.1 | |  | | OsMADS15 |  | ABF57930 | TaAGL29 |
| *CAL1* | At1g26310.1 | |  | | LOC_Os07g41370.1 | |  | | OsMADS18 |  | ABF57915 | TaAGL10 |
| *FUL* | At5g60910.1 | |  | | LOC_Os12g31748.1 | |  | | OsMADS20 |  |  |  |
|  |  | |  | |  | |  | |  |  |  |  |
|  |  | |  | | LOC_Os10g41100.1 | |  | | OsP |  | AAL99271 | HvCO9 |
|  |  | |  | | LOC_Os07g15770.1 | |  | | OsGhd7 |  | AAS58482 | TmCCT2 |
|  |  | |  | |  | |  | |  |  | AAS60249 | HvZCCTa |
|  |  | |  | |  | |  | |  |  | AAS60250 | HvZCCTb |
|  |  | |  | |  | |  | |  |  | AAS60238 | TmZCCT1 |
|  |  | |  | |  | |  | |  |  | AAT96853 | HvZCCTc |
| *FD* | AT4G35900.1 | | FD_ARATH | | LOC_Os01g59760.1 | |  | |  |  | ABZ91908 | TaFDL2 |
| *FDP* | AT2G17770.1 | | Q7PCC6_ARATH | | LOC_Os05g41070.1 | |  | |  |  |  |  |
| *AREB3* | AT3G56850.1 | |  | | LOC_Os07g48660.1 | |  | |  |  |  |  |
|  | AT2G41070.1 | |  | | LOC_Os03g20650.1 | |  | |  |  | ABZ91909 | TaFDL3 |
|  | AT5G44080.1 | |  | | LOC_Os05g36160.1 | |  | |  |  | ABZ91910 | TaFDL6 |
| *GBF4* | AT1G03970 .1 | |  | | LOC_Os01g64730.1 | |  | |  |  |  |  |
| *AtbZIP67* | AT3G44460.1 | |  | | LOC_Os01g64000.1 | |  | |  |  |  |  |
| *ABI5* | AT2G36270 .1 | |  | | LOC_Os09g28310.1 | |  | |  |  |  |  |
| *ABF2* | AT1G45249.1 | |  | | LOC_Os08g36790.1 | |  | |  |  |  |  |
| *ABF1* | AT1G49720.1 | |  | | LOC_Os06g10880.1 | |  | |  |  |  |  |
| *ABF4* | AT3G19290.1 | |  | | LOC_Os02g52780.1 | |  | |  |  |  |  |
| *AtbZIP15* | AT5G42910.1 | |  | | LOC_Os09g36910.1 | |  | |  |  |  |  |
| *ABF3* | AT4G34000.1 | |  | | LOC_Os08g43600.1 | |  | |  |  | ABK91939 | ZmDFL1 |
|  |  | |  | | LOC_Os02g58670.1 | |  | |  |  | ABZ91911 | TaFDL13 |
|  |  | |  | | LOC_Os06g50600.1 | |  | |  |  | ABZ91912 | TaFDL15 |
|  |  | |  | | LOC_Os06g50830.1 | |  | |  |  |  |  |
|  |  | |  | | LOC_Os06g50480.1 | |  | |  |  |  |  |
| *SPL* | AT2G33810.1 | |  | |  | |  | |  |  |  |  |
| *FLC* | AT5G10140.1 | |  | | LOC_Os04g52410.1 | |  | | OsMADS31 |  |  |  |
| *FLM* | AT1G77080.2 | |  | |  | |  | |  |  |  |  |
| *MAF2* | AT5G65050.1 | |  | |  | |  | |  |  |  |  |
| *MAF3* | AT5G65060.1 | |  | |  | |  | |  |  |  |  |
| *MAF4* | AT5G65070.1 | |  | |  | |  | |  |  |  |  |
| *MAF5* | AT5G65080.1 | |  | |  | |  | |  |  |  |  |
| *AGL18* | AT3G57390.1 | |  | |  | |  | |  |  |  |  |
| *AGL32* | AT5G23260.1 | |  | |  | |  | |  |  |  |  |
| *AGL24* | AT4G24540.1 | |  | | LOC_Os06g11330.1 | |  | | OsMADS55 |  | AAY43789 | TaVRT2 |
| *SVP* | AT2G22540.1 | |  | | LOC_Os02g52340.1 | |  | | OsMADS22 |  | ABB13347 | HvVRT2 |
|  |  | |  | | LOC_Os03g08754.1 | |  | | OsMADS47 |  | A1YJE4 | HvBM10 |
|  |  | |  | |  | |  | |  |  | Q9LEI4 | HvBM1 |
| *FRI* | AT4G00650.1 | | Q52S96_ARATH | | LOC_Os03g63440.1 | |  | | OsFRI |  |  |  |
|  | AT5G27220.1 | |  | | LOC_Os03g09310.1 | |  | |  |  |  |  |
|  |  | |  | | LOC_Os03g39129.1 | |  | |  |  |  |  |
|  |  | |  | | LOC_Os03g39170.1 | |  | |  |  |  |  |
| *SUF4* | AT1G30970.1 | |  | | LOC_Os09g38790.1 | |  | | OsSUF4 |  |  |  |
| *FCA* | AT4G16280.1 | |  | | LOC_Os09g03610.1 | |  | | OsFCA |  | ACI16484 | HvFCA |
| *FY* | AT5G13480.1 | |  | | LOC_Os01g72220.1 | |  | | OsFY |  |  |  |
| *FLD* | AT3G10390.1 | |  | | LOC_Os04g47270.1 | |  | | OsFLD |  |  |  |
| *FPA* | AT2G43410.1 | |  | | LOC_Os09g34070.1 | |  | | OsFPA |  |  |  |
| *FVE* | AT2G19520.1 | |  | | LOC_Os01g51300.2 | |  | | OsFVE |  |  |  |
| *LD* | AT4G02560.1 | |  | | LOC_Os01g70810.1 | |  | | OsLD |  |  |  |
| *FLK* | AT3G04610.1 | |  | | LOC_Os12g40560.2 | |  | | OsFLKa |  |  |  |
|  |  | |  | | LOC_Os03g42900.1 | |  | | OsFLKb |  |  |  |
| *LHP1* | AT5G17690.1 | |  | | LOC_Os10g17770.1 | |  | | OsLHP1 |  |  |  |
| *HUA2* | AT5G23150.1 | |  | | LOC_Os08g01054.1 | |  | |  |  |  |  |
| *PEP* | AT4G26000.1 | |  | | LOC_Os10g41440.1 | |  | | OsPEP |  |  |  |
| *CLF* | AT2G23380.1 | |  | | LOC_Os06g16390.1 | |  | | OsCLF |  |  |  |
| *FIE1* | AT3G20740.1 | |  | | LOC_Os08g04270.1 | |  | | OsFIE1a |  |  |  |
|  |  | |  | | LOC_Os08g04290.1 | |  | | OsFIE1b |  |  |  |
| *MSI1* | AT5G58230.1 | |  | | LOC_Os03g43890.1 | |  | | OsMSI1 |  |  |  |
| *SWN* | AT4G02020.1 | |  | | LOC_Os03g19480.1 | |  | | OsSWN |  |  |  |
| *VRN5* | AT3G24440.1 | |  | | LOC_Os12g34850.1 | |  | |  |  | A0SQ43 | TmVIL1 |
| *VEL1* | AT4G30200.2 | |  | | LOC_Os05g05310.1 | |  | |  |  | A0SQ42 | TmVIL3 |
| *VEL2* | AT2G18880.1 | |  | | LOC_Os08g12430.1 | |  | |  |  |  |  |
| *VEL3* | AT2G18870.1 | |  | |  | |  | |  |  | A0SQ41 | TmVIL2 |
| *VIN3* | AT5G57380.1 | |  | | LOC_Os02g05840.1 | |  | |  |  |  |  |
| *VRN1* | AT3G18990.1 | |  | |  | |  | |  |  |  |  |
| *VRN2* | AT4G16845.1 | |  | | LOC_Os09g13630.1 | |  | |  |  |  |  |
| *EMF2* | AT5G51230.1 | |  | | LOC_Os04g08034.1 | |  | |  |  |  |  |
| *FIE2* | AT2G35670.1 | |  | |  | |  | |  |  |  |  |
|  | AT4G16810.1 | |  | |  | |  | |  |  |  |  |
| *PAF1* | AT5G42790.1 | |  | | LOC_Os02g04100.1 | |  | | OsPAF |  |  |  |
| *PAF2* | AT1G47250.1 | |  | |  | |  | |  |  |  |  |
| *PIE1* | AT3G12810.1 | |  | | LOC_Os02g46450.1 | |  | | OsPIE1 |  |  |  |
| *EFS* | AT1G77300.1 | |  | | LOC_Os02g34850.1 | |  | |  |  |  |  |
| *ARP6* | AT3G33520.1 | |  | | LOC_Os01g16414.1 | |  | | OsARP6 |  |  |  |
| * Source: http://www.modelcrop.org/ | | | | | | |  | |  |  |  |  |
|  | |  | |  | |  |  | |  |  |  |  |
