# Supplementary material for: Transcript Profile of Flowering Regulatory Genes in VcFT-Overexpressing Blueberry Plants
Source: PLoS One. 2016 Jun 7;11(6):e0156993. doi: 10.1371/journal.pone.0156993 (PMC4896415; doi:10.1371/journal.pone.0156993)
Supplement: S3 Table — Data from blastn of BLASTplus with e-value threshold of 1e-5 using indicated query and refTrinity as database. (DOCX) [file pone.0156993.s003.docx]

**S3 Table. Analysis of refTrinity assembly quality and completeness by alignment with existing unigene sequences of highbush blueberries.** Data from blastn of BLASTplus with e-value threshold of 1e-5 using indicated query and refTrinity as database.

| Query number | Identity threshold | Complete length threshold | Mapped | Percent mapped |
| --- | --- | --- | --- | --- |
| 2955 singlets | 95 | 95 | 1545 | 52.28% |
|  |  | 90 | 1669 | 56.48% |
|  |  | 80 | 1776 | 60.10% |
|  |  | 0 | 2385 | 80.71% |
|  | 90 | 95 | 1647 | 55.74% |
|  |  | 90 | 1798 | 60.54% |
|  |  | 80 | 1922 | 65.04 |
|  |  | 0 | 2601 | 88.02 |
|  | 80 | 95 | 1679 | 56.82% |
|  |  | 90 | 1836 | 62.13% |
|  |  | 80 | 1985 | 67.17% |
|  |  | 0 | 2695 | 91.20% |
| 748 contigs | 95 | 95 | 460 | 61.50% |
|  |  | 90 | 494 | 66.04% |
|  |  | 80 | 529 | 70.72% |
|  |  | 0 | 677 | 90.51% |
|  | 90 | 95 | 481 | 64.30% |
|  |  | 90 | 521 | 69.65% |
|  |  | 80 | 556 | 74.33% |
|  |  | 0 | 703 | 93.98% |
|  | 80 | 95 | 483 | 64.57% |
|  |  | 90 | 525 | 70.19% |
|  |  | 80 | 564 | 75.40% |
|  |  | 0 | 710 | 94.92% |
| 3703 unigenes | 95 | 95 | 2005 | 54.15% |
|  |  | 90 | 2163 | 58.41% |
|  |  | 80 | 2305 | 62.25% |
|  |  | 0 | 3062 | 82.69% |
|  | 90 | 95 | 2128 | 57.47% |
|  |  | 90 | 2310 | 62.38% |
|  |  | 80 | 2478 | 66.92% |
|  |  | 0 | 3304 | 89.22% |
|  | 80 | 95 | 2162 | 58.39% |
|  |  | 90 | 2361 | 63.76% |
|  |  | 80 | 2549 | 68.84% |
|  |  | 0 | 3405 | 91.95% |
