# Supplementary material for: Transcript Profile of Flowering Regulatory Genes in VcFT-Overexpressing Blueberry Plants
Source: PLoS One. 2016 Jun 7;11(6):e0156993. doi: 10.1371/journal.pone.0156993 (PMC4896415; doi:10.1371/journal.pone.0156993)
Supplement: S4 Table — (DOCX) [file pone.0156993.s004.docx]

**S4 Table. Identities of flowering pathway genes/transcripts of blueberry.**

| **Query id** | **Subject id** | **% identity** | **e-value** | **bit score** |
| --- | --- | --- | --- | --- |
| AT5G39660.1 | c75935_g1_i1 | 58.43 | 3.00E-39 | 144 |
| AT4G22950.1 | c85724_g1_i1 | 59.34 | 4.00E-44 | 163 |
| AT3G33520.1 | c89508_g1_i1 | 39.79 | 6.00E-42 | 154 |
| AT1G49720.2 | c89508_g3_i1 | 67.37 | 2.00E-29 | 120 |
| AT1G49720.2 | c89508_g3_i2 | 63 | 2.00E-29 | 116 |
| AT2G19520.1 | c89508_g3_i4 | 67.37 | 5.00E-31 | 120 |
| AT1G49720.2 | c89508_g3_i5 | 67.37 | 2.00E-30 | 120 |
| AT4G34000.1 | c124880_g1_i1 | 67.42 | 5.00E-29 | 112 |
| AT2G23080.1 | c66146_g1_i1 | 61.07 | 4.00E-44 | 158 |
| AT1G69120.1 | c67820_g1_i1 | 64.66 | 3.00E-40 | 145 |
| AT1G69120.1 | c86459_g6_i1 | 42.46 | 4.00E-88 | 285 |
| AT5G16260.1 | c89508_g1_i2 | 43.09 | 3.00E-50 | 179 |
| AT4G16280.2 | c86459_g6_i2 | 46.87 | 1.00E-78 | 259 |
| AT4G34000.1 | c68362_g1_i1 | 45.14 | 1.00E-55 | 197 |
| AT2G06255.1 | c46138_g1_i1 | 59.21 | 1.00E-24 | 98.2 |
| AT1G65480.1 | c88293_g2_i1 | 71.43 | 2.00E-32 | 118 |
| AT5G60100.2 | c94107_g3_i1 | 86 | 2.00E-23 | 94 |
| AT5G65050.3 | c94107_g3_i2 | 86.96 | 5.00E-20 | 85.5 |
| AT4G11880.1 | c95303_g2_i1 | 68.42 | 5.00E-20 | 85.1 |
| AT1G69120.1 | c148413_g1_i1 | 62.86 | 1.00E-21 | 89 |
| AT3G50000.1 | c60451_g1_i1 | 61.25 | 2.00E-27 | 105 |
| AT1G26310.1 | c60451_g1_i2 | 61.25 | 1.00E-27 | 106 |
| AT2G32950.1 | c72918_g2_i3 | 69.49 | 6.00E-22 | 91.7 |
| AT3G57390.1 | c72918_g2_i4 | 69.49 | 3.00E-21 | 91.3 |
| AT3G57390.1 | c93787_g2_i1 | 65.71 | 6.00E-21 | 93.6 |
| AT4G22950.1 | c116231_g1_i1 | 64.41 | 6.00E-20 | 85.1 |
| AT4G22950.1 | c128951_g1_i1 | 63.11 | 2.00E-37 | 130 |
| AT4G22950.1 | c85175_g1_i2 | 44.57 | 2.00E-39 | 143 |
| AT4G22950.1 | c85175_g1_i3 | 44.57 | 3.00E-39 | 143 |
| AT4G22950.1 | c85175_g1_i4 | 44.57 | 2.00E-39 | 143 |
| AT2G43410.2 | c86010_g1_i1 | 57.08 | 4.00E-75 | 236 |
| AT5G16260.1 | c86010_g1_i2 | 65.27 | 3.00E-67 | 216 |
| AT5G13480.2 | c89673_g3_i1 | 55 | 7.00E-57 | 183 |
| AT4G26000.1 | c89673_g4_i1 | 54.46 | 7.00E-68 | 216 |
| AT4G22950.1 | c91178_g1_i2 | 47.62 | 2.00E-44 | 157 |
| AT4G22950.1 | c91178_g3_i1 | 63.89 | 2.00E-28 | 106 |
| AT4G22950.1 | c94107_g4_i1 | 41.18 | 7.00E-33 | 122 |
| AT4G22950.1 | c94107_g4_i2 | 41.18 | 1.00E-20 | 88.6 |
| AT4G22950.1 | c94107_g4_i3 | 40.99 | 1.00E-24 | 102 |
| AT4G22950.1 | c94107_g4_i4 | 39.88 | 6.00E-29 | 112 |
| AT4G22950.1 | c94107_g4_i5 | 33.16 | 6.00E-22 | 93.2 |
| AT4G22950.1 | c94107_g4_i6 | 43.51 | 3.00E-25 | 103 |
| AT4G22950.1 | c99746_g3_i2 | 43.9 | 3.00E-20 | 89 |
| AT4G22950.1 | c99746_g3_i3 | 43.9 | 3.00E-20 | 89 |
| AT1G13260.1 | c58215_g1_i1 | 38.24 | 6.00E-35 | 130 |
| AT2G23380.1 | c72632_g1_i1 | 38.24 | 2.00E-33 | 130 |
| AT5G17690.1 | c81232_g1_i1 | 50.53 | 2.00E-45 | 159 |
| AT5G17690.1 | c83301_g2_i1 | 36.41 | 6.00E-28 | 114 |
| AT1G69120.1 | c77146_g1_i2 | 56 | 2.00E-28 | 108 |
| AT1G69120.1 | c80136_g1_i1 | 41.31 | 9.00E-39 | 142 |
| AT1G69120.1 | c80136_g2_i1 | 51.92 | 6.00E-26 | 102 |
| AT5G67380.1 | c81830_g1_i1 | 52.6 | 2.00E-52 | 175 |
| AT1G69120.1 | c81830_g2_i1 | 52.6 | 4.00E-51 | 175 |
| AT5G16260.1 | c88116_g7_i1 | 69.47 | 3.00E-90 | 270 |
| AT1G65480.1 | c88116_g8_i1 | 77.98 | 4.00E-94 | 283 |
| AT5G47640.1 | c92021_g1_i1 | 79.87 | 1.00E-80 | 266 |
| AT5G60910.1 | c92021_g1_i2 | 62.15 | 1.00E-87 | 286 |
| AT4G36920.1 | c83375_g1_i2 | 46.67 | 4.00E-43 | 156 |
| AT4G36920.1 | c84671_g2_i1 | 64.22 | 2.00E-25 | 107 |
| AT4G36920.1 | c87192_g3_i3 | 68.92 | 2.00E-24 | 104 |
| AT4G36920.1 | c91054_g3_i1 | 57.89 | 1.00E-20 | 89.4 |
| AT1G53090.1 | c97450_g4_i1 | 52.76 | 1.00E-63 | 218 |
| AT1G53090.1 | c97450_g4_i2 | 56.1 | 5.00E-99 | 314 |
| AT5G39660.1 | c97450_g4_i3 | 55.87 | 6.00E-100 | 317 |
| AT2G33810.1 | c97450_g4_i4 | 74.71 | 2.00E-98 | 311 |
| AT2G33810.1 | c97450_g4_i5 | 66.78 | 2.00E-96 | 298 |
| AT1G01060.1 | c52584_g1_i1 | 54.75 | 3.00E-33 | 126 |
| AT2G19520.1 | c89508_g3_i3 | 56.99 | 8.00E-21 | 90.9 |
| AT5G63470.1 | c92844_g1_i1 | 58.61 | 2.00E-89 | 283 |
| AT3G02380.1 | c92844_g1_i2 | 58.61 | 3.00E-89 | 283 |
| AT5G17690.1 | c57275_g1_i1 | 47.27 | 2.00E-30 | 119 |
| AT5G42790.1 | c81684_g2_i1 | 47.3 | 3.00E-30 | 122 |
| AT4G34530.1 | c86316_g1_i1 | 46.49 | 2.00E-26 | 112 |
| AT3G33520.1 | c49456_g2_i2 | 32.67 | 2.00E-32 | 127 |
| AT3G33520.1 | c70813_g1_i1 | 26.88 | 2.00E-36 | 142 |
| AT3G33520.1 | c81390_g2_i1 | 31.66 | 5.00E-48 | 170 |
| AT3G33520.1 | c82970_g1_i1 | 25.92 | 1.00E-31 | 130 |
| AT3G33520.1 | c88638_g1_i1 | 24.63 | 5.00E-26 | 112 |
| AT3G33520.1 | c94438_g1_i1 | 28.75 | 3.00E-32 | 127 |
| AT3G33520.1 | c94438_g1_i2 | 28.75 | 2.00E-32 | 127 |
| AT3G33520.1 | c94438_g3_i1 | 30.17 | 4.00E-55 | 197 |
| AT3G33520.1 | c94438_g3_i2 | 30.17 | 5.00E-55 | 197 |
| AT3G33520.1 | c94438_g4_i1 | 38.28 | 6.00E-24 | 103 |
| AT3G33520.1 | c98416_g1_i1 | 57.42 | 4.00E-59 | 172 |
| AT4G02020.1 | c98416_g1_i10 | 63.33 | 5.00E-165 | 359 |
| AT3G33520.1 | c98416_g1_i11 | 62.42 | 2.00E-71 | 199 |
| AT4G02020.1 | c98416_g1_i12 | 61.12 | 2.00E-167 | 494 |
| AT4G02020.1 | c98416_g1_i2 | 70 | 0 | 599 |
| AT4G16280.2 | c98416_g1_i3 | 62.75 | 0 | 547 |
| AT3G33520.1 | c98416_g1_i4 | 57.42 | 3.00E-48 | 172 |
| AT3G33520.1 | c98416_g1_i7 | 63.07 | 3.00E-117 | 356 |
| AT3G33520.1 | c98416_g1_i9 | 71.88 | 6.00E-89 | 288 |
| AT3G33520.1 | c99151_g2_i1 | 29.11 | 4.00E-37 | 141 |
| AT3G33520.1 | c99151_g2_i2 | 30.64 | 1.00E-56 | 198 |
| AT3G33520.1 | c99151_g6_i1 | 32.3 | 4.00E-35 | 133 |
| AT1G22770.1 | c67037_g1_i3 | 44.41 | 4.00E-50 | 178 |
| AT5G23260.2 | c119609_g1_i1 | 82.86 | 2.00E-32 | 122 |
| AT5G08330.1 | c48328_g1_i1 | 78.26 | 2.00E-30 | 115 |
| AT5G67380.1 | c76265_g1_i1 | 45.7 | 3.00E-34 | 133 |
| AT2G23380.1 | c77980_g1_i1 | 50 | 2.00E-90 | 287 |
| AT5G51230.1 | c137759_g1_i1 | 78.26 | 5.00E-31 | 117 |
| AT1G04400.2 | c142106_g1_i1 | 80.41 | 9.00E-49 | 167 |
| AT3G20740.1 | c856_g1_i1 | 71.08 | 1.00E-27 | 110 |
| AT4G26000.1 | c103329_g1_i1 | 63.33 | 1.00E-26 | 105 |
| AT2G23080.1 | c85121_g1_i1 | 49.05 | 5.00E-91 | 286 |
| AT2G06255.1 | c85121_g2_i1 | 44.14 | 1.00E-35 | 134 |
| AT2G33810.1 | c95831_g2_i2 | 27.75 | 5.00E-23 | 102 |
| AT5G57660.1 | c95831_g2_i4 | 27.75 | 5.00E-23 | 102 |
| AT5G39660.1 | c135780_g1_i1 | 46.81 | 7.00E-32 | 119 |
| AT4G16280.2 | c77146_g1_i1 | 56.73 | 2.00E-56 | 184 |
| AT2G19520.1 | c80388_g1_i2 | 49.69 | 7.00E-42 | 147 |
| AT3G48430.1 | c88116_g6_i1 | 55.75 | 2.00E-53 | 177 |
| AT5G57660.1 | c95202_g1_i1 | 71.76 | 1.00E-32 | 135 |
| AT2G25930.1 | c96605_g3_i2 | 80.88 | 2.00E-30 | 129 |
| AT3G20740.1 | c79151_g2_i1 | 50 | 3.00E-34 | 126 |
| AT5G39660.1 | c85978_g2_i1 | 37.58 | 2.00E-39 | 149 |
| AT5G39660.1 | c89744_g2_i1 | 67.8 | 6.00E-21 | 97.8 |
| AT3G46640.3 | c93553_g3_i1 | 52.6 | 3.00E-97 | 313 |
| AT5G58230.1 | c93553_g3_i3 | 52.34 | 1.00E-96 | 312 |
| AT2G23080.1 | c93553_g3_i4 | 52.34 | 2.00E-96 | 312 |
| AT5G04240.1 | c93553_g3_i5 | 51.17 | 7.00E-90 | 294 |
| AT3G48430.1 | c115823_g1_i1 | 72.88 | 5.00E-21 | 93.6 |
| AT3G47500.1 | c142918_g1_i1 | 64.2 | 2.00E-27 | 111 |
| AT1G65480.1 | c56008_g1_i1 | 85.06 | 4.00E-93 | 300 |
| AT3G47500.1 | c75848_g1_i1 | 56.16 | 7.00E-22 | 99.4 |
| AT3G47500.1 | c80015_g1_i2 | 68.52 | 3.00E-20 | 94.7 |
| AT3G47500.1 | c83407_g2_i2 | 60.53 | 1.00E-23 | 103 |
| AT3G47500.1 | c86120_g1_i2 | 65 | 1.00E-21 | 99.8 |
| AT3G47500.1 | c86120_g1_i3 | 65 | 1.00E-21 | 99.4 |
| AT3G47500.1 | c86263_g1_i3 | 66.67 | 3.00E-23 | 103 |
| AT3G47500.1 | c89744_g2_i2 | 67.24 | 5.00E-21 | 97.8 |
| AT3G47500.1 | c89744_g2_i3 | 67.24 | 3.00E-21 | 95.5 |
| AT3G47500.1 | c89744_g2_i4 | 67.24 | 6.00E-21 | 97.8 |
| AT3G47500.1 | c92107_g2_i1 | 43.35 | 3.00E-62 | 212 |
| AT3G47500.1 | c93553_g1_i1 | 65.12 | 6.00E-50 | 172 |
| AT1G68050.1 | c93553_g3_i2 | 44.24 | 3.00E-92 | 299 |
| AT3G48430.1 | c94376_g3_i1 | 43.75 | 2.00E-23 | 103 |
| AT3G57390.1 | c94376_g4_i1 | 52.08 | 0 | 953 |
| AT5G42790.1 | c94376_g4_i2 | 53.85 | 9.00E-69 | 239 |
| AT3G47500.1 | c96275_g5_i1 | 69.49 | 3.00E-22 | 102 |
| AT3G47500.1 | c98207_g4_i1 | 54.55 | 8.00E-23 | 100 |
| AT3G47500.1 | c98207_g4_i2 | 54.55 | 6.00E-23 | 100 |
| AT3G47500.1 | c98207_g5_i1 | 50 | 4.00E-24 | 99.8 |
| AT3G48430.1 | c98404_g3_i1 | 46.47 | 4.00E-56 | 212 |
| AT3G48430.1 | c98404_g3_i3 | 38.74 | 2.00E-64 | 241 |
| AT3G48430.1 | c98404_g5_i1 | 34.19 | 7.00E-34 | 140 |
| AT4G22950.1 | c75183_g2_i1 | 67.24 | 2.00E-22 | 93.2 |
| AT1G22770.1 | c86323_g7_i1 | 65.08 | 2.00E-22 | 101 |
| AT3G56850.1 | c90235_g1_i1 | 63.79 | 2.00E-21 | 98.2 |
| AT3G50000.1 | c131065_g1_i1 | 85.07 | 1.00E-37 | 129 |
| AT2G46830.1 | c73977_g1_i1 | 55.23 | 8.00E-56 | 180 |
| AT3G18990.1 | c80015_g1_i1 | 60 | 7.00E-22 | 94.7 |
| AT1G69570.1 | c86120_g1_i1 | 65.52 | 5.00E-22 | 95.1 |
| AT5G08330.1 | c79789_g1_i1 | 81.48 | 3.00E-23 | 99.4 |
| AT5G08330.1 | c79789_g1_i2 | 81.48 | 6.00E-23 | 99.4 |
| AT5G42790.1 | c81621_g3_i1 | 69.23 | 8.00E-25 | 99.8 |
| AT5G08330.1 | c86280_g3_i1 | 69.23 | 1.00E-23 | 99.8 |
| AT5G08330.1 | c87443_g4_i1 | 76.27 | 2.00E-23 | 97.8 |
| AT5G08330.1 | c87443_g4_i2 | 75.44 | 2.00E-22 | 96.7 |
| AT5G08330.1 | c87443_g4_i3 | 80.85 | 4.00E-21 | 91.7 |
| AT5G08330.1 | c87443_g4_i4 | 78.18 | 9.00E-23 | 97.8 |
| AT5G08330.1 | c87443_g4_i5 | 81.82 | 3.00E-23 | 101 |
| AT1G13260.1 | c87443_g4_i6 | 81.82 | 6.00E-24 | 101 |
| AT5G08330.1 | c87443_g4_i7 | 81.82 | 3.00E-23 | 100 |
| AT5G08330.1 | c88959_g2_i2 | 77.36 | 8.00E-22 | 95.9 |
| AT5G08330.1 | c92182_g1_i5 | 77.55 | 7.00E-20 | 89.7 |
| AT5G08330.1 | c92182_g2_i1 | 58.75 | 2.00E-21 | 94.4 |
| AT5G08330.1 | c92182_g2_i2 | 58.75 | 2.00E-21 | 94.7 |
| AT5G08330.1 | c92734_g9_i1 | 78.95 | 6.00E-23 | 98.6 |
| AT5G08330.1 | c92734_g9_i2 | 78.95 | 5.00E-23 | 98.6 |
| AT4G26000.1 | c95028_g2_i1 | 70.79 | 1.00E-29 | 120 |
| AT3G18990.1 | c95028_g4_i1 | 86.15 | 2.00E-31 | 116 |
| AT5G08330.1 | c97955_g3_i3 | 79.59 | 9.00E-20 | 90.5 |
| AT4G34530.1 | c21520_g1_i1 | 69 | 7.00E-41 | 144 |
| AT4G34530.1 | c23304_g1_i1 | 64.77 | 1.00E-30 | 115 |
| AT4G34530.1 | c46110_g1_i1 | 76.12 | 1.00E-28 | 109 |
| AT4G34530.1 | c53908_g2_i1 | 70.67 | 1.00E-27 | 110 |
| AT4G34530.1 | c53908_g2_i2 | 70.67 | 2.00E-27 | 107 |
| AT4G34530.1 | c56548_g1_i1 | 72.07 | 6.00E-41 | 147 |
| AT4G34530.1 | c56548_g1_i2 | 65.96 | 1.00E-26 | 106 |
| AT4G34530.1 | c57048_g1_i1 | 80 | 3.00E-43 | 151 |
| AT3G24440.1 | c77723_g1_i1 | 52.34 | 5.00E-60 | 205 |
| AT4G34530.1 | c79628_g1_i1 | 74 | 2.00E-40 | 147 |
| AT4G34530.1 | c79628_g1_i2 | 80 | 6.00E-39 | 143 |
| AT4G34530.1 | c81799_g1_i1 | 63.33 | 7.00E-29 | 117 |
| AT4G34530.1 | c81799_g1_i2 | 63.33 | 3.00E-29 | 117 |
| AT4G34530.1 | c81855_g1_i1 | 69 | 6.00E-39 | 147 |
| AT4G34530.1 | c82044_g1_i1 | 68.18 | 6.00E-21 | 95.1 |
| AT4G34530.1 | c85798_g1_i2 | 65.79 | 1.00E-26 | 104 |
| AT4G34530.1 | c85798_g1_i3 | 68.27 | 3.00E-39 | 148 |
| AT4G34530.1 | c8881_g1_i2 | 85.14 | 4.00E-38 | 135 |
| AT4G34530.1 | c89096_g1_i1 | 67.16 | 7.00E-21 | 95.1 |
| AT4G34530.1 | c90916_g1_i1 | 62.71 | 1.00E-41 | 151 |
| AT4G34530.1 | c90916_g1_i2 | 75.71 | 1.00E-31 | 120 |
| AT4G34530.1 | c90916_g3_i1 | 66.67 | 3.00E-40 | 152 |
| AT4G34530.1 | c91872_g1_i1 | 72.07 | 1.00E-38 | 147 |
| AT4G34530.1 | c91872_g1_i2 | 72.07 | 1.00E-38 | 147 |
| AT4G34530.1 | c91872_g1_i3 | 72.07 | 8.00E-39 | 147 |
| AT4G34530.1 | c91872_g2_i2 | 80 | 2.00E-39 | 146 |
| AT4G34530.1 | c91872_g2_i3 | 80 | 1.00E-38 | 147 |
| AT4G34530.1 | c91960_g1_i1 | 71.21 | 7.00E-21 | 95.9 |
| AT4G34530.1 | c91960_g1_i2 | 71.21 | 7.00E-21 | 95.9 |
| AT4G34530.1 | c92899_g1_i1 | 74 | 7.00E-39 | 147 |
| AT4G34530.1 | c92899_g1_i2 | 80 | 2.00E-37 | 143 |
| AT4G34530.1 | c93832_g1_i1 | 80.77 | 9.00E-39 | 140 |
| AT4G34530.1 | c93832_g4_i1 | 51.2 | 2.00E-42 | 159 |
| AT4G34530.1 | c93832_g4_i2 | 49.65 | 5.00E-31 | 125 |
| AT4G34530.1 | c93832_g4_i4 | 51.2 | 2.00E-42 | 158 |
| AT4G34530.1 | c93832_g4_i5 | 51.2 | 1.00E-42 | 159 |
| AT4G34530.1 | c94404_g2_i1 | 55.19 | 2.00E-41 | 155 |
| AT4G34530.1 | c95677_g7_i1 | 66.67 | 1.00E-39 | 147 |
| AT4G34530.1 | c95677_g7_i4 | 60.95 | 2.00E-28 | 115 |
| AT4G30200.2 | c97506_g1_i1 | 77.66 | 5.00E-48 | 167 |
| AT4G34530.1 | c97506_g2_i1 | 68.64 | 9.00E-47 | 173 |
| AT4G34530.1 | c97506_g2_i2 | 68.64 | 8.00E-47 | 173 |
| AT2G33810.1 | c97506_g2_i3 | 68.64 | 5.00E-47 | 173 |
| AT2G33810.1 | c97506_g2_i4 | 68.64 | 5.00E-47 | 173 |
| AT4G34530.1 | c97506_g2_i5 | 68.64 | 1.00E-46 | 173 |
| AT5G67380.1 | c142719_g1_i1 | 34.03 | 3.00E-20 | 89.4 |
| AT5G67380.1 | c147080_g1_i1 | 92.65 | 2.00E-38 | 137 |
| AT1G69570.1 | c59129_g1_i1 | 91.75 | 8.00E-121 | 355 |
| AT5G67380.1 | c66299_g1_i2 | 91.54 | 1.00E-66 | 213 |
| AT5G67380.1 | c82532_g2_i1 | 86.96 | 2.00E-49 | 169 |
| AT2G18915.2 | c82532_g3_i1 | 92.42 | 1.00E-176 | 499 |
| AT5G67380.1 | c82532_g3_i2 | 88.7 | 6.00E-64 | 224 |
| AT2G25930.1 | c82532_g3_i3 | 86.67 | 3.00E-120 | 366 |
| AT5G67380.1 | c82532_g3_i4 | 92.78 | 4.00E-115 | 355 |
| AT5G67380.1 | c86745_g2_i1 | 31.17 | 1.00E-32 | 132 |
| AT5G35840.1 | c87015_g1_i1 | 86.54 | 0 | 608 |
| AT5G67380.1 | c87587_g6_i1 | 30.89 | 9.00E-24 | 106 |
| AT5G67380.1 | c87587_g6_i2 | 30.21 | 3.00E-23 | 104 |
| AT5G67380.1 | c88142_g1_i1 | 33.46 | 4.00E-34 | 132 |
| AT5G67380.1 | c89026_g2_i1 | 30.72 | 7.00E-34 | 137 |
| AT5G67380.1 | c93390_g4_i2 | 29.18 | 8.00E-20 | 93.2 |
| AT5G67380.1 | c93390_g4_i8 | 29.18 | 4.00E-20 | 93.2 |
| AT5G67380.1 | c95679_g4_i1 | 28.76 | 5.00E-30 | 124 |
| AT5G67380.1 | c95679_g4_i3 | 28.76 | 7.00E-30 | 124 |
| AT5G67380.1 | c97143_g1_i1 | 30.6 | 2.00E-26 | 113 |
| AT5G67380.1 | c97143_g1_i2 | 30.6 | 4.00E-26 | 113 |
| AT5G67380.1 | c98248_g1_i2 | 30.77 | 3.00E-27 | 114 |
| AT5G67380.1 | c98248_g1_i3 | 31.17 | 1.00E-26 | 115 |
| AT5G67380.1 | c98248_g1_i4 | 31.17 | 2.00E-26 | 115 |
| AT5G67380.1 | c98248_g1_i5 | 30.77 | 3.00E-26 | 114 |
| AT3G50000.1 | c85643_g1_i5 | 30.42 | 4.00E-25 | 108 |
| AT3G50000.1 | c89061_g2_i4 | 32.34 | 4.00E-27 | 113 |
| AT3G50000.1 | c92765_g1_i2 | 26.26 | 3.00E-26 | 114 |
| AT3G50000.1 | c92765_g1_i4 | 26.26 | 8.00E-27 | 114 |
| AT3G50000.1 | c92765_g1_i5 | 26.26 | 3.00E-26 | 114 |
| AT3G50000.1 | c92765_g1_i6 | 26.26 | 1.00E-26 | 114 |
| AT3G50000.1 | c92765_g1_i8 | 26.26 | 6.00E-27 | 114 |
| AT3G50000.1 | c93001_g2_i2 | 28.04 | 1.00E-21 | 99.8 |
| AT3G46640.3 | c94084_g1_i1 | 32.68 | 1.00E-28 | 120 |
| AT3G56850.1 | c94084_g1_i2 | 32.68 | 2.00E-29 | 121 |
| AT3G46640.3 | c94084_g1_i3 | 32.68 | 1.00E-28 | 120 |
| AT1G35160.2 | c94179_g1_i2 | 28.1 | 2.00E-29 | 124 |
| AT3G50000.1 | c94310_g2_i1 | 26.98 | 4.00E-24 | 107 |
| AT3G50000.1 | c94310_g2_i2 | 25.85 | 2.00E-23 | 105 |
| AT3G50000.1 | c94310_g2_i5 | 26.98 | 8.00E-24 | 106 |
| AT3G50000.1 | c94310_g2_i6 | 25.7 | 4.00E-24 | 107 |
| AT3G50000.1 | c95679_g4_i2 | 28.04 | 1.00E-28 | 120 |
| AT3G50000.1 | c95679_g4_i5 | 28.04 | 2.00E-28 | 120 |
| AT3G50000.1 | c97885_g4_i1 | 27.8 | 1.00E-21 | 99.8 |
| AT2G23080.1 | c31321_g1_i1 | 90 | 6.00E-31 | 115 |
| AT2G23080.1 | c60918_g1_i1 | 30.48 | 4.00E-21 | 92 |
| AT5G24470.1 | c77654_g1_i1 | 28.75 | 9.00E-37 | 140 |
| AT2G23080.1 | c80828_g1_i1 | 30 | 9.00E-21 | 92.4 |
| AT2G23080.1 | c81821_g1_i2 | 28.19 | 1.00E-26 | 114 |
| AT2G23080.1 | c81869_g1_i1 | 28.62 | 2.00E-28 | 118 |
| AT2G23080.1 | c82498_g1_i1 | 26.65 | 3.00E-24 | 106 |
| AT2G23080.1 | c83797_g2_i1 | 32.13 | 1.00E-26 | 111 |
| AT2G23080.1 | c83797_g2_i2 | 32.13 | 2.00E-26 | 111 |
| AT2G23080.1 | c84369_g1_i1 | 27.64 | 3.00E-20 | 93.6 |
| AT1G18100.1 | c84766_g4_i1 | 33.44 | 1.00E-36 | 141 |
| AT5G04240.1 | c84766_g4_i2 | 33.77 | 3.00E-37 | 142 |
| AT2G23080.1 | c85616_g2_i1 | 27.24 | 2.00E-20 | 94.7 |
| AT2G23080.1 | c85616_g2_i2 | 27.24 | 3.00E-20 | 94.4 |
| AT2G23080.1 | c85802_g2_i1 | 29.61 | 2.00E-30 | 124 |
| AT2G23080.1 | c87497_g1_i1 | 33.33 | 3.00E-30 | 124 |
| AT2G23080.1 | c87497_g1_i2 | 33.33 | 2.00E-30 | 124 |
| AT2G23080.1 | c87587_g5_i1 | 26.96 | 8.00E-24 | 105 |
| AT2G23080.1 | c88142_g5_i1 | 32.57 | 5.00E-34 | 134 |
| AT2G32950.1 | c88142_g6_i1 | 32.24 | 4.00E-35 | 136 |
| AT2G23080.1 | c89770_g1_i2 | 27.3 | 9.00E-21 | 95.9 |
| AT2G23080.1 | c89770_g1_i3 | 27.19 | 3.00E-21 | 96.7 |
| AT2G23080.1 | c89770_g1_i4 | 26.62 | 5.00E-20 | 93.6 |
| AT2G23080.1 | c89869_g1_i1 | 25.56 | 5.00E-20 | 92.8 |
| AT2G23080.1 | c93264_g1_i1 | 28.76 | 5.00E-28 | 118 |
| AT2G23080.1 | c93608_g1_i2 | 32.08 | 2.00E-31 | 127 |
| AT2G23080.1 | c93746_g2_i3 | 31.48 | 2.00E-20 | 92.8 |
| AT2G23080.1 | c93746_g2_i4 | 29.51 | 5.00E-29 | 119 |
| AT2G23080.1 | c94310_g2_i3 | 25.83 | 6.00E-20 | 93.6 |
| AT2G23080.1 | c94310_g2_i4 | 25.83 | 6.00E-20 | 93.6 |
| AT2G23080.1 | c95328_g1_i3 | 30.19 | 6.00E-35 | 135 |
| AT2G23080.1 | c95679_g4_i6 | 29.19 | 1.00E-30 | 123 |
| AT2G23080.1 | c96748_g2_i1 | 28.81 | 1.00E-29 | 123 |
| AT2G23080.1 | c98469_g4_i1 | 28.15 | 5.00E-28 | 118 |
| AT2G23070.1 | c49567_g1_i1 | 31.15 | 7.00E-22 | 95.5 |
| AT2G23070.1 | c81821_g1_i3 | 31.38 | 5.00E-25 | 110 |
| AT2G23070.1 | c87222_g3_i1 | 30.54 | 2.00E-27 | 117 |
| AT4G26000.1 | c87575_g1_i1 | 91.56 | 6.00E-140 | 418 |
| AT1G68050.1 | c87575_g1_i2 | 90.75 | 0 | 623 |
| AT1G68050.1 | c87575_g1_i3 | 91.56 | 6.00E-140 | 418 |
| AT1G68050.1 | c87575_g3_i1 | 92.31 | 3.00E-114 | 340 |
| AT2G23070.1 | c92765_g1_i3 | 29.7 | 7.00E-22 | 99 |
| AT2G23070.1 | c94179_g1_i3 | 28.28 | 3.00E-29 | 123 |
| AT2G23070.1 | c94179_g1_i4 | 28.34 | 2.00E-29 | 124 |
| AT2G23070.1 | c95615_g4_i1 | 32.9 | 1.00E-29 | 125 |
| AT2G23070.1 | c97231_g4_i2 | 32.07 | 7.00E-27 | 115 |
| AT2G23070.1 | c97231_g4_i3 | 32.07 | 7.00E-27 | 115 |
| AT2G23070.1 | c97231_g4_i4 | 32.07 | 6.00E-27 | 116 |
| AT2G23070.1 | c97231_g4_i5 | 32.07 | 5.00E-27 | 116 |
| AT2G23070.1 | c98469_g3_i1 | 35.26 | 8.00E-22 | 100 |
| AT3G12810.1 | c112493_g1_i1 | 55.2 | 4.00E-35 | 134 |
| AT1G01060.1 | c128931_g1_i1 | 75 | 4.00E-31 | 121 |
| AT2G23380.1 | c59210_g1_i1 | 50.7 | 4.00E-30 | 120 |
| AT2G23380.1 | c90207_g1_i1 | 51.55 | 2.00E-23 | 100 |
| AT1G49720.2 | c90207_g2_i2 | 60.29 | 0 | 694 |
| AT1G22770.1 | c90207_g2_i3 | 58.99 | 0 | 899 |
| AT2G32950.1 | c103277_g1_i1 | 83.56 | 8.00E-39 | 141 |
| AT2G32950.1 | c117962_g1_i1 | 90.59 | 2.00E-48 | 167 |
| AT2G32950.1 | c138098_g1_i1 | 82.81 | 4.00E-30 | 118 |
| AT2G23380.1 | c35534_g1_i1 | 70.07 | 2.00E-145 | 432 |
| AT5G04240.1 | c76422_g1_i1 | 76.54 | 0 | 1085 |
| AT3G50000.1 | c76422_g1_i2 | 75.22 | 0 | 1076 |
| AT3G50000.1 | c76422_g1_i3 | 72.68 | 0 | 796 |
| AT2G32950.1 | c83784_g1_i1 | 36.68 | 7.00E-58 | 208 |
| AT2G32950.1 | c95738_g1_i1 | 85 | 8.00E-131 | 393 |
| AT2G32950.1 | c95738_g1_i2 | 92.68 | 3.00E-103 | 323 |
| AT2G32950.1 | c95738_g1_i3 | 84.38 | 8.00E-134 | 398 |
| AT2G32950.1 | c95738_g2_i1 | 57.36 | 7.00E-37 | 150 |
| AT2G32950.1 | c95738_g2_i2 | 56.18 | 1.00E-51 | 191 |
| AT2G32950.1 | c95738_g2_i3 | 76.81 | 1.00E-33 | 129 |
| AT2G32950.1 | c95738_g2_i4 | 58.02 | 6.00E-40 | 149 |
| AT2G32950.1 | c95738_g2_i5 | 57.36 | 7.00E-37 | 150 |
| AT2G32950.1 | c95738_g2_i6 | 49.19 | 5.00E-23 | 107 |
| AT2G32950.1 | c95738_g2_i7 | 57.33 | 7.00E-110 | 340 |
| AT2G32950.1 | c95738_g3_i1 | 81.82 | 5.00E-93 | 294 |
| AT4G08920.1 | c110638_g1_i1 | 76.25 | 5.00E-36 | 134 |
| AT4G08920.1 | c111288_g1_i1 | 86.3 | 1.00E-35 | 132 |
| AT4G08920.1 | c111818_g1_i1 | 100 | 2.00E-45 | 159 |
| AT4G08920.1 | c131633_g1_i1 | 94.03 | 3.00E-36 | 134 |
| AT4G08920.1 | c132787_g1_i1 | 85.92 | 2.00E-33 | 126 |
| AT5G39660.1 | c64241_g1_i1 | 85.27 | 0 | 689 |
| AT4G08920.1 | c7322_g1_i1 | 60 | 1.00E-27 | 110 |
| AT2G25930.1 | c82826_g2_i1 | 85.91 | 3.00E-126 | 379 |
| AT4G08920.1 | c82826_g2_i2 | 87.1 | 8.00E-81 | 264 |
| AT4G08920.1 | c82826_g3_i1 | 91.34 | 1.00E-72 | 238 |
| AT4G08920.1 | c87509_g2_i1 | 47.87 | 1.00E-34 | 142 |
| AT4G08920.1 | c87509_g2_i2 | 62.62 | 3.00E-114 | 358 |
| AT4G34530.1 | c99751_g2_i1 | 88.97 | 0 | 719 |
| AT3G18990.1 | c99751_g2_i2 | 79.06 | 0 | 1008 |
| AT4G08920.1 | c99751_g2_i3 | 55.3 | 4.00E-46 | 167 |
| AT3G18990.1 | c99751_g2_i4 | 79.06 | 0 | 1008 |
| AT4G08920.1 | c99751_g5_i1 | 90.24 | 2.00E-73 | 238 |
| AT1G04400.2 | c123848_g1_i1 | 92.65 | 3.00E-38 | 138 |
| AT1G04400.2 | c34724_g1_i1 | 59.09 | 2.00E-29 | 115 |
| AT5G65050.3 | c70927_g1_i3 | 68.97 | 2.00E-123 | 374 |
| AT3G25730.1 | c94698_g2_i1 | 65.14 | 0 | 791 |
| AT1G04400.2 | c96538_g1_i2 | 28.74 | 8.00E-50 | 187 |
| AT2G46790.1 | c96538_g1_i3 | 28.74 | 7.00E-50 | 187 |
| AT1G50680.1 | c96538_g1_i4 | 28.74 | 5.00E-50 | 187 |
| AT1G04400.2 | c99616_g1_i1 | 45.45 | 2.00E-22 | 97.1 |
| AT1G04400.2 | c99616_g2_i1 | 33.59 | 3.00E-23 | 107 |
| AT1G04400.2 | c99616_g2_i2 | 28.33 | 8.00E-28 | 121 |
| AT1G77300.1 | c126993_g1_i1 | 58.03 | 2.00E-57 | 202 |
| AT1G77300.1 | c14941_g1_i1 | 75.74 | 9.00E-70 | 239 |
| AT1G77300.1 | c86820_g1_i2 | 41.98 | 1.00E-29 | 123 |
| AT1G77300.1 | c92516_g1_i1 | 47.22 | 5.00E-24 | 112 |
| AT1G77300.1 | c92516_g1_i10 | 39.81 | 1.00E-39 | 160 |
| AT1G77300.1 | c92516_g1_i11 | 45.59 | 2.00E-29 | 129 |
| AT1G77300.1 | c92516_g1_i12 | 39.81 | 2.00E-39 | 160 |
| AT1G77300.1 | c92516_g1_i13 | 39.81 | 2.00E-40 | 159 |
| AT1G77300.1 | c92516_g1_i14 | 39.9 | 1.00E-37 | 153 |
| AT1G77300.1 | c92516_g1_i2 | 39.81 | 6.00E-40 | 160 |
| AT1G77300.1 | c92516_g1_i3 | 45.59 | 5.00E-31 | 129 |
| AT1G77300.1 | c92516_g1_i4 | 45.59 | 2.00E-29 | 129 |
| AT1G77300.1 | c92516_g1_i5 | 45.59 | 3.00E-30 | 129 |
| AT1G77300.1 | c92516_g1_i6 | 39.81 | 2.00E-39 | 160 |
| AT1G77300.1 | c92516_g1_i7 | 47.22 | 3.00E-24 | 112 |
| AT1G77300.1 | c92516_g1_i8 | 39.81 | 2.00E-40 | 159 |
| AT1G77300.1 | c92516_g1_i9 | 45.59 | 2.00E-29 | 129 |
| AT1G77300.1 | c92516_g3_i1 | 46.46 | 2.00E-31 | 125 |
| AT1G77300.1 | c94437_g2_i1 | 33.71 | 6.00E-21 | 103 |
| AT1G77300.1 | c94437_g2_i2 | 33.71 | 5.00E-21 | 103 |
| AT1G77300.1 | c94437_g2_i3 | 33.71 | 6.00E-21 | 103 |
| AT1G77300.1 | c98840_g2_i1 | 44.91 | 1.00E-52 | 202 |
| AT1G77300.1 | c98840_g2_i3 | 44.91 | 2.00E-52 | 202 |
| AT1G77300.1 | c98840_g2_i4 | 44.19 | 1.00E-51 | 202 |
| AT1G77300.1 | c98840_g2_i5 | 44.19 | 4.00E-52 | 200 |
| AT1G77300.1 | c98840_g2_i7 | 44.91 | 7.00E-52 | 202 |
| AT1G77300.1 | c98840_g2_i8 | 44.91 | 1.00E-51 | 201 |
| AT1G77300.1 | c99621_g4_i1 | 35 | 3.00E-20 | 100 |
| AT1G77300.1 | c99621_g4_i3 | 34.97 | 8.00E-21 | 102 |
| AT1G77300.1 | c99621_g4_i6 | 35 | 3.00E-20 | 100 |
| AT1G77300.1 | c99621_g4_i7 | 34.97 | 8.00E-21 | 102 |
| AT1G77300.1 | c99621_g4_i8 | 34.97 | 8.00E-21 | 102 |
| AT3G33520.1 | c99784_g1_i1 | 64 | 2.00E-140 | 485 |
| AT1G77300.1 | c99784_g1_i2 | 71.16 | 4.00E-101 | 345 |
| AT5G24470.1 | c99784_g1_i3 | 64 | 3.00E-140 | 485 |
| AT3G24440.1 | c99784_g1_i4 | 64 | 3.00E-140 | 485 |
| AT3G24440.1 | c99784_g1_i5 | 64 | 2.00E-140 | 485 |
| AT4G30200.2 | c99653_g1_i1 | 33.24 | 2.00E-26 | 114 |
| AT3G15354.1 | c99653_g1_i2 | 48.23 | 4.00E-30 | 126 |
| AT4G30200.2 | c99653_g1_i3 | 42.31 | 7.00E-24 | 110 |
| AT5G57380.1 | c99653_g2_i1 | 42.31 | 5.00E-25 | 110 |
| AT5G57360.2 | c70743_g2_i1 | 62.65 | 1.00E-26 | 102 |
| AT2G40080.1 | c93803_g1_i1 | 66.22 | 5.00E-25 | 102 |
| AT2G40080.1 | c93803_g1_i2 | 66.22 | 1.00E-25 | 102 |
| AT2G40080.1 | c93803_g1_i3 | 66.22 | 6.00E-25 | 102 |
| AT1G49720.2 | c93803_g1_i4 | 66.22 | 2.00E-26 | 102 |
| AT5G35840.1 | c95520_g1_i1 | 36.2 | 6.00E-38 | 151 |
| AT4G08920.1 | c95520_g1_i2 | 36.2 | 8.00E-38 | 151 |
| AT5G02810.1 | c30668_g1_i1 | 75.45 | 1.00E-49 | 158 |
| AT1G17455.1 | c77345_g1_i1 | 70.18 | 4.00E-47 | 159 |
| AT3G15354.1 | c91390_g4_i1 | 74.56 | 3.00E-49 | 163 |
| AT1G35160.2 | c91390_g4_i2 | 74.56 | 2.00E-49 | 163 |
| AT5G10450.4 | c91390_g4_i3 | 74.56 | 5.00E-50 | 162 |
| AT3G24440.1 | c95875_g2_i3 | 43.33 | 4.00E-37 | 154 |
| AT5G04240.1 | c95875_g2_i4 | 43.33 | 5.00E-37 | 154 |
| AT5G04240.1 | c95875_g2_i5 | 43.33 | 5.00E-37 | 154 |
| AT5G04240.1 | c95875_g2_i6 | 43.33 | 4.00E-37 | 154 |
| AT5G04240.1 | c97312_g2_i1 | 38.43 | 1.00E-36 | 154 |
| AT5G04240.1 | c97312_g2_i10 | 38.43 | 9.00E-37 | 154 |
| AT5G04240.1 | c97312_g2_i11 | 38.43 | 9.00E-37 | 154 |
| AT5G04240.1 | c97312_g2_i14 | 38.43 | 1.00E-36 | 154 |
| AT5G04240.1 | c97312_g2_i2 | 38.43 | 7.00E-37 | 154 |
| AT5G04240.1 | c97312_g2_i3 | 38.43 | 1.00E-36 | 154 |
| AT5G04240.1 | c97312_g2_i4 | 38.43 | 9.00E-37 | 154 |
| AT5G04240.1 | c97312_g2_i5 | 38.43 | 9.00E-37 | 154 |
| AT5G04240.1 | c97312_g2_i6 | 38.43 | 9.00E-37 | 154 |
| AT5G04240.1 | c97312_g2_i7 | 38.43 | 1.00E-36 | 154 |
| AT5G04240.1 | c97312_g2_i8 | 38.43 | 1.00E-36 | 154 |
| AT5G04240.1 | c97312_g2_i9 | 38.43 | 1.00E-36 | 154 |
| AT5G04240.1 | c98947_g5_i1 | 31.73 | 1.00E-29 | 130 |
| AT5G04240.1 | c98947_g5_i2 | 31.73 | 1.00E-29 | 130 |
| AT5G04240.1 | c98947_g5_i4 | 31.73 | 1.00E-29 | 130 |
| AT5G04240.1 | c98947_g5_i5 | 31.73 | 1.00E-29 | 130 |
| AT5G04240.1 | c98947_g5_i7 | 31.73 | 1.00E-29 | 130 |
| AT4G20370.1 | c99119_g2_i1 | 39.82 | 2.00E-38 | 159 |
| AT1G18100.1 | c99119_g2_i2 | 39.82 | 2.00E-38 | 159 |
| AT1G09570.1 | c99369_g1_i1 | 63.85 | 0 | 723 |
| AT4G34000.1 | c80912_g1_i1 | 68.7 | 3.00E-41 | 145 |
| AT4G08920.1 | c80912_g1_i2 | 68.7 | 3.00E-41 | 145 |
| AT4G08920.1 | c80912_g1_i3 | 68.7 | 3.00E-42 | 145 |
| AT5G16260.1 | c41273_g1_i1 | 59.38 | 1.00E-27 | 111 |
| AT2G40080.1 | c95120_g2_i1 | 69.81 | 2.00E-78 | 251 |
| AT5G16260.1 | c95714_g2_i1 | 41.78 | 3.00E-24 | 101 |
| AT3G12810.1 | c95714_g3_i2 | 62.71 | 2.00E-119 | 369 |
| AT1G26310.1 | c95714_g3_i4 | 58.7 | 2.00E-147 | 444 |
| AT5G02810.1 | c95714_g3_i5 | 58.87 | 2.00E-66 | 223 |
| AT5G51230.1 | c33841_g1_i1 | 81.82 | 1.00E-47 | 166 |
| AT2G23080.1 | c93569_g2_i1 | 62.06 | 0 | 637 |
| AT1G01060.1 | c93569_g2_i2 | 61.78 | 0 | 644 |
| AT1G01060.1 | c93569_g2_i3 | 65.79 | 1.00E-99 | 316 |
| AT2G18915.2 | c93569_g2_i4 | 61.78 | 0 | 644 |
| AT2G43410.2 | c101592_g1_i1 | 77.34 | 8.00E-106 | 327 |
| AT4G16280.2 | c104433_g1_i1 | 59.7 | 2.00E-23 | 99.8 |
| AT4G16280.2 | c135168_g1_i1 | 60.49 | 1.00E-26 | 108 |
| AT4G36920.1 | c80089_g4_i1 | 55.98 | 3.00E-71 | 249 |
| AT4G16280.2 | c80089_g4_i2 | 55.98 | 5.00E-71 | 249 |
| AT4G16280.2 | c84999_g1_i1 | 74.67 | 3.00E-24 | 109 |
| AT5G08330.1 | c84999_g1_i2 | 50.29 | 1.00E-83 | 295 |
| AT2G06255.1 | c84999_g1_i3 | 54.55 | 2.00E-121 | 393 |
| AT4G16280.2 | c87828_g4_i1 | 32.49 | 4.00E-20 | 97.1 |
| AT4G16280.2 | c92711_g7_i1 | 43.14 | 3.00E-21 | 98.2 |
| AT4G16280.2 | c92711_g9_i1 | 52.04 | 2.00E-25 | 105 |
| AT4G16280.2 | c97265_g1_i1 | 45.76 | 3.00E-45 | 174 |
| AT4G16280.2 | c97265_g1_i2 | 45.76 | 2.00E-44 | 174 |
| AT4G16280.2 | c97265_g1_i3 | 45.76 | 5.00E-46 | 173 |
| AT4G16280.2 | c97265_g1_i4 | 45.76 | 4.00E-45 | 174 |
| AT4G16280.2 | c97265_g1_i5 | 45.76 | 2.00E-44 | 174 |
| AT4G16280.2 | c97265_g1_i6 | 45.76 | 1.00E-44 | 174 |
| AT4G16280.2 | c97265_g1_i7 | 45.76 | 2.00E-44 | 174 |
| AT1G78300.1 | c66694_g1_i2 | 56.38 | 1.00E-37 | 137 |
| AT5G23260.2 | c75407_g1_i2 | 43.23 | 4.00E-34 | 127 |
| AT5G65050.3 | c94134_g4_i1 | 76.56 | 3.00E-143 | 431 |
| AT5G63470.1 | c94134_g4_i2 | 75.27 | 0 | 609 |
| AT5G65080.1 | c94134_g6_i1 | 73.06 | 8.00E-104 | 310 |
| AT1G68050.1 | c96634_g1_i1 | 45.45 | 2.00E-23 | 105 |
| AT1G68050.1 | c96634_g1_i2 | 45.45 | 2.00E-23 | 105 |
| AT1G68050.1 | c96634_g2_i1 | 45.45 | 7.00E-22 | 103 |
| AT1G68050.1 | c96634_g2_i3 | 45.45 | 5.00E-22 | 103 |
| AT1G68050.1 | c96634_g2_i4 | 45.45 | 6.00E-22 | 103 |
| AT1G68050.1 | c96634_g2_i5 | 45.45 | 6.00E-22 | 103 |
| AT5G02810.1 | c96828_g1_i1 | 83.79 | 0 | 959 |
| AT5G67180.1 | c96828_g1_i2 | 83.79 | 0 | 955 |
| AT2G45660.1 | c96828_g2_i1 | 83.8 | 6.00E-77 | 256 |
| AT2G45660.1 | c96828_g2_i2 | 83.8 | 7.00E-79 | 253 |
| AT1G68050.1 | c97877_g1_i1 | 44.55 | 6.00E-20 | 97.1 |
| AT1G68050.1 | c97877_g1_i2 | 44.55 | 6.00E-20 | 97.1 |
| AT3G10390.1 | c100245_g2_i1 | 39.11 | 1.00E-82 | 296 |
| AT3G10390.1 | c12083_g1_i1 | 83.94 | 4.00E-126 | 384 |
| AT3G10390.1 | c144375_g1_i1 | 47.06 | 4.00E-21 | 95.9 |
| AT3G10390.1 | c88640_g9_i1 | 23.66 | 3.00E-21 | 101 |
| AT3G10390.1 | c89277_g1_i1 | 31.34 | 3.00E-39 | 158 |
| AT3G10390.1 | c89277_g1_i2 | 30.37 | 6.00E-31 | 133 |
| AT3G10390.1 | c89277_g1_i3 | 30.8 | 7.00E-25 | 113 |
| AT3G10390.1 | c89277_g1_i4 | 30.37 | 6.00E-31 | 133 |
| AT5G65050.3 | c96271_g1_i1 | 56.87 | 0 | 706 |
| AT3G10390.1 | c96271_g2_i1 | 66.67 | 8.00E-24 | 100 |
| AT3G10390.1 | c96271_g3_i1 | 69.06 | 1.00E-116 | 361 |
| AT4G36920.1 | c97418_g1_i1 | 56.82 | 0 | 369 |
| AT3G10390.1 | c97418_g1_i2 | 46.76 | 2.00E-43 | 165 |
| AT5G62040.1 | c99951_g3_i1 | 76.37 | 0 | 1081 |
| AT5G57360.2 | c99951_g3_i3 | 76.37 | 0 | 1083 |
| AT3G10390.1 | c99951_g8_i1 | 73.97 | 1.00E-32 | 125 |
| AT3G04610.1 | c100252_g5_i1 | 35.63 | 2.00E-23 | 107 |
| AT4G11880.1 | c100359_g1_i1 | 66.29 | 4.00E-169 | 501 |
| AT3G04610.1 | c135272_g1_i1 | 68.54 | 1.00E-22 | 96.7 |
| AT3G04610.1 | c46560_g1_i1 | 32.16 | 2.00E-20 | 93.6 |
| AT3G04610.1 | c66381_g1_i3 | 32.84 | 4.00E-25 | 108 |
| AT3G04610.1 | c69398_g1_i1 | 58.88 | 4.00E-67 | 223 |
| AT3G04610.1 | c69904_g1_i1 | 30.11 | 8.00E-23 | 100 |
| AT3G04610.1 | c73101_g1_i1 | 35.63 | 4.00E-25 | 108 |
| AT3G04610.1 | c73470_g1_i1 | 31.49 | 2.00E-20 | 94 |
| AT1G26310.1 | c81479_g2_i1 | 56.49 | 2.00E-132 | 407 |
| AT1G04400.2 | c81479_g2_i2 | 56.14 | 2.00E-131 | 405 |
| AT3G04610.1 | c88931_g1_i2 | 25.42 | 4.00E-20 | 97.4 |
| AT3G04610.1 | c91122_g1_i1 | 37.21 | 3.00E-26 | 116 |
| AT3G04610.1 | c91122_g1_i2 | 37.21 | 2.00E-26 | 116 |
| AT3G04610.1 | c91122_g1_i3 | 37.21 | 3.00E-26 | 116 |
| AT3G04610.1 | c91122_g1_i4 | 36.63 | 2.00E-26 | 116 |
| AT3G04610.1 | c91122_g1_i6 | 37.21 | 2.00E-26 | 116 |
| AT3G04610.1 | c92308_g1_i1 | 33.64 | 5.00E-24 | 108 |
| AT3G04610.1 | c92308_g1_i2 | 33.64 | 7.00E-24 | 108 |
| AT3G04610.1 | c92308_g1_i3 | 28.46 | 1.00E-25 | 114 |
| AT3G04610.1 | c95770_g2_i1 | 32.84 | 6.00E-23 | 105 |
| AT3G02520.1 | c95779_g3_i1 | 69.83 | 3.00E-74 | 250 |
| AT3G04610.1 | c97816_g1_i1 | 27.38 | 3.00E-20 | 97.4 |
| AT3G04610.1 | c97816_g1_i2 | 27.38 | 3.00E-20 | 97.4 |
| AT3G04610.1 | c97816_g1_i3 | 27.38 | 3.00E-20 | 97.4 |
| AT2G43410.2 | c128325_g1_i1 | 56.38 | 3.00E-28 | 115 |
| AT2G43410.2 | c134091_g1_i1 | 55.95 | 3.00E-22 | 96.7 |
| AT2G43410.2 | c31677_g1_i1 | 56.76 | 4.00E-22 | 95.5 |
| AT2G43410.2 | c82364_g2_i4 | 35.21 | 7.00E-30 | 109 |
| AT2G18915.2 | c97189_g1_i5 | 38.15 | 2.00E-54 | 204 |
| AT2G43410.2 | c99051_g1_i2 | 59.26 | 2.00E-34 | 144 |
| AT2G43410.2 | c99051_g1_i3 | 59.26 | 2.00E-34 | 144 |
| AT2G43410.2 | c99051_g2_i1 | 34.11 | 1.00E-25 | 110 |
| AT5G63470.1 | c99819_g1_i1 | 42.28 | 0 | 612 |
| AT5G57360.2 | c99819_g1_i2 | 42.28 | 0 | 612 |
| AT5G57360.2 | c99819_g1_i3 | 42.28 | 0 | 612 |
| AT2G43410.2 | c99819_g2_i1 | 71.11 | 1.00E-36 | 139 |
| AT1G68840.1 | c95416_g3_i1 | 41.48 | 5.00E-58 | 203 |
| AT5G08330.1 | c102867_g1_i1 | 78.12 | 4.00E-53 | 170 |
| AT1G65480.1 | c59813_g1_i2 | 75.64 | 2.00E-39 | 135 |
| AT5G16260.1 | c84088_g2_i1 | 76.92 | 5.00E-95 | 285 |
| AT1G65480.1 | c84088_g2_i2 | 65.96 | 4.00E-37 | 135 |
| AT2G40080.1 | c84088_g2_i3 | 76.92 | 5.00E-95 | 285 |
| AT1G65480.1 | c84088_g2_i4 | 73.49 | 3.00E-37 | 132 |
| AT1G17455.1 | c84088_g2_i5 | 76.92 | 1.00E-94 | 284 |
| AT1G65480.1 | c84088_g2_i6 | 73.49 | 3.00E-37 | 132 |
| AT5G60910.1 | c11865_g1_i1 | 39.46 | 8.00E-31 | 115 |
| AT5G60910.1 | c145968_g1_i1 | 42.65 | 8.00E-32 | 118 |
| AT5G60910.1 | c59357_g1_i1 | 93.55 | 4.00E-35 | 125 |
| AT5G13480.2 | c61614_g1_i1 | 52.38 | 6.00E-50 | 167 |
| AT5G60910.1 | c61685_g1_i1 | 33.65 | 1.00E-30 | 119 |
| AT5G60910.1 | c65987_g1_i1 | 72.73 | 1.00E-29 | 113 |
| AT5G60910.1 | c67980_g1_i1 | 39.39 | 5.00E-36 | 132 |
| AT5G60910.1 | c68983_g1_i1 | 72.73 | 1.00E-30 | 113 |
| AT5G60910.1 | c72752_g1_i1 | 45.25 | 1.00E-38 | 140 |
| AT5G60910.1 | c72918_g2_i1 | 45.35 | 3.00E-43 | 151 |
| AT5G60910.1 | c75036_g1_i1 | 41.28 | 2.00E-35 | 134 |
| AT5G60910.1 | c77424_g2_i1 | 43.82 | 3.00E-43 | 154 |
| AT5G57660.1 | c77424_g2_i2 | 43.82 | 9.00E-44 | 154 |
| AT5G60910.1 | c79125_g1_i1 | 39.39 | 2.00E-35 | 132 |
| AT5G60910.1 | c79125_g1_i2 | 45.03 | 8.00E-34 | 128 |
| AT5G60910.1 | c85175_g1_i6 | 41.86 | 6.00E-38 | 141 |
| AT2G34140.1 | c88116_g1_i1 | 58.23 | 5.00E-91 | 280 |
| AT2G43410.2 | c88116_g3_i1 | 42.4 | 3.00E-53 | 182 |
| AT5G60910.1 | c88293_g4_i2 | 47.83 | 7.00E-43 | 155 |
| AT5G60910.1 | c88293_g4_i3 | 46.56 | 8.00E-41 | 149 |
| AT5G60910.1 | c90323_g1_i1 | 38.85 | 2.00E-28 | 112 |
| AT5G60910.1 | c90323_g1_i3 | 38.85 | 1.00E-28 | 112 |
| AT5G60910.1 | c90323_g1_i4 | 39.6 | 1.00E-27 | 110 |
| AT5G60910.1 | c92220_g3_i1 | 93.55 | 4.00E-35 | 125 |
| AT4G11880.1 | c20397_g1_i1 | 88.64 | 2.00E-110 | 331 |
| AT3G11540.1 | c74059_g1_i1 | 82.94 | 3.00E-125 | 372 |
| AT2G19520.1 | c90553_g1_i1 | 63.3 | 2.00E-36 | 134 |
| AT1G78300.1 | c90553_g2_i1 | 78.87 | 0 | 762 |
| AT3G57390.1 | c90553_g3_i1 | 77.83 | 0 | 767 |
| AT2G19520.1 | c90553_g5_i1 | 76.42 | 6.00E-55 | 185 |
| AT3G47500.1 | c130196_g1_i1 | 84.08 | 2.00E-91 | 285 |
| AT5G13480.2 | c83399_g1_i1 | 26.91 | 4.00E-22 | 103 |
| AT5G13480.2 | c84941_g1_i1 | 25.42 | 1.00E-20 | 98.2 |
| AT5G13480.2 | c88585_g1_i1 | 26.4 | 3.00E-20 | 95.5 |
| AT5G13480.2 | c92912_g1_i1 | 31.12 | 7.00E-23 | 105 |
| AT1G69570.1 | c92912_g1_i2 | 31.12 | 5.00E-23 | 106 |
| AT5G13480.2 | c93342_g1_i2 | 26.03 | 2.00E-20 | 98.2 |
| AT5G13480.2 | c95750_g6_i2 | 28.71 | 1.00E-21 | 102 |
| AT5G13480.2 | c95750_g6_i3 | 28.71 | 1.00E-21 | 102 |
| AT5G13480.2 | c95750_g6_i4 | 28.71 | 1.00E-21 | 102 |
| AT5G67380.1 | c95783_g1_i1 | 26.3 | 5.00E-23 | 106 |
| AT5G02810.1 | c95783_g1_i2 | 26.3 | 5.00E-23 | 106 |
| AT1G22770.1 | c101470_g1_i1 | 89.17 | 1.00E-67 | 227 |
| AT1G22770.1 | c122108_g1_i1 | 95.52 | 6.00E-34 | 129 |
| AT1G22770.1 | c99108_g1_i1 | 80.46 | 1.00E-42 | 155 |
| AT1G22770.1 | c99108_g2_i1 | 92.62 | 1.00E-58 | 201 |
| AT2G25930.1 | c99108_g3_i1 | 75.28 | 0 | 1592 |
| AT5G67180.1 | c99108_g3_i2 | 75.11 | 0 | 1588 |
| AT5G67180.1 | c99108_g3_i3 | 75.19 | 0 | 1591 |
| AT4G20370.1 | c99108_g3_i4 | 75.98 | 2.00E-93 | 316 |
| AT1G29160.1 | c61713_g1_i1 | 90.14 | 2.00E-38 | 135 |
| AT3G11540.1 | c98000_g4_i1 | 71.21 | 3.00E-25 | 104 |
| AT1G78300.1 | c125128_g1_i1 | 88.46 | 9.00E-58 | 186 |
| AT1G78300.1 | c145727_g1_i1 | 68.22 | 2.00E-56 | 182 |
| AT1G78300.1 | c50193_g1_i1 | 89.71 | 7.00E-37 | 129 |
| AT1G78300.1 | c50193_g2_i1 | 89.55 | 2.00E-34 | 124 |
| AT1G78300.1 | c61245_g1_i1 | 72.5 | 2.00E-121 | 359 |
| AT1G78300.1 | c85043_g5_i1 | 72.5 | 2.00E-123 | 358 |
| AT5G08330.1 | c91613_g2_i1 | 90.34 | 8.00E-153 | 441 |
| AT5G65430.3 | c91613_g4_i2 | 84.81 | 2.00E-146 | 422 |
| AT2G18915.2 | c93687_g2_i1 | 90.34 | 3.00E-155 | 441 |
| AT1G26310.1 | c93687_g2_i3 | 90.76 | 4.00E-155 | 441 |
| AT5G38480.1 | c110705_g1_i1 | 72.93 | 1.00E-58 | 188 |
| AT5G38480.1 | c123694_g1_i1 | 78.95 | 2.00E-23 | 94.7 |
| AT5G38480.1 | c52728_g1_i1 | 70.65 | 2.00E-95 | 285 |
| AT3G02520.1 | c91613_g1_i1 | 85.99 | 4.00E-128 | 369 |
| AT3G02520.1 | c91613_g2_i2 | 82.47 | 4.00E-149 | 431 |
| AT5G38480.1 | c91613_g3_i1 | 69.41 | 1.00E-119 | 354 |
| AT3G02520.1 | c91613_g4_i1 | 84.06 | 8.00E-151 | 434 |
| AT5G38480.1 | c93155_g1_i2 | 69.57 | 9.00E-64 | 218 |
| AT5G38480.1 | c93155_g1_i3 | 69.57 | 6.00E-64 | 217 |
| AT5G38480.1 | c93155_g2_i1 | 71.68 | 1.00E-49 | 168 |
| AT4G08920.1 | c93155_g2_i2 | 71.32 | 1.00E-123 | 368 |
| AT5G38480.1 | c93687_g2_i2 | 82.29 | 2.00E-116 | 338 |
| AT3G48430.1 | c98000_g4_i2 | 67.63 | 6.00E-66 | 208 |
| AT4G02020.1 | c111484_g1_i1 | 80.68 | 1.00E-44 | 151 |
| AT2G34140.1 | c68750_g1_i1 | 89.45 | 4.00E-164 | 462 |
| AT1G04400.2 | c70972_g1_i1 | 67.69 | 5.00E-87 | 265 |
| AT2G23070.1 | c76055_g1_i1 | 68.95 | 6.00E-116 | 344 |
| AT4G18130.1 | c82989_g1_i1 | 89.45 | 9.00E-163 | 462 |
| AT5G65430.3 | c29912_g1_i1 | 87.85 | 2.00E-63 | 199 |
| AT5G65430.3 | c38005_g1_i1 | 80.6 | 1.00E-31 | 116 |
| AT2G23070.1 | c64358_g1_i1 | 87.72 | 2.00E-67 | 209 |
| AT1G17455.1 | c72484_g1_i1 | 94.07 | 8.00E-75 | 234 |
| AT4G22950.1 | c74368_g1_i1 | 94.07 | 2.00E-76 | 234 |
| AT5G65430.3 | c77056_g1_i1 | 91.51 | 2.00E-63 | 205 |
| AT5G65430.3 | c77056_g2_i1 | 90.57 | 3.00E-65 | 204 |
| AT5G65430.3 | c91613_g5_i1 | 85.83 | 9.00E-72 | 221 |
| AT5G65430.3 | c93687_g3_i1 | 87.67 | 5.00E-38 | 133 |
| AT2G22540.1 | c60457_g1_i1 | 66.67 | 4.00E-51 | 169 |
| AT5G24470.1 | c81197_g1_i1 | 89.69 | 9.00E-56 | 185 |
| AT3G20740.1 | c48600_g1_i1 | 90.82 | 9.00E-58 | 181 |
| AT1G78300.1 | c68387_g1_i2 | 84.31 | 3.00E-59 | 190 |
| AT4G14540.1 | c87227_g1_i1 | 86.67 | 5.00E-27 | 102 |
| AT4G14540.1 | c87227_g1_i2 | 89.29 | 8.00E-43 | 149 |
| AT4G14540.1 | c87227_g1_i3 | 89.29 | 4.00E-43 | 149 |
| AT4G14540.1 | c90406_g1_i2 | 76.58 | 4.00E-51 | 176 |
| AT2G22540.1 | c98146_g5_i1 | 90.27 | 1.00E-66 | 209 |
| AT5G67180.1 | c98146_g5_i2 | 90.27 | 5.00E-67 | 209 |
| AT4G11880.1 | c72831_g2_i2 | 87.34 | 1.00E-39 | 136 |
| AT4G22950.1 | c81528_g1_i1 | 70.99 | 2.00E-75 | 234 |
| AT1G04400.2 | c81528_g2_i1 | 95.89 | 1.00E-45 | 149 |
| AT1G08970.4 | c105577_g1_i1 | 77.33 | 4.00E-36 | 127 |
| AT1G08970.4 | c106937_g1_i1 | 97.59 | 4.00E-53 | 171 |
| AT5G24930.1 | c78473_g1_i2 | 90.09 | 2.00E-66 | 216 |
| AT4G18130.1 | c95551_g1_i1 | 92.93 | 6.00E-61 | 194 |
| AT4G18130.1 | c95551_g2_i1 | 90.27 | 9.00E-66 | 222 |
| AT5G60910.1 | c95551_g2_i3 | 90.27 | 1.00E-68 | 222 |
| AT5G63470.1 | c117401_g1_i1 | 95 | 4.00E-20 | 84.7 |
| AT5G63470.1 | c78992_g1_i1 | 93.18 | 7.00E-54 | 176 |
| AT5G63470.1 | c78992_g2_i1 | 96.74 | 6.00E-51 | 168 |
| AT5G24930.1 | c78992_g3_i1 | 76.51 | 5.00E-76 | 238 |
| AT2G23380.1 | c78992_g4_i1 | 80 | 1.00E-76 | 240 |
| AT1G29160.1 | c84395_g3_i1 | 65.19 | 3.00E-63 | 209 |
| AT5G04240.1 | c84395_g3_i3 | 65.19 | 5.00E-64 | 209 |
| AT1G08970.4 | c120736_g1_i1 | 85.29 | 3.00E-29 | 116 |
| tr\|Q9LEI4\|Q9LEI4_HORVU | c55380_g1_i1 | 69.64 | 2E-21 | 88.2 |
| tr\|Q9LEI4\|Q9LEI4_HORVU | c73602_g1_i1 | 40.85 | 3E-30 | 116 |
| tr\|Q9LEI4\|Q9LEI4_HORVU | c80388_g1_i1 | 40.45 | 8E-31 | 120 |
| tr\|Q9LEI4\|Q9LEI4_HORVU | c83301_g1_i1 | 35.94 | 2E-21 | 92 |
| tr\|Q9LEI4\|Q9LEI4_HORVU | c87555_g1_i1 | 40.7 | 9E-27 | 109 |
| tr\|Q9LEI4\|Q9LEI4_HORVU | c89673_g5_i2 | 60 | 1E-20 | 89.4 |
| tr\|Q9LEI4\|Q9LEI4_HORVU | c94119_g4_i1 | 37.5 | 1E-34 | 129 |
| AT4G02560.1 | c98104_g1_i1 | 63.19 | 1.00E-56 | 202 |
| AT4G02560.1 | c98104_g1_i2 | 63.19 | 2.00E-56 | 201 |
| AT4G02560.1 | c98104_g1_i3 | 63.19 | 1.00E-56 | 201 |
| AT4G02560.1 | c98104_g2_i1 | 64.29 | 4.00E-65 | 141 |
| AT1G30970.1 | c98104_g2_i2 | 59.41 | 3.00E-77 | 277 |
| AT2G22540.1 | c98104_g2_i3 | 59.41 | 9.00E-79 | 279 |
| AT1G13260.1 | c98104_g2_i4 | 59.41 | 8.00E-81 | 278 |
| AT2G22540.1 | c98104_g2_i5 | 59.41 | 3.00E-77 | 277 |
| AT4G02560.1 | c98104_g3_i1 | 81.91 | 1.00E-36 | 137 |
| AT4G08920.1 | c96427_g2_i1 | 89.81 | 7.00E-102 | 316 |
| AT2G46790.1 | c96427_g2_i2 | 89.81 | 3.00E-98 | 313 |
| AT5G17690.1 | c27843_g1_i1 | 56.67 | 1.00E-25 | 104 |
| AT5G17690.1 | c87281_g1_i1 | 61 | 2.00E-28 | 117 |
| AT1G25540.1 | c87281_g1_i2 | 70.65 | 9.00E-31 | 124 |
| AT5G38480.1 | c90643_g1_i1 | 49.68 | 2.00E-33 | 135 |
| AT5G38480.1 | c90643_g1_i2 | 49.68 | 2.00E-33 | 134 |
| AT5G38480.1 | c90643_g1_i3 | 49.68 | 1.00E-33 | 134 |
| AT1G01060.1 | c58074_g2_i1 | 43.2 | 5.00E-55 | 204 |
| AT1G01060.1 | c58074_g2_i3 | 43.2 | 5.00E-55 | 204 |
| AT1G01060.1 | c84815_g6_i1 | 57.14 | 2.00E-21 | 93.6 |
| AT1G01060.1 | c84815_g8_i1 | 60 | 1.00E-20 | 97.8 |
| AT1G01060.1 | c85679_g2_i2 | 64.43 | 1.00E-36 | 144 |
| AT1G01060.1 | c92088_g3_i3 | 57.14 | 4.00E-22 | 101 |
| AT1G01060.1 | c92088_g3_i5 | 57.14 | 4.00E-22 | 101 |
| AT1G01060.1 | c96879_g4_i1 | 57.14 | 4.00E-21 | 99.8 |
| AT1G01060.1 | c99092_g1_i1 | 41.18 | 8.00E-101 | 339 |
| AT2G28550.3 | c99092_g1_i2 | 41.18 | 2.00E-101 | 339 |
| AT1G01060.1 | c99092_g1_i3 | 41.18 | 8.00E-101 | 339 |
| AT1G01060.1 | c99092_g1_i4 | 39.08 | 1.00E-48 | 187 |
| AT2G28550.3 | c99092_g1_i5 | 41.18 | 3.00E-101 | 339 |
| AT1G01060.1 | c99092_g1_i6 | 41.18 | 6.00E-101 | 339 |
| AT5G60120.2 | c99092_g1_i7 | 41.18 | 2.00E-101 | 339 |
| AT3G02380.1 | c99092_g1_i8 | 41.18 | 3.00E-101 | 339 |
| AT1G01060.1 | c99092_g2_i1 | 43.37 | 1.00E-35 | 140 |
| AT5G13480.2 | c127840_g1_i1 | 66.47 | 2.00E-71 | 233 |
| AT2G23070.1 | c56739_g1_i1 | 69.5 | 2.00E-61 | 206 |
| AT2G18915.2 | c88534_g2_i1 | 30.57 | 2.00E-24 | 111 |
| AT3G10390.1 | c88534_g2_i2 | 27.02 | 5.00E-32 | 133 |
| AT2G18915.2 | c88534_g2_i3 | 30.57 | 2.00E-24 | 111 |
| AT5G10140.1 | c56831_g1_i1 | 82.28 | 1.00E-38 | 137 |
| AT3G04610.1 | c72415_g1_i1 | 56.03 | 4.00E-61 | 208 |
| AT3G46640.3 | c92222_g4_i1 | 56.1 | 3.00E-23 | 98.2 |
| AT3G19290.3 | c92489_g5_i1 | 61.33 | 3.00E-69 | 226 |
| AT4G16280.2 | c96650_g1_i1 | 73.2 | 3.00E-35 | 138 |
| AT3G46640.3 | c96650_g1_i2 | 73.2 | 3.00E-35 | 138 |
| AT1G25540.1 | c95303_g7_i2 | 43.04 | 8.00E-29 | 112 |
| AT5G38480.1 | c95303_g7_i4 | 48.34 | 4.00E-33 | 129 |
| AT1G25540.1 | c95303_g7_i5 | 61.02 | 1.00E-21 | 82.4 |
| AT1G09570.1 | c95303_g7_i6 | 43.04 | 2.00E-27 | 112 |
| AT5G23260.2 | c91178_g1_i1 | 37.75 | 2.00E-21 | 94.4 |
| AT5G58230.1 | c134658_g1_i1 | 65.82 | 1.00E-28 | 110 |
| AT5G58230.1 | c14192_g1_i1 | 87.18 | 1.00E-41 | 147 |
| AT5G58230.1 | c29905_g1_i1 | 90.77 | 3.00E-73 | 231 |
| AT5G58230.1 | c49531_g1_i1 | 84.88 | 2.00E-46 | 159 |
| AT3G53340.1 | c55622_g1_i1 | 55.25 | 8.00E-111 | 333 |
| AT5G58230.1 | c57444_g1_i1 | 85.96 | 4.00E-58 | 192 |
| AT1G69120.1 | c81655_g1_i1 | 59.1 | 4.00E-166 | 481 |
| AT5G61850.1 | c89972_g1_i1 | 88.92 | 0 | 600 |
| AT1G22770.1 | c89972_g2_i1 | 92.06 | 2.00E-166 | 475 |
| AT5G58230.1 | c92476_g3_i1 | 30.63 | 3.00E-31 | 128 |
| LOC_Os04g37920.1 | c70927_g1_i1 | 65.69 | 3E-107 | 332 |
| LOC_Os04g37920.1 | c70927_g1_i2 | 69.06 | 4E-106 | 328 |
| LOC_Os10g32600.1 | c126018_g1_i1 | 40.79 | 4E-29 | 109 |
| LOC_Os01g72220.1 | c93616_g2_i1 | 82.67 | 0 | 590 |
| LOC_Os01g51610.1 | c25733_g2_i1 | 57.84 | 7E-33 | 127 |
| LOC_Os01g51610.1 | c29411_g1_i1 | 57.33 | 3E-24 | 98.6 |
| LOC_Os01g51610.1 | c97066_g3_i1 | 39.62 | 7E-20 | 94.4 |
| LOC_Os01g51610.1 | c97066_g3_i2 | 39.62 | 6E-20 | 94.4 |
| LOC_Os03g54084.1 | c68853_g1_i1 | 43.73 | 4E-91 | 302 |
| LOC_Os05g47650.1 | c83982_g1_i1 | 51.29 | 8E-98 | 302 |
| AT5G42790.1 | c111343_g1_i1 | 39.26 | 6.00E-21 | 89.4 |
| AT5G42790.1 | c70727_g1_i1 | 43.75 | 9.00E-26 | 103 |
| AT5G42790.1 | c73146_g1_i1 | 39.18 | 5.00E-34 | 132 |
| AT5G42790.1 | c76027_g1_i1 | 35.29 | 2.00E-38 | 143 |
| AT3G57390.1 | c78703_g1_i1 | 85.71 | 2.00E-149 | 433 |
| AT5G23260.2 | c87709_g2_i1 | 38.65 | 7.00E-43 | 155 |
| AT3G10390.1 | c87709_g2_i2 | 46.33 | 1.00E-42 | 151 |
| AT3G10390.1 | c87709_g2_i3 | 46.33 | 1.00E-42 | 151 |
| AT1G47250.1 | c129114_g1_i1 | 64.44 | 4.00E-20 | 65.5 |
| AT1G47250.1 | c25465_g1_i1 | 36.64 | 3.00E-24 | 99 |
| AT2G23070.1 | c78703_g2_i1 | 90.08 | 5.00E-75 | 231 |
| AT1G47250.1 | c80165_g1_i1 | 35.16 | 8.00E-34 | 130 |
| AT5G35840.1 | c87709_g1_i2 | 45.3 | 4.00E-44 | 155 |
| AT2G18790.1 | c87872_g1_i1 | 38.35 | 5.00E-41 | 150 |
| AT3G04610.1 | c87872_g1_i2 | 38.35 | 4.00E-41 | 150 |
| AT1G47250.1 | c87872_g1_i3 | 38.35 | 5.00E-41 | 150 |
| AT1G47250.1 | c87872_g1_i4 | 38.35 | 5.00E-41 | 150 |
| AT1G47250.1 | c89728_g3_i4 | 36.32 | 5.00E-37 | 138 |
| AT3G57390.1 | c105476_g1_i1 | 63.5 | 9.00E-46 | 160 |
| AT4G26000.1 | c86791_g1_i1 | 44.62 | 1.00E-20 | 91.7 |
| AT5G61850.1 | c86791_g2_i1 | 57.33 | 3.00E-158 | 474 |
| AT2G46790.1 | c90763_g1_i1 | 28.11 | 6.00E-26 | 114 |
| AT4G26000.1 | c90763_g1_i2 | 27.8 | 4.00E-20 | 88.2 |
| AT5G42790.1 | c94848_g2_i2 | 33.92 | 4.00E-21 | 98.6 |
| AT1G49720.2 | c129256_g1_i1 | 88.35 | 7.00E-59 | 199 |
| AT3G30260.1 | c92488_g1_i1 | 63.88 | 0 | 782 |
| AT1G08970.4 | c92488_g2_i1 | 58.13 | 3.00E-62 | 212 |
| AT5G24470.1 | c141609_g1_i1 | 58.42 | 1.00E-32 | 127 |
| AT5G61380.1 | c93518_g3_i1 | 78.26 | 0 | 728 |
| AT2G18790.1 | c101617_g1_i1 | 75.34 | 3.00E-30 | 119 |
| AT2G18790.1 | c103567_g1_i1 | 69.01 | 5.00E-31 | 121 |
| AT3G11540.1 | c117126_g1_i1 | 87.38 | 3.00E-55 | 191 |
| AT3G02380.1 | c12922_g1_i1 | 64.57 | 4.00E-50 | 177 |
| AT2G18790.1 | c12922_g1_i2 | 54.12 | 6.00E-22 | 96.3 |
| AT2G18790.1 | c95474_g2_i1 | 80.6 | 5.00E-28 | 121 |
| AT3G33520.1 | c95474_g2_i2 | 78.01 | 0 | 1867 |
| AT2G18790.1 | c95474_g3_i1 | 84.51 | 3.00E-39 | 144 |
| AT5G35840.1 | c95474_g4_i1 | 85.71 | 1.00E-57 | 198 |
| AT5G35840.1 | c107120_g1_i1 | 58.57 | 3.00E-24 | 102 |
| AT1G08970.4 | c95421_g1_i1 | 63.83 | 0 | 1439 |
| AT2G18790.1 | c95421_g1_i2 | 63.83 | 0 | 1439 |
| AT2G18790.1 | c95421_g1_i4 | 63.83 | 0 | 1439 |
| AT2G18790.1 | c95421_g1_i5 | 63.83 | 0 | 1439 |
| AT5G35840.1 | c95421_g1_i6 | 63.83 | 0 | 1439 |
| AT5G35840.1 | c95421_g1_i7 | 63.83 | 0 | 1439 |
| AT3G46640.3 | c112901_g1_i1 | 71.82 | 3.00E-51 | 180 |
| AT1G13260.1 | c93518_g1_i1 | 46.08 | 0 | 986 |
| AT2G46790.1 | c96432_g1_i1 | 59.09 | 4.00E-51 | 181 |
| AT4G14540.1 | c96432_g2_i1 | 62.89 | 0 | 1449 |
| AT4G18130.1 | c96432_g2_i2 | 78.57 | 2.00E-28 | 119 |
| AT3G12810.1 | c100164_g2_i2 | 43.77 | 6.00E-71 | 267 |
| AT5G61380.1 | c100311_g2_i1 | 60.81 | 0 | 2037 |
| AT3G12810.1 | c109091_g1_i1 | 50.79 | 1.00E-33 | 132 |
| AT3G12810.1 | c109531_g1_i1 | 51.35 | 8.00E-27 | 112 |
| AT3G21320.1 | c134373_g1_i1 | 36.36 | 6.00E-22 | 101 |
| AT3G12810.1 | c137164_g1_i1 | 42.24 | 5.00E-21 | 95.1 |
| AT3G12810.1 | c23807_g1_i1 | 48.11 | 1.00E-24 | 106 |
| AT3G12810.1 | c58680_g1_i1 | 68.97 | 9.00E-36 | 137 |
| AT3G12810.1 | c65546_g1_i1 | 66.06 | 9.00E-46 | 169 |
| AT3G12810.1 | c79319_g4_i1 | 60.98 | 6.00E-28 | 115 |
| AT3G12810.1 | c83396_g2_i1 | 83.1 | 2.00E-29 | 122 |
| AT3G12810.1 | c83396_g2_i2 | 83.1 | 6.00E-30 | 122 |
| AT3G12810.1 | c83396_g9_i1 | 64.44 | 1.00E-51 | 185 |
| AT3G12810.1 | c84580_g2_i2 | 56.21 | 2.00E-45 | 181 |
| AT3G12810.1 | c85564_g2_i1 | 40.06 | 4.00E-68 | 246 |
| AT3G12810.1 | c86085_g3_i2 | 45.83 | 6.00E-25 | 106 |
| AT3G12810.1 | c86085_g3_i3 | 46.88 | 7.00E-25 | 110 |
| AT3G12810.1 | c86748_g2_i1 | 52.03 | 4.00E-31 | 128 |
| AT3G12810.1 | c89213_g2_i1 | 38.76 | 2.00E-22 | 107 |
| AT3G12810.1 | c89213_g2_i2 | 38.76 | 2.00E-22 | 107 |
| AT3G12810.1 | c89266_g3_i1 | 30.83 | 1.00E-20 | 102 |
| AT3G12810.1 | c89266_g3_i10 | 30.83 | 4.00E-20 | 100 |
| AT3G12810.1 | c89266_g3_i12 | 30.83 | 3.00E-20 | 100 |
| AT3G12810.1 | c89266_g3_i13 | 30.83 | 3.00E-20 | 100 |
| AT3G12810.1 | c89266_g3_i14 | 30.83 | 4.00E-20 | 100 |
| AT3G12810.1 | c89266_g3_i15 | 30.83 | 4.00E-20 | 100 |
| AT3G12810.1 | c89266_g3_i7 | 30.83 | 4.00E-20 | 100 |
| AT3G12810.1 | c89266_g3_i8 | 30.83 | 4.00E-20 | 100 |
| AT3G12810.1 | c89266_g3_i9 | 30.83 | 4.00E-20 | 100 |
| AT3G12810.1 | c89563_g1_i1 | 40.32 | 1.00E-21 | 105 |
| AT3G12810.1 | c92275_g1_i1 | 31.55 | 3.00E-31 | 136 |
| AT3G12810.1 | c92275_g1_i2 | 31.55 | 3.00E-31 | 136 |
| AT3G12810.1 | c92902_g3_i1 | 33.02 | 7.00E-41 | 167 |
| AT3G12810.1 | c92902_g3_i5 | 33.02 | 7.00E-41 | 167 |
| AT3G12810.1 | c92902_g3_i6 | 46.3 | 7.00E-34 | 145 |
| AT3G12810.1 | c92902_g3_i7 | 46.3 | 5.00E-34 | 145 |
| AT3G12810.1 | c92902_g3_i9 | 33.02 | 8.00E-41 | 167 |
| AT3G12810.1 | c94411_g1_i1 | 32.64 | 6.00E-41 | 169 |
| AT3G12810.1 | c94411_g1_i2 | 32.64 | 6.00E-41 | 169 |
| AT3G12810.1 | c95473_g3_i1 | 29.79 | 2.00E-20 | 101 |
| AT3G12810.1 | c95473_g3_i2 | 29.79 | 2.00E-20 | 101 |
| AT3G12810.1 | c95823_g6_i1 | 38.16 | 1.00E-47 | 191 |
| AT3G12810.1 | c95823_g6_i2 | 38.16 | 1.00E-47 | 191 |
| AT3G12810.1 | c95823_g6_i3 | 38.16 | 1.00E-47 | 191 |
| AT3G12810.1 | c95932_g1_i1 | 27.76 | 8.00E-21 | 102 |
| AT3G12810.1 | c95946_g2_i1 | 38.87 | 1.00E-53 | 211 |
| AT3G12810.1 | c95946_g2_i2 | 38.87 | 1.00E-53 | 211 |
| AT3G12810.1 | c95946_g2_i3 | 37.89 | 7.00E-52 | 204 |
| AT4G35900.1 | c96115_g2_i1 | 47 | 8.00E-87 | 318 |
| AT5G24470.1 | c96115_g2_i2 | 47 | 8.00E-87 | 318 |
| AT3G12810.1 | c96482_g1_i1 | 39.86 | 2.00E-51 | 201 |
| AT3G12810.1 | c96482_g1_i2 | 39.86 | 2.00E-49 | 195 |
| AT3G12810.1 | c96505_g1_i1 | 32.42 | 2.00E-36 | 153 |
| AT3G12810.1 | c96625_g2_i2 | 33.93 | 4.00E-22 | 107 |
| AT3G12810.1 | c97515_g2_i1 | 39.88 | 1.00E-58 | 227 |
| AT3G12810.1 | c97515_g2_i2 | 41.19 | 1.00E-59 | 231 |
| AT3G12810.1 | c97601_g1_i2 | 30.64 | 5.00E-27 | 123 |
| AT3G12810.1 | c97601_g1_i3 | 30.64 | 5.00E-27 | 123 |
| AT4G35900.1 | c98691_g1_i1 | 42.32 | 2.00E-72 | 264 |
| AT3G12810.1 | c98691_g1_i2 | 42.32 | 2.00E-72 | 264 |
| AT3G12810.1 | c98691_g2_i1 | 43.73 | 6.00E-70 | 261 |
| AT3G12810.1 | c98765_g4_i2 | 37.79 | 8.00E-56 | 218 |
| AT3G12810.1 | c98765_g4_i3 | 37.79 | 7.00E-56 | 218 |
| AT3G12810.1 | c98765_g4_i4 | 37.79 | 1.00E-56 | 214 |
| AT3G12810.1 | c98765_g4_i5 | 37.79 | 7.00E-56 | 218 |
| AT3G12810.1 | c99276_g1_i1 | 39.75 | 2.00E-66 | 248 |
| AT3G12810.1 | c99276_g1_i2 | 39.75 | 2.00E-66 | 248 |
| AT3G12810.1 | c99276_g1_i3 | 39.75 | 6.00E-66 | 246 |
| AT3G12810.1 | c99276_g1_i4 | 39.75 | 6.00E-66 | 246 |
| AT3G12810.1 | c99276_g3_i1 | 43.24 | 8.00E-30 | 121 |
| AT3G12810.1 | c99431_g4_i1 | 38.46 | 1.00E-24 | 115 |
| AT3G12810.1 | c99431_g4_i2 | 38.46 | 1.00E-24 | 115 |
| AT3G12810.1 | c99431_g6_i1 | 38.46 | 3.00E-23 | 108 |
| AT3G12810.1 | c99506_g3_i1 | 31.96 | 1.00E-42 | 174 |
| AT3G12810.1 | c99506_g3_i10 | 31.96 | 4.00E-43 | 176 |
| AT3G12810.1 | c99506_g3_i2 | 31.96 | 5.00E-43 | 176 |
| AT3G12810.1 | c99506_g3_i3 | 31.96 | 3.00E-43 | 176 |
| AT3G12810.1 | c99506_g3_i4 | 31.96 | 3.00E-43 | 176 |
| AT3G12810.1 | c99506_g3_i6 | 31.96 | 4.00E-43 | 176 |
| AT3G12810.1 | c99506_g3_i7 | 31.96 | 5.00E-43 | 176 |
| AT3G12810.1 | c99506_g3_i8 | 31.96 | 4.00E-43 | 176 |
| AT3G12810.1 | c99993_g3_i1 | 41.28 | 1.00E-34 | 147 |
| AT3G12810.1 | c99993_g3_i2 | 29.97 | 6.00E-35 | 148 |
| AT3G12810.1 | c99993_g3_i3 | 30.64 | 1.00E-35 | 150 |
| AT3G12810.1 | c99993_g3_i4 | 42.07 | 8.00E-34 | 144 |
| AT3G12810.1 | c99993_g3_i5 | 31.34 | 2.00E-29 | 127 |
| AT3G12810.1 | c99993_g4_i1 | 46 | 3.00E-23 | 101 |
| AT5G02810.1 | c80738_g1_i1 | 58.14 | 2.00E-26 | 110 |
| AT5G02810.1 | c80738_g1_i2 | 42.57 | 2.00E-41 | 157 |
| AT5G02810.1 | c85628_g4_i1 | 36.17 | 8.00E-24 | 109 |
| AT5G02810.1 | c85628_g4_i2 | 41.14 | 3.00E-39 | 156 |
| AT1G18100.1 | c98035_g1_i1 | 48.62 | 7.00E-99 | 336 |
| AT3G11540.1 | c98035_g1_i2 | 63.08 | 2.00E-82 | 288 |
| AT1G30970.1 | c98035_g1_i3 | 44.34 | 2.00E-87 | 204 |
| AT1G30970.1 | c98035_g2_i1 | 62.55 | 2.00E-72 | 253 |
| AT5G02810.1 | c98035_g2_i2 | 62.55 | 3.00E-72 | 253 |
| AT5G51230.1 | c5265_g1_i1 | 76.67 | 1.00E-39 | 143 |
| AT5G24470.1 | c106579_g1_i1 | 85.32 | 2.00E-59 | 199 |
| AT5G60910.1 | c51756_g1_i1 | 75.27 | 3.00E-40 | 146 |
| AT5G24470.1 | c56212_g1_i1 | 75.49 | 2.00E-47 | 167 |
| AT5G24470.1 | c78668_g1_i1 | 33.51 | 2.00E-23 | 107 |
| AT5G24470.1 | c92222_g2_i1 | 32.61 | 3.00E-21 | 100 |
| AT5G24470.1 | c92222_g2_i2 | 32.61 | 4.00E-21 | 100 |
| AT1G08970.4 | c92704_g6_i1 | 39.48 | 1.00E-116 | 374 |
| AT4G30200.2 | c92704_g6_i2 | 39.76 | 2.00E-116 | 374 |
| AT4G11110.1 | c93974_g2_i1 | 77.37 | 9.00E-70 | 228 |
| AT5G24470.1 | c93974_g3_i1 | 33.83 | 5.00E-57 | 208 |
| AT3G56850.1 | c96565_g2_i1 | 70.11 | 2.00E-67 | 241 |
| AT5G24470.1 | c96565_g2_i2 | 70.11 | 3.00E-67 | 241 |
| AT2G46790.1 | c91063_g2_i1 | 40.98 | 5.00E-22 | 101 |
| AT2G46830.1 | c92222_g3_i1 | 40.65 | 3.00E-22 | 101 |
| AT2G46790.1 | c96565_g1_i1 | 93.18 | 1.00E-20 | 90.5 |
| AT5G61380.1 | c98813_g2_i1 | 36.53 | 3.00E-25 | 111 |
| AT5G61380.1 | c98813_g3_i1 | 36.53 | 1.00E-26 | 110 |
| AT4G34530.1 | c98813_g3_i2 | 42.5 | 5.00E-26 | 105 |
| AT1G13260.1 | c115816_g1_i1 | 71.76 | 3.00E-34 | 126 |
| AT1G13260.1 | c61782_g1_i1 | 50.38 | 3.00E-33 | 124 |
| AT2G32950.1 | c83982_g1_i2 | 59.77 | 6.00E-130 | 385 |
| AT5G23150.1 | c86857_g1_i1 | 64.22 | 3.00E-133 | 390 |
| AT5G58230.1 | c94181_g3_i1 | 70.75 | 1.00E-43 | 154 |
| AT5G58230.1 | c94181_g4_i2 | 68.97 | 8.00E-43 | 160 |
| AT1G13260.1 | c94181_g4_i3 | 68.97 | 1.00E-42 | 160 |
| AT1G13260.1 | c94181_g4_i6 | 68.97 | 2.00E-42 | 160 |
| AT5G24930.1 | c86857_g1_i2 | 63.86 | 4.00E-25 | 100 |
| AT1G77300.1 | c99391_g1_i2 | 37.87 | 6.00E-35 | 138 |
| tr\|C5X1F3\|C5X1F3_SORBI | c103219_g1_i1 | 73.55 | 1E-54 | 181 |
| tr\|C5X1F3\|C5X1F3_SORBI | c123769_g1_i1 | 76.67 | 2E-28 | 110 |
| tr\|C5X1F3\|C5X1F3_SORBI | c14388_g1_i1 | 80 | 9E-29 | 111 |
| tr\|C5X1F3\|C5X1F3_SORBI | c55945_g1_i1 | 78.75 | 5E-43 | 152 |
| tr\|C5X1F3\|C5X1F3_SORBI | c79414_g2_i1 | 71.52 | 2E-76 | 241 |
| tr\|C5X1F3\|C5X1F3_SORBI | c80203_g1_i1 | 54.76 | 2E-20 | 87.8 |
| tr\|C5X1F3\|C5X1F3_SORBI | c80203_g2_i2 | 57.8 | 9E-63 | 217 |
| tr\|C5X1F3\|C5X1F3_SORBI | c81138_g1_i1 | 34.19 | 4E-21 | 97.1 |
| tr\|C5X1F3\|C5X1F3_SORBI | c81138_g1_i2 | 34.19 | 4E-21 | 97.1 |
| tr\|C5X1F3\|C5X1F3_SORBI | c82411_g2_i1 | 55.95 | 2E-57 | 195 |
| tr\|C5X1F3\|C5X1F3_SORBI | c82411_g2_i2 | 66.67 | 2E-79 | 253 |
| tr\|C5X1F3\|C5X1F3_SORBI | c82523_g1_i2 | 48.72 | 8E-37 | 135 |
| tr\|C5X1F3\|C5X1F3_SORBI | c82523_g2_i1 | 68.55 | 2E-55 | 189 |
| tr\|C5X1F3\|C5X1F3_SORBI | c82523_g2_i2 | 68.55 | 4E-56 | 188 |
| tr\|C5X1F3\|C5X1F3_SORBI | c82523_g3_i1 | 79.63 | 6E-25 | 108 |
| tr\|C5X1F3\|C5X1F3_SORBI | c87244_g1_i1 | 34.38 | 2E-21 | 98.6 |
| tr\|C5X1F3\|C5X1F3_SORBI | c87244_g1_i2 | 34.38 | 2E-21 | 98.6 |
| tr\|C5X1F3\|C5X1F3_SORBI | c92595_g6_i1 | 74.14 | 7E-86 | 283 |
| tr\|C5X1F3\|C5X1F3_SORBI | c92595_g6_i2 | 75.29 | 1E-88 | 280 |
| tr\|C5X1F3\|C5X1F3_SORBI | c93923_g1_i1 | 33.95 | 3E-21 | 98.2 |
| tr\|C5X1F3\|C5X1F3_SORBI | c93923_g1_i2 | 33.95 | 3E-21 | 98.2 |
| tr\|C5X1F3\|C5X1F3_SORBI | c93923_g1_i3 | 33.95 | 3E-21 | 98.2 |
| tr\|C5X1F3\|C5X1F3_SORBI | c94665_g2_i1 | 68.24 | 1E-79 | 271 |
| tr\|C5X1F3\|C5X1F3_SORBI | c94665_g2_i2 | 68.24 | 5E-81 | 271 |
| tr\|C5X1F3\|C5X1F3_SORBI | c95484_g2_i1 | 81.36 | 2E-31 | 120 |
| tr\|C5X1F3\|C5X1F3_SORBI | c95484_g3_i1 | 80 | 9E-27 | 114 |
| tr\|C5X1F3\|C5X1F3_SORBI | c95484_g4_i1 | 67.97 | 4E-56 | 189 |
| tr\|C5X1F3\|C5X1F3_SORBI | c96507_g1_i1 | 82.35 | 9E-48 | 167 |
| tr\|C5X1F3\|C5X1F3_SORBI | c96507_g4_i1 | 70.45 | 9E-85 | 281 |
| tr\|C5X1F3\|C5X1F3_SORBI | c96507_g4_i2 | 70.45 | 1E-84 | 281 |
| tr\|C5X1F3\|C5X1F3_SORBI | c96507_g4_i3 | 72.67 | 9E-79 | 265 |
| tr\|C5X1F3\|C5X1F3_SORBI | c97165_g2_i1 | 73.53 | 5E-83 | 277 |
| tr\|C5X1F3\|C5X1F3_SORBI | c97165_g2_i2 | 73.53 | 6E-81 | 273 |
| tr\|C5X1F3\|C5X1F3_SORBI | c97165_g2_i3 | 73.53 | 5E-83 | 277 |
| tr\|C5X1F3\|C5X1F3_SORBI | c9945_g1_i1 | 81.25 | 2E-44 | 154 |
| AT3G21320.1 | c86010_g1_i3 | 75.44 | 9.00E-55 | 175 |
| AT5G51230.1 | c86010_g2_i1 | 75.44 | 2.00E-54 | 175 |
| AT2G45660.1 | c88293_g2_i2 | 78.46 | 2.00E-31 | 114 |
| AT2G45660.1 | c88293_g3_i1 | 56.19 | 9.00E-35 | 124 |
| AT5G44080.1 | c88293_g4_i1 | 59.79 | 6.00E-35 | 126 |
| AT2G45660.1 | c88293_g4_i4 | 59.79 | 8.00E-34 | 125 |
| AT2G45660.1 | c88293_g4_i5 | 59.79 | 3.00E-34 | 126 |
| AT5G13480.2 | c89673_g4_i2 | 66.36 | 3.00E-43 | 145 |
| AT2G45660.1 | c89673_g5_i1 | 79.41 | 2.00E-31 | 117 |
| AT2G45660.1 | c90323_g2_i1 | 52.25 | 2.00E-31 | 115 |
| AT2G45660.1 | c94107_g3_i3 | 86 | 1.00E-23 | 94.7 |
| AT2G27550.1 | c99509_g1_i1 | 73.91 | 1.00E-42 | 155 |
| AT2G27550.1 | c99509_g2_i1 | 49.8 | 0 | 872 |
| AT4G11110.1 | c99509_g2_i2 | 69.81 | 2.00E-40 | 155 |
| AT2G27550.1 | c99509_g2_i4 | 49.4 | 0 | 864 |
| AT1G77300.1 | c99509_g2_i5 | 40.11 | 1.00E-133 | 429 |
| AT3G15354.1 | c8179_g1_i1 | 70.31 | 3.00E-25 | 104 |
| AT4G11110.1 | c97089_g2_i1 | 64 | 2.00E-56 | 194 |
| AT3G15354.1 | c97089_g3_i1 | 68.84 | 3.00E-46 | 165 |
| AT4G00650.1 | c97089_g4_i1 | 54.78 | 0 | 673 |
| AT3G15354.1 | c97089_g4_i2 | 49.67 | 9.00E-33 | 135 |
| AT3G15354.1 | c97089_g4_i3 | 49.67 | 2.00E-32 | 135 |
| AT3G15354.1 | c97089_g4_i4 | 59.16 | 0 | 958 |
| AT3G15354.1 | c97089_g4_i5 | 54.78 | 0 | 673 |
| AT4G14540.1 | c27182_g1_i1 | 51.66 | 1.00E-57 | 199 |
| AT2G46830.1 | c64106_g1_i2 | 74.04 | 1.00E-57 | 149 |
| AT2G33810.1 | c129220_g1_i1 | 66.67 | 1.00E-35 | 123 |
| AT2G33810.1 | c135760_g1_i1 | 68.33 | 1.00E-22 | 90.9 |
| AT2G33810.1 | c136351_g1_i1 | 72 | 9.00E-34 | 119 |
| AT2G33810.1 | c141441_g1_i1 | 66.28 | 7.00E-34 | 118 |
| AT2G33810.1 | c68350_g1_i1 | 64.63 | 1.00E-30 | 116 |
| AT1G17455.1 | c75109_g1_i1 | 76.92 | 5.00E-38 | 133 |
| AT4G34530.1 | c75109_g1_i2 | 76.92 | 2.00E-37 | 132 |
| AT2G33810.1 | c79187_g1_i1 | 71.62 | 4.00E-32 | 122 |
| AT2G33810.1 | c79187_g1_i2 | 62.5 | 5.00E-31 | 120 |
| AT2G33810.1 | c79890_g1_i1 | 70.89 | 7.00E-34 | 125 |
| AT2G33810.1 | c79890_g1_i2 | 70.89 | 7.00E-34 | 125 |
| AT4G09000.2 | c80807_g1_i1 | 76.92 | 2.00E-37 | 136 |
| AT2G32950.1 | c80807_g1_i2 | 76.92 | 2.00E-37 | 133 |
| AT2G33810.1 | c80807_g1_i3 | 76.92 | 2.00E-37 | 136 |
| AT2G33810.1 | c81260_g1_i2 | 58.89 | 4.00E-30 | 117 |
| AT2G33810.1 | c81320_g1_i1 | 65.22 | 7.00E-34 | 125 |
| AT2G33810.1 | c82819_g4_i3 | 56.04 | 6.00E-30 | 113 |
| AT2G33810.1 | c87494_g1_i3 | 60.81 | 3.00E-26 | 107 |
| AT2G33810.1 | c87494_g1_i5 | 60.81 | 3.00E-26 | 107 |
| AT2G33810.1 | c87494_g1_i6 | 60.81 | 3.00E-26 | 107 |
| AT2G33810.1 | c89908_g2_i1 | 71.43 | 4.00E-33 | 124 |
| AT2G33810.1 | c91703_g1_i1 | 64.63 | 6.00E-30 | 117 |
| AT2G33810.1 | c91703_g1_i3 | 64.63 | 5.00E-30 | 117 |
| AT2G33810.1 | c91703_g3_i1 | 65.22 | 1.00E-32 | 125 |
| AT2G33810.1 | c92156_g2_i1 | 66.22 | 9.00E-28 | 110 |
| AT2G33810.1 | c92156_g2_i2 | 66.22 | 1.00E-27 | 110 |
| AT2G33810.1 | c92156_g2_i3 | 66.22 | 9.00E-28 | 110 |
| AT2G33810.1 | c93310_g3_i1 | 72.97 | 1.00E-30 | 115 |
| AT3G12810.1 | c93310_g3_i2 | 69.14 | 9.00E-32 | 119 |
| AT2G33810.1 | c93310_g5_i1 | 68.92 | 7.00E-30 | 112 |
| AT2G33810.1 | c93493_g1_i1 | 63.29 | 4.00E-30 | 116 |
| AT2G33810.1 | c93493_g1_i2 | 63.29 | 3.00E-31 | 114 |
| AT2G33810.1 | c93493_g1_i3 | 63.29 | 3.00E-30 | 116 |
| AT2G33810.1 | c94853_g3_i1 | 64.56 | 9.00E-30 | 117 |
| AT2G33810.1 | c96319_g1_i1 | 69.33 | 2.00E-29 | 114 |
| AT2G33810.1 | c96319_g1_i2 | 63.86 | 4.00E-30 | 114 |
| AT2G33810.1 | c96319_g1_i3 | 69.33 | 2.00E-29 | 114 |
| AT2G33810.1 | c96319_g1_i4 | 63.86 | 5.00E-30 | 114 |
| AT2G33810.1 | c96319_g1_i5 | 69.33 | 2.00E-29 | 114 |
| AT2G33810.1 | c96319_g1_i6 | 69.33 | 2.00E-29 | 114 |
| AT2G33810.1 | c96319_g1_i7 | 69.33 | 2.00E-29 | 114 |
| AT2G33810.1 | c97164_g4_i1 | 55.41 | 1.00E-21 | 89.4 |
| AT2G33810.1 | c97164_g5_i1 | 55.41 | 2.00E-20 | 90.5 |
| AT2G33810.1 | c97164_g5_i2 | 55.41 | 3.00E-20 | 89.7 |
| AT3G11540.1 | c129215_g1_i1 | 94.32 | 7.00E-50 | 174 |
| AT3G02380.1 | c56630_g1_i1 | 89.71 | 2.00E-84 | 271 |
| AT5G60910.1 | c82061_g1_i2 | 28.1 | 4.00E-83 | 293 |
| AT4G14540.1 | c92144_g9_i1 | 84.35 | 0 | 1496 |
| AT5G58230.1 | c92144_g9_i2 | 84.23 | 0 | 1496 |
| AT4G36920.1 | c69103_g1_i1 | 69.41 | 7.00E-68 | 216 |
| AT1G47250.1 | c84333_g1_i1 | 61.2 | 5.00E-130 | 395 |
| AT1G17455.1 | c84333_g1_i2 | 60.06 | 1.00E-113 | 359 |
| AT2G22540.1 | c101570_g1_i1 | 66.06 | 2.00E-44 | 151 |
| AT2G22540.1 | c15711_g1_i1 | 58.49 | 8.00E-34 | 122 |
| AT2G22540.1 | c64449_g1_i1 | 62.96 | 5.00E-44 | 149 |
| AT2G22540.1 | c88116_g2_i1 | 54.39 | 2.00E-79 | 251 |
| AT2G13570.1 | c90289_g1_i1 | 68.46 | 4.00E-106 | 316 |
| AT4G09000.2 | c90289_g1_i2 | 68.46 | 4.00E-106 | 317 |
| AT4G09000.2 | c90289_g1_i3 | 78.21 | 7.00E-82 | 251 |
| AT1G78300.1 | c90289_g1_i4 | 68.46 | 1.00E-105 | 316 |
| AT2G22540.1 | c90289_g4_i1 | 82.95 | 1.00E-44 | 150 |
| AT2G22540.1 | c90829_g2_i1 | 55.04 | 4.00E-77 | 245 |
| AT2G22540.1 | c90829_g2_i2 | 55.04 | 9.00E-78 | 246 |
| AT2G22540.1 | c91377_g1_i11 | 50.2 | 2.00E-70 | 224 |
| AT2G22540.1 | c91377_g1_i14 | 50.2 | 2.00E-70 | 224 |
| AT2G22540.1 | c91377_g1_i2 | 59.65 | 2.00E-61 | 207 |
| AT2G22540.1 | c91377_g1_i5 | 59.65 | 2.00E-61 | 207 |
| AT2G22540.1 | c91377_g1_i7 | 50.2 | 5.00E-69 | 226 |
| AT2G22540.1 | c91377_g1_i8 | 59.65 | 1.00E-60 | 207 |
| AT2G22540.1 | c91377_g1_i9 | 59.65 | 6.00E-62 | 207 |
| AT4G02020.1 | c120777_g1_i1 | 49.46 | 1.00E-20 | 92 |
| AT4G02020.1 | c90207_g2_i1 | 42.47 | 4.00E-47 | 182 |
| AT4G02020.1 | c95674_g1_i1 | 83.33 | 5.00E-20 | 90.1 |
| AT4G18130.1 | c95674_g3_i2 | 87.19 | 5.00E-111 | 350 |
| AT3G12810.1 | c95674_g3_i3 | 57.85 | 0 | 639 |
| AT5G03840.1 | c95674_g3_i4 | 55.36 | 0 | 843 |
| AT3G12810.1 | c95674_g3_i5 | 79.41 | 1.00E-48 | 172 |
| AT2G36270.1 | c54446_g2_i1 | 72.41 | 3.00E-33 | 123 |
| AT4G11110.1 | c88536_g1_i1 | 56.38 | 1.00E-41 | 156 |
| AT4G34000.1 | c94181_g3_i2 | 54.93 | 2.00E-45 | 157 |
| AT1G53090.1 | c22179_g1_i1 | 72.29 | 1.00E-86 | 258 |
| tr\|A0SQ43\|A0SQ43_TRIMO | c93433_g3_i1 | 78.75 | 3E-37 | 137 |
| tr\|A0SQ43\|A0SQ43_TRIMO | c93433_g4_i1 | 62.41 | 1E-50 | 176 |
| AT5G61380.1 | c20504_g1_i1 | 83.58 | 8.00E-24 | 99.4 |
| AT1G47250.1 | c94930_g1_i1 | 80.31 | 8.00E-67 | 219 |
| AT5G57660.1 | c94930_g3_i1 | 49.26 | 1.00E-155 | 472 |
| AT1G47250.1 | c94930_g3_i2 | 48.6 | 1.00E-153 | 467 |
| AT1G47250.1 | c94930_g5_i1 | 82.26 | 6.00E-26 | 108 |
| AT2G28550.3 | c119742_g1_i1 | 51.38 | 6.00E-24 | 99.4 |
| AT2G28550.3 | c52913_g1_i1 | 77.27 | 2.00E-69 | 224 |
| AT2G28550.3 | c52913_g1_i2 | 85.71 | 7.00E-83 | 263 |
| AT2G28550.3 | c61024_g1_i2 | 85.48 | 6.00E-31 | 118 |
| AT2G28550.3 | c75317_g1_i1 | 53.44 | 3.00E-32 | 127 |
| AT2G28550.3 | c85378_g3_i1 | 50 | 5.00E-24 | 104 |
| AT2G28550.3 | c85378_g3_i2 | 51.15 | 2.00E-26 | 110 |
| AT2G28550.3 | c85378_g3_i3 | 49.39 | 2.00E-34 | 136 |
| AT2G28550.3 | c87192_g1_i1 | 92 | 2.00E-31 | 119 |
| AT2G28550.3 | c87192_g1_i3 | 57.55 | 2.00E-57 | 198 |
| AT2G28550.3 | c87192_g1_i5 | 91.23 | 8.00E-28 | 109 |
| AT2G28550.3 | c87192_g2_i2 | 55.14 | 3.00E-42 | 157 |
| AT2G28550.3 | c87192_g2_i3 | 47.57 | 1.00E-28 | 117 |
| AT2G28550.3 | c87192_g2_i4 | 46.2 | 4.00E-27 | 114 |
| AT2G28550.3 | c87192_g4_i1 | 85.25 | 6.00E-59 | 194 |
| AT2G28550.3 | c87192_g5_i1 | 57.55 | 7.00E-65 | 215 |
| AT2G28550.3 | c87192_g5_i2 | 51.48 | 2.00E-51 | 132 |
| AT2G28550.3 | c87192_g5_i3 | 89.13 | 3.00E-25 | 91.3 |
| AT2G28550.3 | c87192_g5_i4 | 86.05 | 1.00E-50 | 132 |
| AT2G28550.3 | c87192_g5_i5 | 89.13 | 9.00E-47 | 92 |
| AT2G28550.3 | c87982_g1_i1 | 53.33 | 3.00E-41 | 159 |
| AT2G28550.3 | c87982_g1_i2 | 53.33 | 3.00E-41 | 159 |
| AT2G28550.3 | c87982_g1_i5 | 51.9 | 3.00E-36 | 145 |
| AT2G28550.3 | c91054_g4_i3 | 54.96 | 2.00E-31 | 126 |
| AT2G28550.3 | c91054_g4_i4 | 53.66 | 3.00E-40 | 153 |
| AT2G28550.3 | c91054_g5_i1 | 53.66 | 4.00E-41 | 156 |
| AT2G28550.3 | c91085_g1_i4 | 47.24 | 9.00E-22 | 100 |
| AT2G28550.3 | c96694_g1_i1 | 51.83 | 5.00E-32 | 132 |
| AT2G28550.3 | c96694_g1_i4 | 50 | 7.00E-24 | 105 |
| AT2G28550.3 | c96979_g5_i2 | 50 | 3.00E-28 | 120 |
| AT2G28550.3 | c98453_g1_i1 | 73.33 | 1.00E-62 | 206 |
| AT2G28550.3 | c98453_g2_i1 | 71.11 | 5.00E-68 | 221 |
| AT1G68840.1 | c98453_g2_i2 | 59.4 | 1.00E-101 | 324 |
| AT2G28550.3 | c98453_g2_i3 | 63.92 | 6.00E-98 | 313 |
| AT1G68840.1 | c98453_g2_i4 | 59.4 | 3.00E-101 | 324 |
| AT1G69120.1 | c98453_g2_i5 | 59.73 | 2.00E-105 | 334 |
| AT2G28550.3 | c98453_g2_i6 | 88.64 | 5.00E-48 | 166 |
| AT2G28550.3 | c98453_g2_i7 | 63.69 | 3.00E-98 | 314 |
| AT5G03840.1 | c98453_g2_i8 | 59.4 | 1.00E-101 | 324 |
| AT5G67180.1 | c107136_g1_i1 | 88.31 | 4.00E-32 | 119 |
| AT5G67180.1 | c85625_g1_i1 | 49.66 | 5.00E-31 | 124 |
| AT5G67180.1 | c91085_g1_i2 | 51.33 | 2.00E-21 | 97.8 |
| AT5G67180.1 | c91085_g1_i3 | 51.33 | 3.00E-21 | 97.8 |
| AT5G67180.1 | c91085_g1_i5 | 51.33 | 2.00E-21 | 97.8 |
| AT5G67180.1 | c91085_g1_i6 | 39.13 | 3.00E-22 | 100 |
| AT5G67180.1 | c91085_g1_i7 | 39.13 | 3.00E-22 | 100 |
| AT2G45660.1 | c96979_g1_i1 | 52.24 | 1.00E-32 | 123 |
| AT2G45660.1 | c96979_g5_i1 | 50.28 | 2.00E-40 | 154 |
| AT4G11110.1 | c96979_g6_i1 | 51.4 | 1.00E-42 | 160 |
| AT5G44080.1 | c96979_g6_i2 | 51.4 | 1.00E-42 | 160 |
| AT3G10390.1 | c135320_g1_i1 | 70.83 | 4.00E-31 | 112 |
| AT2G37060.1 | c44764_g1_i1 | 69.71 | 5.00E-83 | 251 |
| AT4G14540.1 | c44764_g1_i2 | 72.73 | 6.00E-41 | 140 |
| AT1G65480.1 | c91926_g8_i1 | 66.86 | 2.00E-80 | 243 |
| AT5G65430.3 | c91926_g8_i2 | 55.88 | 5.00E-73 | 226 |
| AT4G30200.2 | c1071_g1_i1 | 61.11 | 7.00E-30 | 118 |
| AT4G30200.2 | c13767_g1_i1 | 50.92 | 1.00E-45 | 162 |
| AT4G30200.2 | c58996_g1_i1 | 36.03 | 5.00E-20 | 93.2 |
| AT2G19520.1 | c87738_g1_i1 | 47.56 | 0 | 565 |
| AT3G04610.1 | c87738_g1_i2 | 47.56 | 0 | 565 |
| AT4G30200.2 | c92401_g12_i1 | 60.19 | 5.00E-33 | 126 |
| AT2G37060.1 | c92401_g3_i2 | 41.57 | 3.00E-101 | 335 |
| AT2G28550.3 | c92401_g3_i3 | 43.43 | 4.00E-133 | 412 |
| AT4G30200.2 | c92401_g6_i1 | 60.84 | 1.00E-47 | 168 |
| AT4G30200.2 | c92401_g6_i2 | 65.96 | 4.00E-27 | 112 |
| AT4G02560.1 | c92401_g10_i1 | 66.67 | 3.00E-36 | 135 |
| AT3G44460.1 | c142691_g1_i1 | 56.79 | 2.00E-26 | 105 |
| AT3G18990.1 | c44358_g2_i2 | 34.71 | 3.00E-22 | 95.1 |
| AT3G18990.1 | c85305_g2_i6 | 26.28 | 4.00E-20 | 93.2 |
| AT5G63470.1 | c88187_g1_i1 | 42.5 | 2.00E-25 | 110 |
| AT5G57660.1 | c89643_g1_i1 | 56.1 | 1.00E-38 | 147 |
| AT3G18990.1 | c98995_g1_i3 | 42.4 | 2.00E-24 | 102 |
| AT1G77300.1 | c98995_g1_i5 | 32.14 | 9.00E-42 | 155 |
| AT3G18990.1 | c98995_g1_i6 | 40.29 | 2.00E-24 | 102 |
| AT3G24440.1 | c140400_g1_i1 | 64.56 | 1.00E-29 | 115 |
| AT5G51230.1 | c93433_g1_i1 | 50.51 | 2.00E-166 | 509 |
| AT4G02560.1 | c93433_g1_i2 | 50.51 | 4.00E-166 | 509 |
| AT4G02560.1 | c93433_g1_i3 | 50.51 | 2.00E-168 | 509 |
| AT3G24440.1 | c93433_g1_i4 | 50.51 | 1.00E-165 | 509 |
| AT4G02560.1 | c93433_g1_i5 | 50.51 | 2.00E-166 | 509 |
| AT3G24440.1 | c93433_g2_i1 | 48.86 | 2.00E-157 | 478 |
| AT3G24440.1 | c93433_g2_i2 | 48.86 | 1.00E-157 | 478 |
| AT4G11880.1 | c109817_g1_i1 | 73.03 | 4.00E-41 | 147 |
| AT5G67380.1 | c75781_g1_i1 | 92.05 | 2.00E-78 | 255 |
| AT3G04610.1 | c88070_g1_i1 | 84.66 | 0 | 915 |
| AT2G43410.2 | c88070_g1_i2 | 84.66 | 0 | 915 |
| AT5G57360.2 | c88070_g2_i1 | 91.18 | 4.00E-34 | 127 |
| LOC_Os02g46450.1 | c106297_g1_i1 | 54.48 | 3E-45 | 164 |
| LOC_Os01g72220.1 | c112445_g1_i1 | 26.74 | 1E-21 | 95.5 |
| LOC_Os12g05590.1 | c141582_g1_i1 | 73.33 | 3E-25 | 96.7 |
| LOC_Os05g03040.1 | c31752_g1_i1 | 43.65 | 3E-21 | 93.2 |
| LOC_Os03g17570.1 | c39294_g1_i1 | 55.15 | 1E-22 | 100 |
| LOC_Os10g32600.1 | c45692_g1_i1 | 38.06 | 7E-24 | 95.5 |
| LOC_Os01g16414.1 | c49456_g1_i1 | 36.72 | 3E-23 | 99.8 |
| LOC_Os01g16414.1 | c49456_g2_i1 | 30.21 | 9E-26 | 108 |
| LOC_Os05g47650.1 | c54446_g1_i1 | 68.25 | 2E-20 | 89.7 |
| LOC_Os12g13030.1 | c59813_g1_i1 | 69.7 | 2E-28 | 108 |
| LOC_Os02g34850.1 | c60881_g1_i1 | 36.53 | 9E-24 | 102 |
| LOC_Os07g13170.1 | c61024_g1_i1 | 85.54 | 3E-36 | 132 |
| LOC_Os05g49590.2 | c64106_g1_i1 | 56.25 | 1E-52 | 186 |
| LOC_Os07g02350.1 | c66299_g1_i1 | 88.24 | 9E-36 | 55.1 |
| LOC_Os08g36790.1 | c67037_g1_i1 | 32.54 | 5E-33 | 130 |
| LOC_Os07g41580.1 | c68387_g1_i1 | 79.63 | 4E-57 | 186 |
| LOC_Os01g16414.1 | c70813_g1_i2 | 25 | 5E-24 | 107 |
| LOC_Os07g41580.1 | c72831_g1_i1 | 82 | 9E-23 | 95.9 |
| LOC_Os07g41580.1 | c72831_g2_i1 | 72.73 | 1E-24 | 98.2 |
| LOC_Os10g39130.1 | c75036_g1_i2 | 64.06 | 1E-20 | 91.7 |
| LOC_Os06g47890.1 | c76059_g1_i1 | 38.81 | 1E-21 | 98.6 |
| LOC_Os09g30310.1 | c78473_g1_i1 | 84.04 | 1E-49 | 171 |
| LOC_Os07g48570.1 | c79151_g1_i1 | 33.33 | 4E-37 | 143 |
| LOC_Os12g40560.2 | c81479_g1_i1 | 79.1 | 6E-26 | 102 |
| LOC_Os07g41580.1 | c81528_g3_i1 | 76.42 | 8E-54 | 175 |
| LOC_Os08g44510.1 | c82061_g1_i1 | 27.06 | 2E-32 | 138 |
| LOC_Os10g26620.1 | c83407_g1_i3 | 63.79 | 2E-21 | 95.5 |
| LOC_Os07g41370.1 | c83742_g1_i1 | 42.33 | 1E-38 | 141 |
| LOC_Os09g30310.1 | c84395_g3_i2 | 82.11 | 7E-48 | 166 |
| LOC_Os02g46450.1 | c84580_g2_i1 | 55.26 | 5E-45 | 178 |
| LOC_Os07g49460.2 | c85628_g7_i1 | 47.54 | 1E-21 | 96.7 |
| LOC_Os07g49460.2 | c85628_g8_i1 | 53.6 | 3E-21 | 94.4 |
| LOC_Os10g26620.1 | c86263_g1_i2 | 65.67 | 5E-24 | 104 |
| LOC_Os01g72220.1 | c86665_g1_i1 | 26.64 | 3E-22 | 103 |
| LOC_Os01g72220.1 | c86665_g1_i2 | 26.64 | 3E-22 | 103 |
| LOC_Os01g72220.1 | c86665_g1_i3 | 26.64 | 3E-22 | 103 |
| LOC_Os07g13170.1 | c87192_g3_i2 | 37.37 | 5E-24 | 104 |
| LOC_Os08g01054.1 | c87341_g1_i1 | 32.37 | 3E-23 | 110 |
| LOC_Os07g13170.1 | c87982_g1_i3 | 54.64 | 8E-20 | 91.7 |
| LOC_Os07g02350.1 | c89026_g1_i1 | 31.82 | 5E-34 | 133 |
| LOC_Os01g64730.1 | c8912_g1_i1 | 60.47 | 1E-26 | 103 |
| LOC_Os02g04100.1 | c89728_g3_i2 | 36 | 1E-31 | 124 |
| LOC_Os11g05930.1 | c91063_g2_i2 | 38.52 | 6E-20 | 96.7 |
| LOC_Os01g17000.1 | c92107_g1_i1 | 56.32 | 4E-20 | 90.1 |
| LOC_Os08g44510.1 | c92144_g7_i1 | 71.79 | 4E-25 | 61.6 |
| LOC_Os08g44510.1 | c92144_g7_i2 | 71.79 | 2E-28 | 61.6 |
| LOC_Os10g32600.1 | c92222_g4_i3 | 36.77 | 4E-21 | 88.6 |
| LOC_Os03g39129.1 | c92613_g2_i1 | 28 | 2E-21 | 102 |
| LOC_Os09g03610.1 | c92711_g5_i1 | 51.85 | 9E-21 | 93.2 |
| LOC_Os03g10940.1 | c93001_g1_i1 | 29.79 | 2E-22 | 99 |
| LOC_Os03g10940.1 | c93001_g2_i1 | 29.79 | 1E-21 | 99 |
| LOC_Os03g10940.1 | c93390_g4_i7 | 29.21 | 8E-20 | 90.5 |
| LOC_Os10g32600.1 | c93556_g1_i1 | 38.27 | 5E-24 | 102 |
| LOC_Os10g32600.1 | c93556_g1_i3 | 38.27 | 5E-24 | 102 |
| LOC_Os08g04290.1 | c94134_g3_i1 | 66.18 | 9E-28 | 109 |
| LOC_Os08g04290.1 | c94134_g6_i2 | 61.58 | 1E-77 | 246 |
| LOC_Os10g28330.1 | c94499_g1_i1 | 79.25 | 1E-20 | 93.2 |
| LOC_Os08g01054.1 | c96436_g2_i1 | 48.44 | 2E-50 | 198 |
| LOC_Os04g47270.1 | c96822_g1_i1 | 31.22 | 2E-40 | 160 |
| LOC_Os01g51610.1 | c97066_g3_i3 | 39.62 | 7E-20 | 94.4 |
| LOC_Os10g26620.1 | c97714_g7_i1 | 73.08 | 6E-21 | 97.4 |
| LOC_Os08g33370.1 | c98000_g7_i3 | 88 | 8E-20 | 91.7 |
| LOC_Os01g70810.1 | c98104_g4_i1 | 60.76 | 1E-23 | 100 |
| LOC_Os07g02350.1 | c98248_g2_i1 | 37.19 | 4E-21 | 90.5 |
| LOC_Os01g67970.1 | c98404_g1_i1 | 53.61 | 1E-26 | 109 |
| LOC_Os02g34850.1 | c98840_g1_i1 | 36.81 | 3E-29 | 120 |
| LOC_Os02g34850.1 | c99621_g4_i9 | 39.37 | 8E-20 | 97.8 |
